# Resolving the relationships of Paleocene placental mammals

**DOI:** 10.1111/brv.12242

**Published:** 2015-12-21

**Authors:** Thomas J. D. Halliday, Paul Upchurch, Anjali Goswami

**Affiliations:** ^1^ Department of Earth Sciences University College London Gower Street London WC1E 6BT U.K.; ^2^ Department of Genetics, Evolution and Environment University College London Gower Street London WC1E 6BT U.K.

**Keywords:** Placentalia, evolution, phylogeny, Palaeogene, radiation, Condylarthra, palaeontology

## Abstract

The ‘Age of Mammals’ began in the Paleocene epoch, the 10 million year interval immediately following the Cretaceous–Palaeogene mass extinction. The apparently rapid shift in mammalian ecomorphs from small, largely insectivorous forms to many small‐to‐large‐bodied, diverse taxa has driven a hypothesis that the end‐Cretaceous heralded an adaptive radiation in placental mammal evolution. However, the affinities of most Paleocene mammals have remained unresolved, despite significant advances in understanding the relationships of the extant orders, hindering efforts to reconstruct robustly the origin and early evolution of placental mammals. Here we present the largest cladistic analysis of Paleocene placentals to date, from a data matrix including 177 taxa (130 of which are Palaeogene) and 680 morphological characters. We improve the resolution of the relationships of several enigmatic Paleocene clades, including families of ‘condylarths’. *Protungulatum* is resolved as a stem eutherian, meaning that no crown‐placental mammal unambiguously pre‐dates the Cretaceous–Palaeogene boundary. Our results support an Atlantogenata–Boreoeutheria split at the root of crown Placentalia, the presence of phenacodontids as closest relatives of Perissodactyla, the validity of Euungulata, and the placement of Arctocyonidae close to Carnivora. Periptychidae and Pantodonta are resolved as sister taxa, Leptictida and Cimolestidae are found to be stem eutherians, and Hyopsodontidae is highly polyphyletic. The inclusion of Paleocene taxa in a placental phylogeny alters interpretations of relationships and key events in mammalian evolutionary history. Paleocene mammals are an essential source of data for understanding fully the biotic dynamics associated with the end‐Cretaceous mass extinction. The relationships presented here mark a critical first step towards accurate reconstruction of this important interval in the evolution of the modern fauna.

## I. INTRODUCTION

The Cretaceous–Palaeogene (hereafter K/Pg) mass extinction represents one of the largest global ecological turnovers in the history of life. Occurring 66 million years ago, it was the second largest mass extinction of all time, during which some 75% of terrestrial species were extinguished (Jablonski & Chaloner, 1994), dramatically altering both terrestrial and marine ecosystems (Vajda, Raine & Hollis, 2001; Sessa *et al.*, 2012). Palaeontologists usually reconstruct this point as the beginning of the so‐called ‘Age of Mammals’; prior to the K/Pg boundary, mammals were mainly small, terrestrial‐to‐arboreal insectivores with low ecological disparity (Goswami, 2012; Grossnickle & Polly, 2013), albeit with a few notable exceptions (Luo, 2007). By contrast, Palaeogene mammals include the first large‐bodied herbivores, specialised carnivores, and later, radiations of gliding, flying, and fully aquatic organisms, with a corresponding increase in diversity (Darroch *et al.*, 2014).

This apparently sudden increase in ecospace occupation has been interpreted as an adaptive radiation, particularly in placental mammals (Osborn, 1902; Simpson, 1953; Alroy, 1999; Raia *et al.*, 2013). However, macroevolutionary studies of placental mammals of this period are limited by the lack of a comprehensive phylogeny for Paleocene placentals. With the exception of Primates (Russell, 1964), Rodentia (Jepsen, 1937), and Carnivora (Fox, Scott & Rankin, 2010), no extant order of placental mammal has an unambiguous representative during the Paleocene, minimally leaving a 10 million year gap between the K/Pg mass extinction and the origin of most extant orders. Pertinent to the question of when placental mammals diversified are the currently unresolved phylogenetic relationships of the majority of Paleocene mammals; they occur during the period of rapid ecological diversification for placental mammals, but pre‐date the definitive first appearances of most of the extant orders.

Many previous studies have assessed the timing of the origin of placental mammals (Bininda‐Emonds *et al.*, 2007; O'Leary *et al.*, 2013), or examined changes in rates of evolution of body size or diversification across the K/Pg boundary (Springer *et al.*, 2003; Venditti, Meade & Pagel, 2011; Slater, 2013). All, however, have used data sets that mostly or entirely excluded Paleocene taxa, and therefore lack data from the important period during which an adaptive radiation would seem, from a strict reading of the fossil record, to have occurred. These analyses, which have mostly used divergence estimates from molecular dating techniques, have tended to favour a ‘mid’ to Late Cretaceous origin of placental orders and superorders (Springer *et al.*, 2003; Bininda‐Emonds *et al.*, 2007; dos Reis *et al.*, 2012). However, despite numerous suggestions of Cretaceous placentals, no Cretaceous eutherian mammal has been unambiguously resolved within the placental crown (Wible *et al.*, 2009; Goswami *et al.*, 2011). The earliest definitive members of crown orders are mostly known from the Late Paleocene or Eocene. A Cretaceous origin would therefore require the existence of long ghost lineages. Additionally, it has been suggested that clock models suffer from artefacts resulting from historical changes in evolutionary rate (Beck & Lee, 2014). Estimating the date of origin of placental mammals and reconstructing their response to the end‐Cretaceous mass extinction are therefore highly contingent on method and data set.

Addition of fossil data has also been shown to change results of analyses significantly in a wide range of macroevolutionary studies (Tarver & Donoghue, 2011; Pyron & Burbrink, 2012; Slater, Harmon & Alfaro, 2012; Wood *et al.*, 2013; Raj Pant, Goswami & Finarelli, 2014). The inclusion of fossil data is, however, only possible where the phylogenetic relationships of those fossil forms is understood.

**Figure 1:**
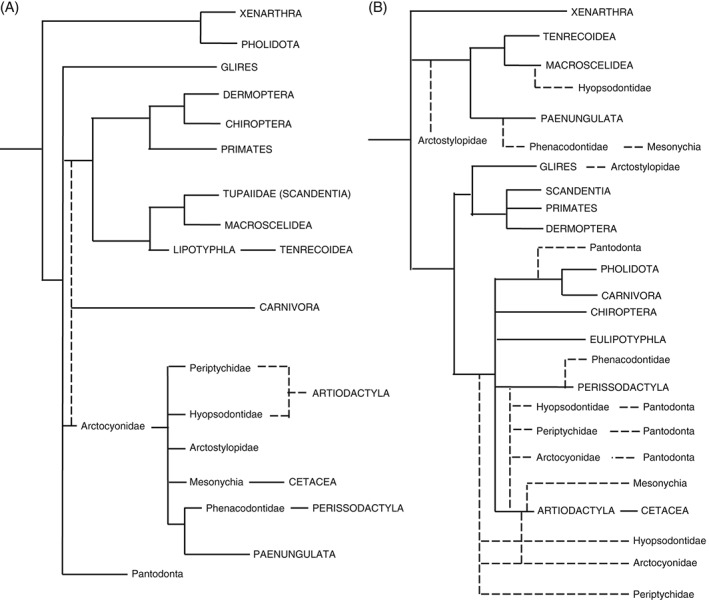
A depiction, in broad terms, of changes in understanding of the relationships of placental mammals over the last 20 years. (A) Broad understanding of placental mammal relationships prior to the advent of molecular data. ‘Condylarth’ families were considered basal to Ungulata, a number of plesiomorphic taxa were grouped together as Insectivora, whales were considered a separate order, and pangolins were joined with xenarthrans in Edentata. (B) The current consensus on placental mammal phylogeny. While the relationships of extant groups are clear – Cetacea is a subgroup of Artiodactyla, Carnivora and Pholidota are sister taxa, Ungulata and Insectivora are polyphyletic – the relationships of Paleocene taxa have become far more uncertain. In both, dotted lines represent uncertainty.

The phylogenetic relationships among extant placental mammals have a long history of study based on morphological data, with some degree of stability in tree topology for several decades (Gregory, 1910; Simpson, 1945; McKenna, 1975; Novacek, 1992). This traditional topology accommodated many of the Paleocene mammal clades in a relatively straightforward manner, such as ‘condylarths’ being identified as stem ungulates (Fig. 1A). However, towards the latter half of the 20th century, questions were raised about some of these groupings, such as the traditional clades of ‘Insectivora’ (insectivorous mammals) and ‘Ungulata’ (hoofed mammals) (see Asher, Geisler, & Sanchez‐Villagra, 2008 for a review of these). For instance, the distinction between the Afrotherian golden mole *Chrysochloris* and European moles was suggested, based on entirely morphological data, as early as the 19th century (Cope, 1884*c*). The advent of molecular sequencing and its application to mammalian phylogenetics confirmed the suggestions that Insectivora and Ungulata were polyphyletic (Stanhope *et al.*, 1998). With the division of Insectivora into Eulipotyphla and Afroinsectivora, and Ungulata into Perissodactyla, Artiodactyla, and Paenungulata, several fossil taxa were left without a well‐supported position in the placental tree of life. In particular, ‘Condylarthra’, historically thought to be ancestral to ‘Ungulata’, was reduced to the status of ‘wastebasket taxon’, into which any generically bunodont, unguligrade mammal from the Palaeogene has been consigned (Archibald, 1998). Patently, these taxa must have ancestors, and extant orders likely evolved from some of the ambiguous Paleocene taxa, but the nature of their relationships remains perplexing. Indeed, every ‘condylarth’ family‐level clade has been suggested to be related to one or more extant clade, with several hypotheses existing for each (Fig. 1B). For the most part, these ‘condylarth’ clades themselves are considered monophyletic, but the relationships among them, and between any ‘condylarth’ clade and extant orders are unknown.

Identifying the phylogenetic position of these enigmatic Palaeogene taxa with respect to extant orders and Cretaceous groups is therefore essential to understanding the timing of divergence of extant orders. Distinct Paleocene mammals first appear less than a million years after the K/Pg boundary, and if they are crown‐placental mammals, this timing would imply that speciation between extant orders most likely occurred cryptically during the Cretaceous. Establishing how many mammalian lineages span the K/Pg boundary also allows for greater accuracy in assessing the role of mass extinctions in evolutionary dynamics more generally. Ascertaining the phylogenetic relationships of fossil forms will facilitate robust, inclusive studies of character evolution that directly sample taxa from the relevant intervals and better represent true clade diversity. Ultimately, a phylogeny of Paleocene mammals is sorely needed, but has not been forthcoming, despite a great deal of energy directed towards study of the end‐Cretaceous mass extinction and its aftermath.

## II. AIMS

Here, we present the results of the largest cladistic analysis of Palaeogene mammals to date, with the aim of resolving the relationships of some enigmatic groups of eutherian mammals. Inclusion of key representative taxa from this important period in placental evolution in a wide‐reaching study is a crucial requirement for future analysis of the early evolution of this clade. Below, we provide a detailed overview of the early fossil record of placental mammals, followed by the new cladistic analysis and a discussion of its implications for resolving internal placental relationships and the origins of the group.

## III. OVERVIEW OF THE FOSSIL RECORD OF EARLY PLACENTAL MAMMALS

### (1) ‘Condylarthra’

By far the largest component of the mammalian biota in the Paleocene is the collection of ‘archaic ungulates’ known as ‘condylarths’. While this grouping is almost certainly an anachronistic grade of largely terrestrial, bunodont, herbivorous‐to‐omnivorous mammals, there are several well‐defined families which fall within ‘Condylarthra’.

#### (a) *Arctocyonidae*

##### (i) *Composition*

Arctocyonidae is composed of approximately 20 genera, including some of the best known of the ‘archaic ungulates’, such as the type species *Arctocyon primaevus*, discovered in 1841 (de Blainville, 1841), and the arboreal mammal *Chriacus*. Other genera include *Mentoclaenodon*, *Lambertocyon*, *Thryptacodon*, *Anacodon*, and *Claenodon*. It has been disputed whether *Claenodon* is considered a separate genus in its own right (Rose, 1981), a synonym of *Arctocyon* (Rigby, 1980), or a synonym of *Arctocyonides* (Van Valen, 1978). Some recent studies (Williamson & Carr, 2007; De Bast & Smith, 2013) have hinted that the subfamilies of Arctocyonidae may be polyphyletic, with the Oxyclaeninae occurring at the base of crown placentals (although all taxa sampled in those analyses are certain or probable laurasiatheres), Arctocyoninae as a sister group to the Mesonychia and Triisodontidae, and Loxolophinae closest to a broader clade comprising ancestors of Artiodactyla and Perissodactyla. According to De Bast & Smith (2013, p. 971), arctocyonids have ‘been used as a wastebasket for basal condylarths’; making arctocyonids particularly enigmatic, as the general consensus is that ‘Condylarthra’ is itself a wastebasket taxon (Archibald, 1998).

##### (ii) *Proposed relationships*

The arctocyonids are a primarily European group (Russell, 1964) which has variously been considered ancestral to Carnivora (Van Valen, 1969), specifically to miacid carnivorans (see Van Valen, 1978), to artiodactyls (Rose, 1996), and as part of the ancestral ungulate group (Kondrashov & Lucas, 2004; De Bast & Smith, 2013). Arctocyonidae was one of the two groups (the other being Oxyclaenidae) that was assigned to the most primitive groupings of ‘archaic ungulate’ – the Procreodi – by Matthew (1915). Simpson (1937) preferred to amalgamate Oxyclaenidae and Arctocyonidae, the latter taking precedence as the name for the whole group. McKenna & Bell (1997), however, placed Arctocyonidae within Procreodi once more, and divided the family into three main subgroups – the Arctocyoninae, Loxolophinae, and Oxyclaeninae. Some debate has concerned whether the particularly primitive but ungulate‐grade organism *Protungulatum*, excluded from Arctocyonidae by Prothero, Manning & Fischer (1988), should be included within the family.

##### (iii) *Ecology*

The species *Arctocyon primaevus* was originally described as being aquatic (de Blainville, 1841), and has been variously hypothesised as being terrestrial (Russell, 1964), fossorial (Kondrashov, 2009), and scansorial–arboreal (Argot, 2013) by subsequent authors. More consistent in interpretation, *Chriacus* is considered to be arboreal (Rose, 1987) on the basis of features of its tarsal bones, among others. The climbing of *Chriacus* is largely inferred from various skeletal features of an almost complete postcranial specimen, which includes all but portions of the femur and a number of vertebrae (Rose, 1987). The shape of the acromion process in *Chriacus*, combined with the extensive deltopectoral ridge of the humerus is indicative of powerful forelimb musculature, and the presence of large forefeet with curved claws suggests either climbing or burrowing ability. Comparisons may be made between the humerus of *Chriacus* and that of various arboreal carnivorans such as the coati, civets and red pandas (Rose, 1987). There is a great diversity of locomotor styles within Procreodi/Arctocyonidae; it has been suggested that some members of the group – *Arctocyon mumak* and *Anacodon* – might have been semifossorial, based on morphological features of the astragalus (Gould & Rose, 2014). In general, the arctocyonids possess teeth adapted for some degree of carnivory, even if it is not as extensive as that displayed by the creodonts and true carnivorans, lacking as they do any specialised shearing carnassials. Large canines are often present, and the premolars are relatively sharp, but the molars are bunodont, suggesting a largely omnivorous dietary niche.

#### (b) *Periptychidae*

##### (i) *Composition*

Periptychidae is composed of approximately 15 genera, divided among the subfamilies Periptychinae, Anisonchinae, and Conacodontinae (Archibald, Schoch & Rigby, 1983), the latter of which comprises *Conacodon* and *Oxyacodon*. According to Archibald *et al.* (1983), the most ‘primitive’ periptychid is *Mimatuta*, although Van Valen (1978) recognised five lineages of periptychids and suggested that they all descended directly from *Protungulatum*, the Cretaceous–Palaeogene proto‐ungulate‐grade eutherian.

##### (ii) *Proposed relationships*

Periptychids are represented by several North American genera, such as *Periptychus*, *Anisonchus*, *Ectoconus*, and others, and, in the case of *Periptychus*, are among the earliest known crown‐placental mammals (Prothero, 1998; Lofgren *et al.*, 2004). They first appear in the San Juan Basin as immigrants (Clemens, 2010; Wilson, 2014) approximately 500000 years after the end‐Cretaceous mass‐extinction event, and are, as a result, usually thought to be basal among ‘condylarths’ (Prothero, 1994).

##### (iii) *Ecology*

Periptychid condylarths are characterised by highly bunodont, square molars which are of roughly equal size along the tooth row. As they are considered to be both temporally early and phylogenetically basal, they show the first adaptations to a herbivorous lifestyle.

#### (c) *Hyopsodontidae*

##### (i) *Composition*

Depending on interpretation, Hyopsodontidae may be treated as synonymous with Mioclaenidae (Williamson & Weil, 2011). Some authors prefer Mioclaenidae to be an entirely separate group, an interpretation backed up with some cladistic evidence (Ladevèze, Missiaen & Smith, 2010). In that analysis, both groups were represented by only a single genus, and the sample size of the entire analysis was not sufficient to draw an informed judgement on the relative positions of all ‘condylarth’ lineages. In total, there are 15 genera of hyopsodontid. The type genus, *Hyopsodus* is primarily Eocene, and is found across all Laurasian land masses.

##### (ii) *Proposed relationships*

Hyopsodontids are typical of the archaic ungulate families in that they have been considered ancestral to many different groups of ungulate‐grade mammal. Traditionally, they were considered to be early ancestors of artiodactyls (Simpson, 1937; Schaeffer, 1947), but later hypotheses placed them with either afrotheres – whether hyracoids (Godinot, Smith & Smith, 1996) or macroscelideans (Tabuce *et al.*, 2001) – or with the enigmatic South American ungulates (Cifelli, 1983; de Muizon & Cifelli, 2000). The characters that have linked hyopsodontids to this large variety of clades are mostly dental, although in the case of the macroscelidean relationships this depends on the assumption that apheliscid ‘condylarths’ fall within Hyopsodontidae, and is supported primarily by the morphology of the tarsals. Since both teeth and tarsals have morphologies that are highly tied to their ecology (diet and locomotor ability, respectively), the conflicting evidence suggests that hyopsodontids are convergent in one or both of these regions. This is problematic when there is little in the way of postcranial remains of hyopsodontid ‘condylarths’.

##### (iii) *Ecology*

Hyopsodontid condylarths are one of the most widespread groups of archaic ungulate. They are found from the Middle Paleocene, with the earliest representatives found across North and South America. Eocene representatives are known from Europe and Asia, and they are a remarkably cosmopolitan group until they disappear during the Eocene (Hooker & Dashzeveg, 2003).

Analysis of the braincase of *Hyopsodus lepidus* has indicated that it possessed strong abilities to detect the positions of acoustic stimuli accurately, due to an enlarged inferior colliculus. This has been interpreted as implying an ability to echolocate in a similar way to some burrowing and nocturnal shrews and tenrecs (Orliac, Argot & Gilissen, 2012*a*). However, because the postcranium of *Hyopsodus* is not strongly adapted for digging, and as the genus is more usually reconstructed as a scansorial herbivore (Williamson & Lucas, 1992), the more likely interpretation is of at least a nocturnal habit, although some digging ability – as well as the possibility of living in vacated burrows – cannot be discounted.

#### (d) *Mioclaenidae*

##### (i) *Composition*

Those that subscribe to the viewpoint that mioclaenids are a separate family place all 10 mioclaenid genera in the subfamily Mioclaeninae (e.g. Zack *et al.*, 2005*b*). Regardless of phylogenetic topology, mioclaenids are considered by all to be monophyletic.

##### (ii) *Proposed relationships*

Mioclaenidae are a little known group of archaic ungulates considered by some to be a subgroup or synonym of Hyopsodontidae (Williamson & Weil, 2011). Originally erected as a monospecific family (Osborn & Earle, 1895), it includes several taxa from across North America and Europe, as well as a few in South America.

##### (iii) *Ecology*

The most recent summary of the characters which define Mioclaenidae was a reanalysis of two genera – *Bomburia* and *Ellipsodon* – by Williamson & Carr (2007), where the family was rediagnosed as 'Ungulate with P4 metacone absent, upper molar postcingulum continuous with metastyle, lower molar metaconid nearly lingual to protoconid, ratio of m3 length/m2 length between 0.9 and 1.1'. Lack of a metacone aside, these traits are relatively primitive for a large number of groups of ‘archaic ungulates’ (see Prothero *et al.*, 1988). Mioclaenids also have relatively enlarged premolars, similar to periptychids, which are thought to indicate a diet consisting of tough vegetation (Rose, 2006).

#### (e) *Pleuraspidotheriidae*

##### (i) *Composition*

Pleuraspidotheriidae is a small group sometimes placed within Meniscotheriidae, Hyopsodontidae or Phenacodontidae, and recently affined to the early arctocyonids. It is exclusively found in Europe, mainly in northern France and Belgium, and consists of three genera – *Pleuraspidotherium*, *Orthaspidotherium* and the enigmatic Turkish fossil *Hilalia*.

##### (ii) *Proposed relationships*

Ladevèze *et al.* (2010) hypothesised that Pleuraspidotheriidae are closest relatives to arctocyonids such as *Chriacus*, in a group also including the basal artiodactyls, but their taxonomic sampling was very low, and only very few representatives of each supposed group were present. Since the majority of the ‘condylarth’ material has been collected from North America, or are parts of families that are present in North America with representatives elsewhere, the phylogenetic position of a clade from another continent is of interest when considering biogeographic questions regarding the origin of modern orders, and little has been proposed concerning the relationships of this family.

##### (iii) *Ecology*

The basicranial morphology of pleuraspidotheres is similar to that of the early artiodactyls such as *Gobiohyus*, their teeth resemble the previously mentioned ‘condylarth’ groups, and their tarsal morphology is basal in appearance, with little in the way of unambiguous synapomorphies. Preservation of the two better‐known genera is very good, with an almost complete skeleton known for *Pleuraspidotherium*, and a complete skull with assorted postcranial material known for *Orthaspidotherium* (Ladevèze *et al.*, 2010). The pseudohypocone that characterised the square molars of the Pleuraspidotheriidae mark the difference between this morphology and the superficially similar molars of perissodactyls (Ladevèze *et al.*, 2010).

#### (f) *Phenacodontidae*

##### (i) *Composition*

Phenacodontidae is composed of 14 North American genera, the best known and most complete of which are the phenacodontine phenacodontids *Phenacodus*, *Tetraclaenodon* and *Copecion* (Osborn, 1898; Thewissen, 1990; Kondrashov & Lucas, 2012). Meniscotheriidae, once considered separate, is now generally included within Phenacodontidae (Rose, 2006) as the subfamily Meniscotheriinae (Simpson, 1937), and includes *Ectocion* and *Meniscotherium*.

##### (ii) *Proposed relationships*

Like the apheliscid ‘condylarths’, phenacodontids have been suggested to be closely related to afrotherian and laurasiatherian orders. In particular, Phenacodontidae was resolved by Tabuce *et al.* (2001) as being part of a clade comprising Paenungulata, Phenacodontidae and Perissodactyla, while Kondrashov & Lucas (2012) found phenacodontids to be paraphyletic to Perissodactyla, Paenungulata, and Hyracoidea. While Perissodactyla is certainly not closely related to Paenungulata, being consistently resolved as being in a totally different superorder by molecular methods (Springer *et al.*, 2004), Phenacodontidae represent the phenotype that was previously thought to link the two groups ancestrally. It is not clear whether support for the affinity was driven largely by one order or another – both Paenungulata (e.g. Rose *et al.*, 2014) and Perissodactyla (e.g. Kondrashov & Lucas, 2012) have been considered the closest living relatives of phenacodontids in recent years, whether phenacodontids are considered paraphyletic or monophyletic. Indeed, *Phenacodus* and *Meniscotherium* have also been found to be close relatives of Artiodactyla (Wible *et al.*, 2007).

##### (iii) *Ecology*

Phenacodontids are superficially similar to the modern groups of ungulate mammals, with a herbivorous diet, and generalised, often slightly cursorial limbs, especially in more derived forms (Thewissen, 1990). Upper molars are bunodont and square, with the presence of a hypocone being relatively derived. Lower molars, however, are reduced in the number of cusps, with the paraconid having been lost. The forelimb of *Tetraclaenodon* has been described as having features associated with both terrestriality and climbing, although these attributes are weakly developed, and it has been suggested that *Tetraclaenodon* behaved in such a way that it was facultatively terrestrial, but able to scale trees for food or safety (Kondrashov & Lucas, 2012). This hypothesis is borne out by the morphology of the hind limb, which is far more specialised for terrestriality, although not cursoriality (Kondrashov & Lucas, 2012). The third trochanter on the femur is a cursorial adaptation, as is the weakening of the deltopectoral crest of the humerus.

#### (g) *South American native ungulates (SANUs)*

##### (i) *Composition*

The placental fauna of South America, with the exception of the native xenarthrans and later invasions of African and North American groups, include three to five orders of ‘ungulate’ (McKenna, 1975). These orders – Xenungulata, Notoungulata, Litopterna, and, if they are considered separate, Pyrotheria and Astrapotheria – are highly enigmatic with respect to their relationships with extant placental orders. They first appear in South America during the Paleocene (de Muizon & Cifelli, 2000), surviving into the Late Pleistocene (MacFadden & Shockey, 1997). Across all five orders, there are well over 200 named genera, many of which are known from multiple species (McKenna & Bell, 1997).

##### (ii) *Proposed relationships*

Although some hypotheses have suggested that SANUs are more closely related to Afrotheria (Agnolin & Chimento, 2011), or descended from ‘condylarths’ (de Muizon & Cifelli, 2000), recent analysis of protein sequences from subfossil material (Buckley, 2015; Welker *et al.*, 2015) has indicated that the closest extant relatives of both Notoungulata (represented by *Toxodon*) and Litopterna (represented by *Macrauchaenia*) are stem Perissodactyla, a result which is consistent with a recent morphological analysis incorporating Notoungulata (Beck & Lee, 2014), which returned close relationships between notoungulates and perissodactyls. As morphological analyses have been inconsistent in terms of the relationships of these unusual taxa, this particular topology raises many biogeographical questions. Cladistic analysis of the morphology of SANU postcrania has supported a relationship between a notoungulate–litoptern clade and phenacodontids (Horovitz, 2004), with astrapotheres most closely related to periptychids. Relationships among orders of SANU are unclear, with Billet (2010) finding a notoungulate–astrapothere clade and non‐monophyletic Litopterna.

##### (iii) *Ecology*

Ecologically, the SANUs are remarkably diverse, with analogues of several artiodactyl and perissodactyl clades, most clearly emphasised in the similarity between litopterns, artiodactyl camelids, and perissodactyl equids (Bond *et al.*, 2006). It is their especially derived morphology and geographical isolation that presents problems when determining their closest relatives, despite a relatively good fossil record from the Late Paleocene onwards.

### (2) Other placental non‐ungulate clades

In addition to the condylarths, there are several other controversial and enigmatic mammal groups represented by Paleocene fossils. These include two groups, Leptictida and Cimolestidae, variously considered to be stem to the placental lineage, or ancestral to an extant order or group of orders (Lopatin, 2006; Wible *et al.*, 2007). Both show relatively basal general morphology, but also bear specialisations that have driven hypotheses of relationships to extant clades.

#### (a) *Leptictida*

##### (i) *Composition*

Leptictida was first identified as a superorder by McKenna (1975), in which it was proposed to be a clade of crown‐group placental mammals with unclear affinities, whose closest relatives were the Kennalestidae. Novacek (1986) provided an extensive and comprehensive morphological characterisation of the group, reduced the rank to ordinal status, and proposed a position within Insectivora. In McKenna & Bell (1997), Leptictida was a diverse assemblage of taxa including several additional Cretaceous mammals such as *Zhelestes*, *Gypsonictops*, *Lainodon* and *Gallolestes*, and also Kennalestidae. The traditional leptictid forms such as *Prodiacodon* and *Leptictis* were also included in Leptictida, as well as the European Pseudorhynchocyonidae.

In light of further analysis, the taxonomic composition of Leptictida has been revised as a more restricted group. Archibald, Averianov & Ekdale (2001) provided evidence that *Gypsonictops* was part of a separate clade from *Zhelestes* and its kin, implying that Leptictida was at least paraphyletic. In that study, both clades were resolved within the placental crown, with *Gypsonictops* closer to Glires, and *Zhelestes* to Ungulata (represented in this case solely by *Protungulatum*, whose placement as a crown ungulate is questionable). Kielan‐Jaworowska, Cifelli & Luo (2004) maintained the presence of Gypsonictopidae in Leptictida, but considered that Leptictida was, as had originally been suggested, within Insectivora, as a sister group to Lipotyphla. The composition of Gypsonictopidae was also reduced by the removal of *Zhelestes*, as well as other forms previously allied to Leptictida such as *Lainodon*. The newly constructed Zhelestidae was allied with Ungulatomorpha, well within the crown of placental mammals.

An extensive study of Cretaceous mammalian affinities by Wible *et al.* (2007) further modified the position of Leptictida. Here, rather than being crown‐group placental mammals, *Leptictis* and *Gypsonictops* were placed at the crownward end of the placental stem, more derived than Zalambdalestidae but less than *Protungulatum*. Meehan & Martin (2010), however, favoured inclusion of Leptictida in the abandoned grouping ‘Insectivora’. They noted that the morphology of leptictidans was highly convergent to that of extant macroscelideans, due to similar ecological specialisations to insectivory, digging, and saltatory locomotion.

Recently, the European Eocene–Miocene Pseudorhynchocyonidae – a subgroup of Leptictida *sensu* McKenna & Bell (1997) – has been separated from Leptictida entirely, instead placed closer to Palaeanodonta and Pantodonta (Hooker, 2013). However, an unrooted version of the tree from this analysis is entirely consistent with a monophyletic Leptictida to the exclusion of Palaeanodonta and Pantodonta. The order Leptictida currently consists of three families: Gypsonictopidae, a monogeneric family containing only *Gypsonictops*; Leptictidae, consisting of several North American genera; and Pseudorhynchocyonidae. In total, the clade contains 16 genera. Although there is largely a consensus on the taxonomic composition of Leptictida, the precise position of their relationships to extant orders of mammals remains under question, and they occupy a crucial position in the temporal story of eutherian mammal evolution.

##### (ii) *Proposed relationships*

The leptictids are one of the few orders of mammals definitively to cross the K/Pg boundary. A few representatives from the Cretaceous, such as *Gypsonictops*, hint at an early branching from the placental mammal tree, although some analyses have preferred to place them within the crown (Kielan‐Jaworowska *et al.*, 2004; Meehan & Martin, 2010). Leptictida, therefore, are one of the key groups for understanding the timescale of placental evolution. Their presence on both sides of the K/Pg boundary means that, were they to fall within the placental radiation, it would provide conclusive proof of the early (pre K/Pg) origin of placental mammals.

The initial discovery of *Leptictis haydeni* was in Dakota, identified along with *Ictops dakotensis* (now known as *Leptictis dakotensis*) as two genera of ‘insectivorous mammals, which appear to be peculiar, but related to the hedge‐hogs’ (Leidy, 1868, p. 315). Leidy placed them within the order Insectivora, and they were first identified as a separate family with the name Leptictidae by Gill (1872).

##### (iii) *Ecology*

Leptictida are a specialised Laurasian group, occurring throughout northern North America from the Cretaceous to the Oligocene, with some examples – the Mongolian *Praolestes* (Matthew, Granger & Simpson, 1929), European pseudorhynchocyonids such as *Pseudorhynchocyon* (Filhol, 1892), and a Spanish specimen of *Leptictis* (Crusafont‐Pairo & Golpe Posse, 1975) – from the Eocene of Europe and Paleocene of Asia. They are characterised by their long hind limbs, superficially resembling jerboas and sengis, although this is probably an entirely convergent adaptation to a similar ecological niche of a hopping insectivore–omnivore (Rose, 1999*b*).

#### (b) *Cimolestidae*

##### (i) *Composition*

There are 13 genera within Cimolestidae, seven of which are monospecific. Five species of *Cimolestes* (*C. magnus, C. cerberoides, C. incisus, C. stirtoni,* and *C. propalaeoryctes*), as well as *Batodon tenuis* and *Maelestes gobiensis* are found in the Cretaceous – the former two in North America, and the latter in Mongolia, spanning the Judithian and Lancian North American faunal stages (83.3 to 65.5 Ma). Of these, *Cimolestes* is unusual in that it is a genus spanning the K/Pg boundary, and is found in the Puercan of North America, equivalent‐aged rocks in Bolivia, and the Thanetian of Morocco. Nonetheless, it must be pointed out that the monophyly of *Cimolestes* has at times been questioned. Paleocene and Cretaceous forms of *Cimolestes* may not be the same genus, with some concluding that Carnivora and Creodonta were independently derived from *Cimolestes* (Lillegraven, 1969; McKenna, 1975).

##### (ii) *Proposed relationships*

The cimolestids are a second group whose phylogenetic placement should inform strongly on the date of origin of the major clades of placental mammals. They too are hypothesised to occupy a variety of phylogenetic positions, as well as crossing the K/Pg boundary. It is disputed whether the Pantodonta are part of this clade (see differences between McKenna & Bell, 1997; Wible *et al.*, 2007), but even excluding the pantodonts, the cimolestids are a highly diverse and probably monophyletic lineage (Archibald, 2011).

The Cretaceous cimolestids include only the three genera mentioned above. By the earliest Paleocene, the group had diversified to include the South American *Alcidedorbignya*, and the Laurasian *Puercolestes*. Some also include the Paleocene taxon *Procerberus* in the cimolestid lineage (Williamson, Weil & Standhardt, 2011), although *Procerberus* has also been considered to be a very basal eutherian (Kielan‐Jaworowska, Bown & Lillegraven, 1979), and still others favour a relationship with Leptictida (Sloan & Van Valen, 1965).

Considering *Procerberus* as a stem eutherian mammal need not necessarily remove *Procerberus* from the cimolestids, however, because evidence has suggested that cimolestids might be a group of stem placental mammals as well, although others have likened them to the hypothesised ancestors of modern carnivorans and creodonts (Hunt & Tedford, 1993). Given that Carnivora is a group nested well within crown Eutheria, the placement of Cimolestidae is one which impinges strongly on the timescale of placental diversification. If Cimolestidae are indeed closer to Carnivora than to many other Laurasiatherian groups, this would demonstrate that the diversification of the placental mammal lineages occurred at least before the earliest cimolestid material, which is from the Middle Campanian Foremost Formation (approximately 80 Ma), probably significantly earlier. If, however, Cimolestidae are shown to be basal to crown Eutheria, along with the other clades that originate in the Cretaceous, it would be strongly suggestive of a Paleocene diversification event within placental mammals.

Anatomical features consistent with a basal position include the presence of an unusual morphological trait – the prootic canal – found only in Asioryctitheria, Zhelestidae and Cimolestidae among eutherians (Archibald *et al.*, 2001; Ekdale, Archibald & Averianov, 2004). Because both Asioryctitheria are uncontroversially Cretaceous stem placental mammals, it is not unreasonable to suppose that Cimolestidae are also close to the base of placental mammals. Within Placentalia, only *Solenodon* possesses a prootic canal, which appears to be a result of convergence (Wible *et al.*, 2009).

##### (iii) *Ecology*

Cimolestids have in general been considered to have incipiently carnassial teeth (Rana & Wilson, 2003), and as such have been inferred to be faunivorous, if not carnivorous. Indeed, it is the dental similarities that have led to the attribution of this group to the stem of Carnivora. The presence of steep shearing wear marks on the molars of cimolestids (Butler, 1972) illustrates that their teeth were capable of slicing actions, and thus adapted for this diet, but this would be convergent with Carnivora if they are resolved as members of the placental stem.

Cimolestids are relatively primitive in their postcrania, and, like the majority of Cretaceous mammals, their ankle bones suggest a scansorial habit (Szalay & Decker, 1974).

#### (c) *Pantodonta*

##### (i) *Composition*

Pantodonta is composed of several families, the most diverse of which is the Coryphodontidae, which includes 18 species from seven genera. Although the relationships among these families are not well established, there is evidence for the existence of a grouping of exclusively North American pantodonts, the Pantolambdoidea, which includes Cyriacotheriidae, Pantolambdodontidae, Pastoralodontidae, and Titanoideidae (Simons, 1960). In total, Pantodonta includes approximately 35 genera (McKenna & Bell, 1997).

##### (ii) *Proposed relationships*

The pantodonts, for the purposes of this introduction, are considered separately from the Cimolestidae, although they are regarded as a suborder in McKenna & Bell (1997). Superficially, pantodonts are distinct from the majority of the rest of the supposed cimolestids, being large, ground‐dwelling and herbivorous, as opposed to small, scansorial, and carnivorous or insectivorous. Additionally, this classification is a departure from the more traditional interpretations of pantodonts being related to either an assortment of unusual South American ungulates or Paenungulata – the Afrotherian lineage including proboscideans, sirenians and hyracoids. Pantodonts appeared in the Early Paleocene, with a largely global distribution, and survive to the Middle to Late Eocene, whereupon they became extinct approximately 33 Ma.

##### (iii) *Ecology*

Pantodonts include some of the largest terrestrial mammals of the period – the coryphodontids – enormous rhinoceros‐like herbivores which lived from the Arctic to the southern edge of North America (Dawson, 2012), as well as in the Palaeogene of eastern Asia (Ting *et al.*, 2003). They are extremely abundant components of North American Eocene faunas, being common enough to be a stratigraphic indicator for several North American Land Mammal Ages (NALMAs) (Robinson *et al.*, 2004), but have smaller representation throughout the Paleocene.

#### (d) *Creodonta*

##### (i) *Composition*

The status of the 16 genera of Creodonta has long been considered controversial, and the precise composition of the group has changed radically across the history of the literature. Whether the two major groups within Creodonta – Oxyaenidae and Hyaenodontidae – are sister taxa to one another (in other words, whether Creodonta can be considered monophyletic) is not clear (Morlo, Gunnell & Polly, 2009; Zack, 2011). Indeed, their affinity with Carnivora has been suggested to be an artefact of convergent evolution – the superficially similarly shaped carnassial teeth are, developmentally, different teeth (Van Valkenburgh, 1999), suggesting that Carnivora could not have evolved directly from a creodont without significant developmental repatterning.

##### (ii) *Proposed relationships*

Originally described as a group within ‘Insectivora’ (Cope, 1884*c*), Creodonta has been through several iterations, including being related to mesonychians, arctocyonids, carnivorans, palaeoryctids, and even briefly being abandoned as a group completely (for a summary, see Gunnell & Gingerich, 1991). While there has been considerable confusion over what defines a creodont (Polly, 1994; Morlo *et al.*, 2009), the consensus today is that Creodonta is likely a close relative of, although not ancestral to, Carnivora.

##### (iii) *Ecology*

Creodonts were arguably the most specialised placental carnivores, with some members of the group achieving a hypercarnivorous state and modifying their entire molar row to carnassials (Stucky & Hardy, 2007). Members of Creodonta can be considered analogues of several carnivoran clades, with examples of dog‐like, civet‐like, and cat‐like forms (Van Valkenburgh, 1999). The ecological niches exploited by Creodonta are similar enough to Carnivora that hypotheses of competitive exclusion have been invoked to explain the eventual replacement of the former by the latter (Wesley‐Hunt, 2005).

#### (e) *Mesonychia*

##### (i) *Composition*

Mesonychia was traditionally composed of the Asian family Didymoconidae, the semiaquatic Hapalodectidae, and the most speciose member of the clade, Mesonychidae, which includes 19 of the 29 mesonychian genera (Carroll, 1988). Didymoconidae has subsequently been removed from the clade (Meng, Suyin & Schiebout, 1995; Lopatin, 2001), but both hapalodectids and mesonychids remain, with hapalodectids considered more derived than mesonychids. The best known of the mesonychians, *Sinonyx*, *Mesonyx* and *Dissacus*, are all mesonychid mesonychians. The giant mammal *Andrewsarchus* has historically been considered to be a mesonychian or a close relative (Osborn, 1924; Tabuce, Clavel & Antunes, 2011), but competing hypotheses have suggested that *Andrewsarchus* may in fact be a cetancodontomorph artiodactyl (Spaulding, O'Leary & Gatesy, 2009) or an arctocyonid ‘condylarth’ (Van Valen, 1978).

##### (ii) *Proposed relationships*

Mesonychians are an enigmatic group of ‘archaic ungulate’, often considered separate from the ‘condylarths’. Mesonychians were considered, on the basis of shared simplification of the dentition, to be related to whales, but this hypothesis was overturned by the discovery of early whale postcrania, particularly the double pulley astragalus that cemented the position of Cetacea within Artiodactyla, separate from mesonychians (Gingerich *et al.*, 2001; Thewissen *et al.*, 2001). The precise position of the mesonychids with respect to extant clades has been unclear; they have been thought of as stem artiodactyls (Theodor & Foss, 2005) or stem to the clade comprising Artiodactyla and Perissodactyla (Spaulding *et al.*, 2009). Conservatively, they have been grouped with triisodontids and oxyclaenids in the basal placental group Acreodi, and sister to the arctocyonid ‘condylarths’ (Tabuce *et al.*, 2011).

##### (iii) *Ecology*

Apart from a few mesonychians such as *Hapalodectes*, which lack specialised running features of the humerus (O'Leary, 1998), mesonychians have been described as having an ecological niche similar to wolves – that of a cursorially adapted predator (O'Leary & Rose, 1995).

### (3) Paleocene representatives of extant placental clades

#### (a) *Afrotheria (elephants, hyraxes, dugongs, aardvarks, tenrecs, sengis, golden moles)*

The earliest afrotherians known from the fossil record are found in the Middle Paleocene. The species *Ocepeia daouiensis* is known from the Selandian (61.6 to 59.2 Ma) of Morocco, and possesses a mosaic of characters suggesting that it is close to the divergence of Paenungulata and Afroinsectiphilia (Gheerbrant *et al.*, 2014). Its presence in Africa during this time suggests that Afrotheria arose, or at least initially diverged, in Africa, in contrast to some hypotheses which have suggested that at least some afrotherian groups arose in North America (Zack *et al.*, 2005*a*).

#### (b) *Xenarthra (sloths, armadillos, anteaters)*

The location and phylogenetic affinities of the earliest xenarthran is controversial. The earliest member of the crown group that is not disputed is the already highly derived Late Paleocene or Early Eocene armadillo *Utaetus*, which is found from the Casamayoran of Argentina (Ameghino, 1902; Rose, 2006). More controversial is the inclusion of the Asian Paleocene genus *Ernanodon* (Ding, 1987), supposedly part of the suborder Ernanodonta (McKenna & Bell, 1997). This controversy is in part because it occurs on a different continent from other xenarthrans, with the exception of the only other putative xenarthran from Guangdong, *Asiabradypus*. This taxon, however, was considered by Rose *et al.* (2005) to be an animal of unknown affinity, ‘irrelevant to xenarthran origins’. Xenarthrans have historically been grouped together on the basis of simplistic characters such as a lack of teeth. There are few examples of positive characters, with the exception of the additional articulations of the vertebrae which characterise them as xenarthrous, and a relatively highly variable vertebral number (Asher *et al.*, 2011). No clear affinity with typical xenarthran characters has been definitively shown for *Ernanodon*, and Gaudin (1999, p. 30) suggested that the articulations of the vertebrae of *Ernanodon* ‘only vaguely resembles that characteristic of most true xenarthrans’.

#### (c) *Euarchontoglires (rodents, rabbits, pikas, primates, tree shrews, flying lemurs)*

The fossil record of Euarchontoglires in the Paleocene is limited to rodents and primates. The earliest lagomorph (rabbits, hares, pikas) and scandentian (tree shrews) fossils are known from the early to mid Eocene of China and Mongolia (Yongsheng, 1988; Lopatin & Averianov, 2008), while the earliest dermopteran (flying lemur) is known from the Late Eocene of Thailand (Rage *et al.*, 1992).

##### (i) *Rodentia (mice, squirrels, porcupines, guinea pigs, beavers, voles)*

The earliest definitive rodents are known from the Paleocene, with *Tribosphenomys* a close outgroup to Rodentia (Meng & Wyss, 2001) and *Paramys*. Both are known entirely from tooth fragments, but contain crucial synapomorphies that allow identification to their respective positions. Both have the definitive rodent pattern of a single pair of continuously growing incisors with enamel only on the anterior edge.

##### (ii) *Primates (apes, monkeys, lemurs, lorises, tarsiers)*

While there are no definitive crown primates in the Paleocene, there is strong evidence of the presence of plesiadapiforms. These taxa are considered by most to be ancestral to primates, and are arboreal specialists, consistent with the interpretation of many primate features as adaptations for an arboreal lifestyle – for example, grasping hands and a good depth of vision (Rose, 2006). *Purgatorius* is a putative plesiadapiform based on teeth (Clemens, 2004), and recently, tarsal material (Chester *et al.*, 2015). However, its relationships to modern forms has been controversial, with some analyses reconstructing *Purgatorius* outside of Placentalia (Wible *et al.*, 2009). If the Cretaceous Indian genus *Deccanolestes* is, as some have suggested, closely related to purported euarchontans, such as nyctitheres (Hooker, 2001, 2014) or adapisoriculids (Smith, De Bast & Sige, 2010), then *Deccanolestes* would represent a Cretaceous occurrence of a euarchontan. However, while *Deccanolestes* has been shown to be more closely related to adapisoriculids (Goswami *et al.*, 2011), neither group fell within Placentalia. Nyctitheres, by contrast, appear to be more closely related to eulipotyphlans (Manz *et al.*, 2015).

#### (d) *Laurasiatheria*

##### (i) *Carnivora (cats, dogs, bears, otters, badgers, mongooses, hyaenas)*

The earliest stem carnivorans are the genera *Ravenictis* and *Pristinictis*, which are from the earliest Paleocene (Fox & Youzwyshyn, 1994). These earliest forms have relatively unspecialised molars, suggesting a generalised omnivorous diet with only limited specialisation to true carnivory, although *Pristinictis* has been considered a primitive member of Viverravidae. Miacidae and Viverravidae, both relatively derived carnivorans, are both known from the Late Paleocene (Meehan & Wilson, 2002; Sole & Smith, 2013).

Diversification into the major two groups of extant carnivorans – caniforms and feliforms – occurred in the Eocene, but the precise position is dependent on the phylogenetic placement of some enigmatic members of the miacid carnivorans (Tomiya, 2011).

##### (ii) *Pholidota (pangolins)*

Pholidotans are known from the middle Eocene of Europe, being represented by the two genera *Eomanis* and *Eurotamandua*, both from the Messel Pits of Germany (Storch, 1978; Rose *et al.*, 2005). Already relatively derived, a relationship with the Paleocene palaeanodonts has been proposed (Rose, 1999*a*; Gaudin, Emry & Wible, 2009).

##### (iii) *Eulipotyphla (shrews, hedgehogs, moles)*

Eulipotyphla include much of what once was ‘Insectivora’, the basalmost wastebasket taxon of placental mammals from which all others were supposed to have derived (McKenna, 1975). Now recognised as a derived group within Laurasiatheria, if morphologically plesiomorphic, the split between Eulipotyphla and Scrotifera is generally considered to be the basalmost division within Laurasiatheria (Waddell *et al.*, 1999; Nishihara, Hasegawa & Okada, 2006; Zhou *et al.*, 2012), although some earlier molecular analyses support a sister relationship between Eulipotyphla and Chiroptera (Onuma *et al.*, 2000). Combined morphological and molecular analyses consistently have been able to distinguish the ‘true’ insectivores – which comprise moles, shrews, hedgehogs and kin – from the African insectivores – elephant shrews and tenrecs, now known to be members of Afrotheria (Stanhope *et al.*, 1998; Tabuce, Asher & Lehmann, 2008).

If nyctitheres are eulipotyphlans (Manz *et al.*, 2015), the earliest eulipotyphlans in the fossil record are the earliest Paleocene nyctitheres such as *Leptacodon* (Van Valen & Sloan, 1965), with putative but controversial members of the group in the latest Cretaceous (Antunes, Sigogneau‐Russell & Russell, 1986). Other than nyctitheres, the first eulipotyphlans known from the fossil record are from the Late Paleocene, by which time some division into the erinaceids and soricids had taken place (Rose, 1981).

##### (iv) *Chiroptera (bats)*

The first chiropteran fossils are of already relatively derived bats from the Green River Formation of the Early Eocene of Wyoming – *Onychonycteris finneyi* (Simmons *et al.*, 2008) and *Icaronycteris index* (Jepsen, 1966). Morphologically, they were capable of true flight, but unable to echolocate (Simmons *et al.*, 2008). Other dissimilarities with modern bats include a relatively large tail, and, in the case of *Onychonycteris*, the presence of claws on all forelimb digits. No earlier fossil material is attributable to either the crown or stem of bats, making their origins difficult to determine.

##### (v) *Perissodactyla (horses, rhinoceroses, tapirs)*

Of the five main clades of Perissodactyla – Equidae (horses), Tapiridae (tapirs), Rhinocerotidae (rhinoceroses), Brontotheriidae, and Chalicotheriidae – all are known in the earliest Eocene with superficially similar, small, browsing forest‐dwelling forms (Eberle, Rybczynski & Greenwood, 2014). The earliest equid, *Hyracotherium*, underwent a dramatic taxonomic revision in 2002 (Froehlich, 2002), with the separation of the genus into many new (and resurrected) genera. Tapirs and rhinoceroses, which are monophyletic to the exclusion of equids (Froehlich, 1999), are represented in the earliest Eocene by *Heptodon* (Radinsky, 1965) and *Hyrachyus*, respectively. *Heptodon* is known primarily from North America, where the majority of perissodactyl evolution occurred, although there are reports of the genus from China (Chow & Li, 1965). *Hyrachyus* is known from Europe and Asia, but has also been reported from the Caribbean (Domning *et al.*, 1997). Along with these crown members of the perissodactyl families are early members linking the lineages, such as *Mesolambdolophus setoni*, which appears to be close to the base of the tapiromorphs (Holbrook & Lapergola, 2011).

##### (vi) *Artiodactyla (cattle, deer, giraffes, camels, pigs, hippopotamuses, whales)*

Artiodactyla is another extant order whose first members appear at the base of the Eocene (Rose, 1996), with the basal group Dichobunidae, a speciose northern hemisphere group whose best‐known member is the genus *Diacodexis*. Represented by near‐complete specimens (Rose, 1982*b*; Orliac, Benoit & O'Leary, 2012*b*), *Diacodexis* is known from layers immediately above the Paleocene–Eocene boundary (Smith, Smith & Sudre, 1996). It was a cursorial animal capable of high speeds and agile turns, as evidenced by the morphology of the semicircular canals (Orliac *et al.*, 2012*b*) and postcranium (Rose, 1982*b*).

## IV. NEW PHYLOGENETIC ANALYSIS OF CRETACEOUS AND PALAEOGENE PLACENTAL MAMMALS

### (1) Materials

#### (a) *Taxonomic sample*

A broad sample of 177 eutherian taxa was selected in order to evaluate robustly hypotheses of relationships across Placentalia. For both extant orders and extinct groups of unknown affinity, taxa were selected based on several criteria, with particular preference for the most basal members of each lineage. For groups with a limited fossil record, such as all xenarthran groups, dermopterans, and scandentians, and groups where the early relationships and character polarities are not clear, such as in Eulipotyphla, extant taxa were used to supplement fossil material. The reason for preferring fossil taxa over extant forms is that extensive evolutionary change has inevitably occurred within each clade over the last 66 million years. By taking the basalmost and/or earliest members of an order, the chances that key synapomorphies of that group have been obscured through convergence or reversal are far lower.

Terminals were coded at genus rather than species level, to increase character completeness for fossil taxa. Certain genera which have been considered both as separate and synonymous (for example, *Arctocyon* and *Claenodon*, and *Hyracotherium* and *Eohippus*), are treated separately to minimise the potential issue of including poorly supported genera. *Cimolestes* has been suspected to be polyphyletic – Scott (2010, p. 197) states ‘evidence for the monophyly of (Cimolesta) is weak, as is evidence for monophyly of *Cimolestes* Marsh, 1889 itself’ – but is here treated as a single terminal. For the purposes of this study, and lacking any conclusive evidence as regards the monophyly or otherwise of *Cimolestes*, all species assigned to this genus have been considered to represent *Cimolestes*, and are coded into the same terminal to maximise completeness of this important taxon. Completeness and quality of fossil material was also taken into account in taxonomic sampling, with preference for taxa with a higher proportion of codable characters. With the exception of the problematic South American meridiungulate groups of Notoungulata and Litopterna, each group was represented by multiple taxa, to avoid apomorphies being taken as plesiomorphic for a higher clade. In total, 904 specimens and casts were examined in international museum and university collections, supplemented by character data from the published literature, including character state data matrices, scans, and photographs (see online Supporting information, Appendix S1). In total, 177 genera were coded, comprising 130 Palaeogene, 29 Cretaceous, and 18 extant taxa.

#### (b) *Characters*

In total, 680 morphological characters – 48 of which are continuous – were coded for the 177 taxa, resulting in two matrices, one traditionally discrete (Appendix S2), and one with continuous characters treated as such (Appendix S3).

Characters were derived from four major sources – the PhD thesis of Zack (2009), which studied postcranial and dental morphology of largely Paleocene mammals, but excluded cranial characters from the supplied data matrix and included several terminals that were composites of multiple genera; a matrix from Williamson *et al.* (2011) focusing on the Cretaceous–Palaeogene group Cimolestidae, which ultimately descends from the Wible matrix for Cretaceous eutherians (Wible *et al.*, 2007, 2009); a matrix used for establishing the relationships of the Palaeogene ‘ungulate’ mesonychians (Geisler & McKenna, 2007); and a matrix containing several ‘archaic ungulate’ characters, with particular focus on the enigmatic Pleuraspidotheriidae (Ladevèze *et al.*, 2010). Characters were modified such that they were consistently applicable, easily interpreted, and divisions between character states were better and more consistently defined. This resulted in a final list of 680 morphological characters, consisting of 235 dental, 264 cranial, and 181 postcranial characters (Appendix S4).

### (2) Methods

#### (a) *Treatment of continuous characters*

There is much debate over the benefits of using continuous traits in morphological phylogenetic analysis (Rae, 1998; Wiens, 2001; Goloboff, Mattoni & Quinteros, 2006). While more objective than the traditional division of character states in discretized continuous traits, issues arise when determining the relative weighting of a continuous trait. Here, we weight the continuous characters such that the difference between the maximum and minimum values for the trait is equivalent to a single step. Coded values for each terminal taxon were generated through measurement of multiple specimens where possible (Appendix S1) and calculation of the mean value of those measurements. This approach treats the character as effectively equivalent to a binary discrete trait, with the variation in between represented by decimal places within that range. As a result of this treatment of the characters, the steps that take place along the branches of the phylogeny are necessarily on average shorter than when the trait is discretized, which means that the trees are also concomitantly shorter. As a result, it is not possible directly to compare the accuracy of the topology by tree length alone when comparing data sets with and without continuous traits. However, to make sure that the difference in length was exclusively due to the alternate methods of coding particular characters, discrete, ordered, multistate characters were also weighted such that the entire range represented a single step. For binary characters, this requires no weighting, but a three‐state character would be weighted at 0.5 the value of a binary character, since it takes two changes to get from one endpoint to another. We modified the weights of continuous and discretized characters using TNT (Goloboff *et al.*, 2006; Goloboff, Farris & Nixon, 2008).

#### (b) *Constraints on tree topology*

**Figure 2:**
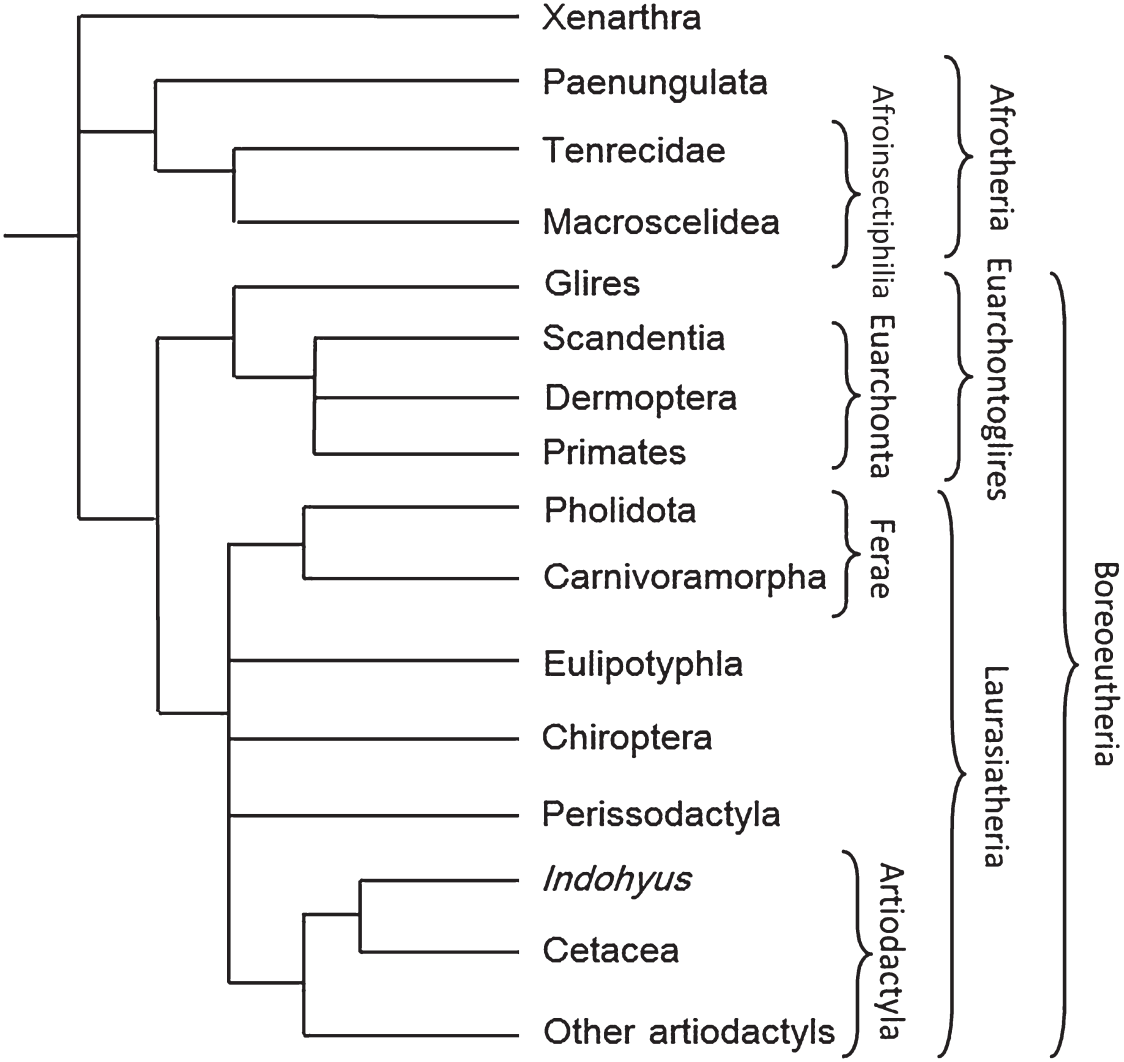
The constraint applied to all analyses, derived from the molecular understanding of the relationships of extant placental mammal groups. In CM and DM analyses, Xenarthra was composed of *Chaetophractus*, *Bradypus* and *Tamandua*; Paenungulata: *Eritherium* and *Procavia*; Tenrecidae: *Potamogale*; Macroscelidea: *Chambius* and *Rhynchocyon*. Glires was composed of *Tribosphenomys*, *Paramys* and *Gomphos*; Scandentia: *Tupaia* and *Ptilocercus*; Dermoptera: *Cynocephalus*; Primates: *Saxonella* *, Cantius*, and *Adapis*. Pholidota was composed of *Eomanis* and *Eurotamandua*, Carnivoramorpha by *Miacis* and *Viverravus*. Eulipotyphla was represented by *Domnina*, *Oreotalpa*, *Blarina*, *Solenodon* and *Echinosorex*. Chiroptera was represented by *Pteropus*, Perissodactyla by *Eohippus* and *Hyracotherium*, Cetacea by *Rodhocetus*, and other artiodactyls by *Gobiohyus*, *Poebrotherium*, *Leptomeryx* and *Elomeryx*. In the CF and DF analyses, additional taxa were, for Xenarthra, *Utaetus*; *Dilambdogale* was added in a polytomy with Macroscelidea and Tenrecidae within *A*froinsectiphilia; for Glires, *Rhombomylus*; for Dermoptera *Elpidophorus*, *Worlandia* and *Plagiomene*; for Primates, *Elphidotarsius*, *Plesiadapis* and *Notharctus*; for Carnivoramorpha, *Didymictis*, *Vulpavus*, *Protictis* and *Uintacyon*; for Eulipotyphla, *Litocherus*, *Uropsilus* and *Centetodon*; for Chiroptera, *Onychonycteris* and *Icaronycteris*; for Perissodactyla, *Heptodon*, *Homogalax*, *Litolophus* and *Lambdotherium*; and for Cetacea, *Pakicetus*. In the CP and DP analyses, *Purgatorius* was further constrained within Primates.

Placentalia is known to display a high level of morphological homoplasy, with adaptive radiations in different groups leading to occupation of similar niches (Madsen *et al.*, 2001), with concomitant morphological similarities. As a result, relationships derived from solely morphological data have often been in conflict with those derived from molecular data, with homoplasy overriding phylogenetic signal at higher phylogenetic levels (Lee & Camens, 2009). As noted above, this issue of homoplasy has long been appreciated, with certain traditional placental groupings (ungulates and insectivores) identified as being particularly suspect (Asher *et al.*, 2008). The most obvious failure of morphological phylogenetic analyses of Placentalia is the lack of support for the major placental ‘superorders’ – Afrotheria, Xenarthra, Euarchontoglires, and Laurasiatheria, each of which are very well supported in most molecular studies (Stanhope *et al.*, 1998; Springer *et al.*, 2003; Bininda‐Emonds *et al.*, 2007; Prasad *et al.*, 2008; dos Reis *et al.*, 2012; Morgan *et al.*, 2013). In order to incorporate the uncontentious aspects of topology for living placentals provided by molecular studies, we constrained the relationships among extant clades with a topological scaffold that is consistent with the vast majority of molecular analyses of placental mammals (Fig. 2). Using molecular constraints can help to correct for morphological homoplasy, and allow the truly synapomorphic morphological features to have a stronger effect.

Rather than constraining taxa, many phylogenetic analyses have used a total‐evidence approach to explore the relationships of groups, incorporating both molecular and morphological data. While this tactic would be possible for these fossil groups, the aim of this study was to elucidate the relationships among the placental mammals of the Paleocene and their affinities with extant orders. Total‐evidence analyses require a substantial proportion of extant taxa. As noted above, we have included earlier and more basal members of extant clades as representatives, as they are less morphologically derived, and are therefore more likely to possess more useful synapomorphies relevant to the diversification of Placentalia, and concomitantly fewer homoplastic traits.

In applying molecular constraints, we allowed for areas of uncertainty such as the topology of the Laurasiatherian orders (Hu, Zhang & Yu, 2012) and the relationships between Boreoeutheria, Atlantogenata and Xenarthra (Murphy *et al.*, 2007; Nishihara, Maruyama & Okada, 2009), discussed further below, by treating them as unresolved polytomies within the scaffold. Two levels of constraint were implemented; one imposed a ‘minimum’ constraint, including a limited subset of exemplar taxa as representatives of the extant orders. For example, while both *Pakicetus* and *Rodhocetus* are undoubtedly closest relatives in this data set, both being stem cetaceans, only *Rodhocetus* was included in the constraint. This approach minimises the degree to which constraints are allowed to affect the data, and is a test that known relationships can still be recovered from the morphological data with a minimal constraint. Exemplar taxa were selected on the basis of both morphological completeness and the level of support for inclusion within a group. For example, within Chiroptera, the extant *Pteropus* was included in the minimum constraint due to high completeness relative to *Onychonycteris* and *Icaronycteris* (both of which are indisputably bats), while within Carnivoramorpha, the genera *Miacis* and *Viverravus* were selected as representatives of Miacidae and Viverravidae respectively. The second approach constrained all taxa which are unequivocally accepted as stem members of the extant orders (Fig. 2) in recent analyses focusing on those groups, in order to ensure that well‐established and evidenced relationships were maintained in these analyses.

Each node that has been constrained is justified on the basis of multiple previous phylogenetic analyses. The ‘four‐clade model’ of placental relationships is now well established (Asher, 2007; Murphy *et al.*, 2007; Wible *et al.*, 2007), but the nature of the relationship between Xenarthra, Afrotheria, and Boreoeutheria, the well‐accepted grouping of Laurasiatheria and Euarchontoglires (Delsuc *et al.*, 2002; Asher & Helgen, 2010) is unclear (Hallstrom *et al.*, 2007; Murphy *et al.*, 2007; Morgan *et al.*, 2013; Teeling & Hedges, 2013). As a result, the scaffold is constrained to allow any topology between these three groups. Within Afrotheria, the basal separation of ‘ungulate‐grade’ from ‘insectivore‐grade’ organisms is well established (Tabuce *et al.*, 2008; Asher & Helgen, 2010), and therefore was also constrained. The division between Glires and Euarchonta is supported by a number of analyses, but the relationships within the orders of Euarchonta differ among them (Waddell, Kishino & Ota, 2001; Nie *et al.*, 2008). As a result, Primates, Dermoptera, and Scandentia, all certainly monophyletic, were allowed to vary in their interrelationships within Euarchonta. Within Laurasiatheria, there is no clear consensus for the relationships of the orders (Hallstrom *et al.*, 2011; Hu *et al.*, 2012), except that Carnivora and Pholidota are likely to be sister taxa (Nishihara *et al.*, 2006; Zhou *et al.*, 2012). Raoellid artiodactyls (including *Indohyus*) are considered to be closer relatives of cetaceans than other artiodactyls in this analysis (Bajpai, Thewissen & Sahni, 2009), and as a result, the topologies within Artiodactyla were also constrained to reflect this basal division.

Further, there remains particular doubt as to the status of the enigmatic genus *Purgatorius*. This early Paleocene genus has been allied by many to the plesiadapiforms (Clemens, 2004; Fox & Scott, 2011), with the implication that it represents an early stem primate. Alternative topologies have placed *Purgatorius* on the stem of Placentalia, due to the conservativeness of its morphology (Wible *et al.*, 2009; Rook & Hunter, 2014), although counterarguments suggest that this more basal position has resulted from inadequate sampling of plesiadapiforms and early Primates (Chester *et al.*, 2015), which could also potentially affect its positioning in this analysis. In order to accommodate these alternative hypotheses, which are both substantially supported on the basis of tarsal and dental similarities, respectively, *Purgatorius* was constrained along with Primates and their kin in a further analysis, and left unconstrained in the others.

In implementing these constraints, all taxa involved in the constraint were set as ‘non‐floaters’ in TNT, while all others were set as ‘floaters’, meaning that they are able to invade an otherwise constrained topology. *Peramus*, *Deltatheridium*, and *Bobolestes* were set as sequential outgroup taxa in the constraint, as all are unambiguous stem eutherians (McKenna & Bell, 1997), in order to ensure that trees were rooted appropriately.

In total, we used three different constraints with two types of data, as well as running an unconstrained analysis with both data matrices, resulting in eight separate sets of most parsimonious trees (MPTs). For brevity, we refer to these eight analyses using the following abbreviations: CU and DU represent the continuous and discretised unconstrained analyses. CM and DM the minimum constraints; CF and DF the full constraints in which all unambiguously placed fossil taxa are included, and CP and DP the constraints equivalent to CF and DF, but with *Purgatorius* constrained with the Primates.

#### (c) *Phylogenetic analysis*

Maximum parsimony phylogenetic analysis was carried out in the freeware program TNT (Goloboff *et al.*, 2008), using the New Technology Search algorithms. The consensus was stabilized twice with factor 75, employing random and exclusive sectorial searches, drift (rejection factor 50) and tree fusing, dumping fused trees for computational ease due to the size of the data set. This was followed by a round of traditional tree bisection and reconnection (TBR) searching, using the MPTs from the New Technology Search as starting trees for the TBR analyses, following Mannion *et al.* (2013). Analyses each took approximately 350–500 h of computing time. Multistate characters were treated as ordered where meristic or where they represented a morphological sequence in which one or more states are discrete intermediates between end‐member states (Wilkinson, 1992). For example, character 325 describes the position of the palatine foramen, and has the ordered states ‘within palatine’, ‘between palatine and maxilla’ and ‘within maxilla’ (see Appendix S4). Due to the precision of continuous and weighted discrete analyses of multiple decimal places, near‐optimal trees which were less than a step longer than the MPTs were also recovered.

#### (d) *Templeton's tests*

Templeton's test (Templeton, 1983) allows alternative, suboptimal topologies to be compared in order to ascertain whether the additional length is significantly longer than the optimal topology, and therefore can be rejected as unsupported by the data. Where two competing hypotheses for the phylogenetic placement of a taxon exist, Templeton's tests are therefore a useful way to determine whether a data set supports one hypothesis strongly over another. Pairwise Templeton's tests were conducted on each set of MPTs for both discrete and continuous data sets using Microsoft Excel.

#### (e) *Bremer support*

Relative Bremer supports were calculated by searching for suboptimal trees at increasing levels of suboptimality until the storage limit of 99999 trees was reached in TNT, calculating relative support, and subsequently pruning out those taxa that were causing local reduction in support due to their instability. Relative Bremer supports measure the degree to which topologies supporting a clade outnumber those invalidating a clade within a set of trees, and give a corresponding value between −100 and 100, where −100 represents topologies that are never supported, and a score of 100 clades that are always present. For example, a score of 50 would indicate that the number of trees that contradicted the clade was half that of the number that supported the clade. Values of 0 or below result in the node being collapsed, as they are contradicted by a majority of trees. Relative Bremer support holds the advantage over absolute Bremer support of taking into account contradictory and favourable evidence for a clade, rather than just favourable evidence (Goloboff & Farris, 2001). Additionally, because they vary within the same scale, measuring a ratio, results can be directly comparable across trees. As a result, it has been argued that relative Bremer support is a superior metric of node support to absolute Bremer support (Goloboff & Farris, 2001).

## V. RESULTS

### (1) Phylogenetic topology

The MPTs resulting from the six constrained analyses were generally consistent with each other. For clarity, only the results from three analyses will be discussed in detail – the discrete, unconstrained tree (DU), and both continuous and discrete trees, with the full constraint applied (CF and DF). Details of the differences between these trees and those derived from alternative constraints (the ‘minimum’ constraint and full constraint incorporating *Purgatorius*) may be found in Appendix S5. Numbers of MPTs, number of suboptimal trees within a single step of the MPTs, and tree metrics are summarised in Table 1.

**Table 1:** Numbers of most parsimonious trees (MPTs), their lengths, the number of suboptimal trees within a single step, and consistency (CI) and retention (RI) indices. Across all analyses, homoplasy is extremely high. Lengths of trees that are not whole numbers are due to the presence and weighting of continuous characters or discretised and reweighted continuous characters

MATRIX:	CU	CM	CF	CP	DU	DM	DF	DP
No MPTs	5	4	2	8	79	60	480	10
Length	7820.16876	8009.03713	8017.90619	8059.26802	8330.75	8471.9	8521.8	8528.23
No Suboptimal	4163	39516	6672	20448	8506	3950	8884	1054
CI	0.111	0.108	0.108	0.108	0.111	0.109	0.108	0.108
RI	0.448	0.431	0.432	0.429	0.446	0.434	0.431	0.430

#### (a) *Unconstrained analyses*

**Figure 3:**
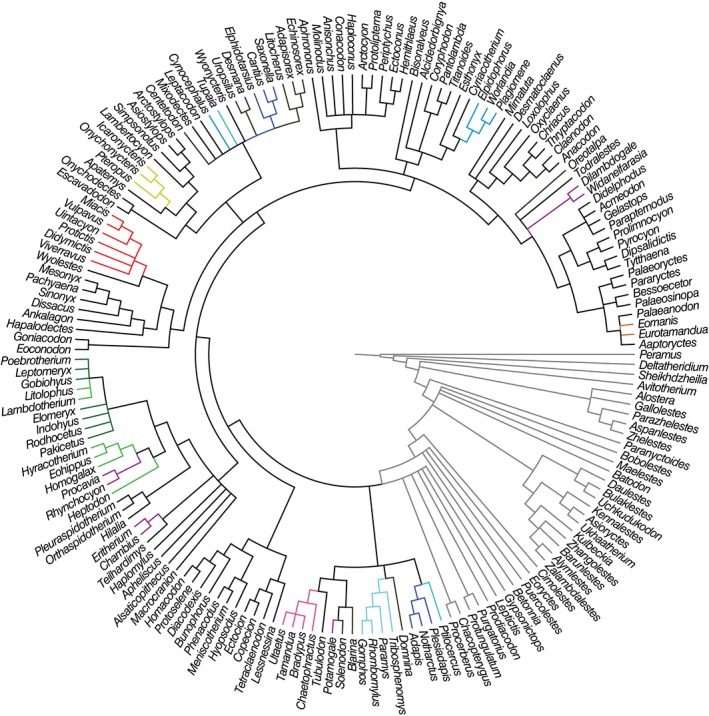
Strict consensus of all discrete, unconstrained (DU) trees within one step of the most parsimonious trees. Colours represent members of extant orders as follows: pink, Xenarthra; purple, Afrotheria; sky blue, Glires; light blue, Scandentia; mid‐blue, Dermoptera; royal blue, primates; brown, Eulipotyphla; dark green, Artiodactyla; light green, Perissodactyla; yellow, Chiroptera; orange, Pholidota; red, Carnivora.

The topology of the unconstrained analysis (Fig. 3, see online Fig. S1) contained many of the groupings that have been generally supported by previous morphological analyses, and failed to recover Eulipotyphla as a monophyletic group to the exclusion of other laurasiatherians. Afrotheria was recovered as polyphyletic and Chiroptera allied with a reduced Euarchontoglires. Aspects of the topology such as these demonstrate the need for constraining relationships among extant clades to those that are well supported by both molecular and phenotypic data sets, such as the four superorders. Results were consistent between discretised and continuous characters.

#### (b) *Fully constrained analyses*

**Figure 4:**
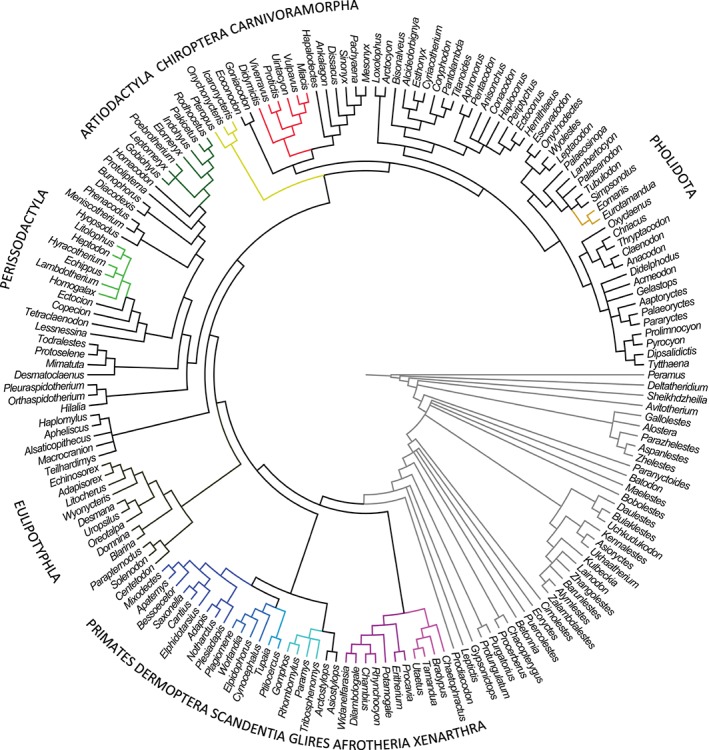
Strict consensus of trees derived from the discrete data set with the full constraints applied (DF analysis). Colours are as in Fig. 3.

When full constraints were implemented as described in Section IV.2*b*, the precise topology of extant clades varied where there was uncertainty, for example in the relationships among the laurasiatherian orders (Fig. 4, see online Figs S2–S6). Nonetheless, topological relationships of the clades of interest were generally consistent among all constrained analyses. The retention and consistency indices were similar in the constrained and unconstrained analyses (Table 1), indicating that the level of homoplasy in the unconstrained tree was almost as high as when relationships were constrained.

Below, we discuss relationships of clades of interest, and the synapomorphies that support them. Full lists of synapomorphies for major recovered clades are found in Appendix S6.

#### (c) *Stem placentals*

In all analyses, *Protungulatum* was most parsimoniously reconstructed as a non‐placental eutherian, contrary to previous suggestions that it represented the earliest crown placental, or that it was an arctocyonid ‘condylarth’. *Purgatorius* was found consistently as sister taxon to *Protungulatum*, as in Wible *et al.* (2007), with both taxa immediately stemward of a paraphyletic Leptictida. Zhelestidae was recovered in a basal stem‐placental position as opposed to being a stem member of an ‘ungulate’ clade within the crown. The monophyly of Zhelestidae was supported by the presence of a metaconid on p4 (Character 131), the separation of a relatively small paraconid from the metaconid (19, 20, 214), a hypoconulid close to the entoconid (236) and a mandibular ramus that deepens below the molars (263). Zalambdalestidae, too, was recovered as a monophyletic group of stem placentals, and was supported by several unambiguous synapomorphies, including the development of an enlarged, procumbent lower first incisor with an extensive root (72, 74, 76), a more anterior position of the posteriormost mental foramen (261), the lack of an ectoflexus on any upper molar (160), an uneven distribution of enamel on the incisors (256), and the presence of a separate metaconid on the fourth premolar (131).

#### (d) *Cimolestidae*

Cimolestids were found to be diphyletic in all analyses; one group, consisting of *Cimolestes*, *Procerberus*, *Chacopterygus*, *Betonnia* and *Puercolestes*, was consistently placed in a relatively basal position on the eutherian stem. However, the cimolestid *Gelastops* was reconstructed as part of the broadly carnivorous radiation of mammals including palaeoryctidans, creodonts, and Ferae, falling out specifically with the palaeoryctidan *Acmeodon* and the mesonychid *Wyolestes*. The inconsistency in the composition of this group means that there are few synapomorphies that are supported across all analyses. However, a premaxilla that extends beyond the canine (293), sharp, gracile molar cusps (138), and a small ectopterygoid process (387) are consistently reconstructed where this broadly carnivorous grouping is recovered as monophyletic.

#### (e) *Leptictida*

The three leptictidans were not recovered as monophyletic in any analysis, but *Gypsonictops* and *Leptictis* were found to be sister taxa in all analyses, with synapomorphies including prominent premolar conules (112), a developed cristid obliqua (230), and the presence of a hypoconule on upper molars (206). In the CF and DF analyses, *Prodiacodon* was found to be the sister taxon to crown Placentalia, with *Gypsonictops* and *Leptictis* the next closest relatives, rendering Leptictida paraphyletic with respect to Placentalia.

#### (f) *The placental root and higher‐level relationships*

Molecular and morphological analyses have been equivocal in support for the three prevailing hypotheses for the placental root topology (e.g. Churakov *et al.*, 2009). Here, in all constrained analyses, a split between Atlantogenata and Boreoeutheria was favoured as the root of placental mammals, rather than either Xenarthra or Afrotheria being most basal among placental superorders, as has previously been hypothesised (Gaudin *et al.*, 1996; Waddell *et al.*, 2001). This result is consistent with many recent genetic and genomic analyses of placental mammals (Hallstrom *et al.*, 2007; Murphy *et al.*, 2007; Kuntner, May‐Collado & Agnarsson, 2011). Morphological synapomorphies for Atlantogenata were inconsistent across different analyses, due to the shifting relationships of other taxa. Those which remain across the majority of analyses were dental – typically related to the simplification of the molars – which poses problems for identifying these traits in edentulous taxa such as the majority of Xenarthra. Commonly reconstructed synapomorphies of Atlantogenata included loss of the pre‐ and postcingula (109, 110), as well as a vertical lingual face of the protocone (191), and the presence of a hypoconid on the second lower molar (232). In the DF and CF analyses, 26 and 25 synapomorphies, respectively, supported Atlantogenata, of which 21 were common to both (Appendix S6). Examples of these are postcranial characters including an increase in the number of thoracic vertebrae (524), a rounded rather than ovoid radial head (560), and a shortened astragalar neck (630), as well as many additional losses in cheek tooth complexity. However, no taxon was consistently resolved on the stem of either Atlantogenata or Boreoeutheria.

Although Laurasiatheria and Euarchontoglires were each constrained to form monophyletic clades in all analyses, they were nonetheless supported by a number of unambiguous synapomorphies. Character transitions which consistently occurred at the base of Laurasiatheria include the movement of the foramen ovale to a medial position relative to the glenoid fossa (393), the opening of the cavum epiptericum (419), the loss of the hypotympanic sinus (452), and, where present, more distal re‐entrant grooves on the molars (257). Eulipotyphla was supported as the most basal extant order within Laurasiatheria in all constrained analyses, but the relationships among other laurasiatherian orders were more variable. With the exception of the minimum constraints analyses, the next most basal division within Laurasiatheria was between Euungulata (Artiodactyla and Perissodactyla) and a Chiroptera–Ferae (Carnivora and Pholidota) clade. Characters supporting Euungulata include a flattened ulnar facet on the radial head (560), the lack of a paraconid on the lower molars (213) or a preparacrista on the upper molars (164), and an elongate calcaneal tubercle (656), while the Chiroptera–Ferae clade was supported by a loss of a postpalatine torus (328), a laterally exposed mastoid region (508), three sacral vertebrae (527), and an inferior petrosal sinus that was housed between the petrosal, basisphenoid and the basicranium (476).

Euarchontoglires was supported unambiguously by an extended ectopterygoid process of the alisphenoid (386), an anteriorly expanded tegmen tympani (447), a small and shallow stapedius fossa (463), and a reduction to three sacral vertebrae from four (527). The most common division within Euarchonta is a Scandentia–Dermoptera clade to the exclusion of Primates; this is supported by five consistent and unambiguous synapomorphies. These are a reduction in the number of lower incisors (57), a loss of contact between the jugal and lacrimal (339), the absence of a sagittal crest (372), the presence of the interparietal (373), and a fused scaphoid and lunate (575).

#### (g) *‘Condylarths’*

All major ‘archaic ungulate’ groups were resolved within Laurasiatheria, with a division between broadly herbivorous taxa on the one hand and carnivorous–insectivorous ones on the other. Phenacodontidae was not recovered as monophyletic. One group (*Tetraclaenodon*, *Copecion* and *Ectocion*) was consistently resolved as paraphyletic with respect to Perissodactyla; this was one of the best‐supported relationships, even being recovered in unconstrained analyses. However, *Phenacodus* and *Meniscotherium* were found among hyopsodontids in all analyses. With the exception of the presence of a mesostyle (148), there was no single synapomorphy that was unambiguously associated with a node subtending perissodactyls and the three phenacodontids when all analyses were considered. Nonetheless, several character states, such as a strong metalophid (221), highly molarised premolars (118, 119) and the loss of upper molar conular cristae (184) are synapomorphies in a majority of analyses. Pleuraspidotheriidae was also consistently included towards the base of an ungulate group including Perissodactyla, and, sometimes, Artiodactyla.

Contrary to suggestions that Apheliscidae is related to Macroscelidea, apheliscids were here recovered in a basal position within Laurasiatheria, sister to Scrotifera (the clade comprising all laurasiatherian orders except Eulipotyphla) in all analyses except CM and DP. Hyopsodontids are placed, in all analyses except CM and DM, as the sister taxon to Artiodactyla. Periptychidae and Pantodonta are consistently found to be sister taxa, more closely related to Ferae and Chiroptera than to other Laurasiatherian orders. Arctocyonidae was polyphyletic in the DF and CF analyses, with *Arctocyon* and *Loxolophus* sister to the Pantodonta–Periptychidae clade, *Goniacodon* and *Eoconodon* sister to a Carnivora–Mesonychia clade, and the remaining genera allied with creodonts and palaeoryctidans.

#### (h) *Other Paleocene taxa*

The close relationship between Creodonta and Carnivora was consistently supported, with other pseudocarnivorous genera such as *Gelastops*, *Acmeodon*, *Wyolestes* and *Didelphodus*, as well as Palaeoryctidae, also placed as close relatives to this grouping. Moreover, palaeanodonts are found to be sister taxa to Pholidota (represented here by *Eomanis* and *Eurotamandua*), supported by a strong teres tubercle (544), a central process of the radial head (559), a shallow olecranon fossa (573), and no iliopubic eminence (587).

The enigmatic South American meridiungulates are represented in this study by the henricosbornid notoungulate *Simpsonotus* and the early litoptern *Protolipterna*. *Protolipterna* was resolved alongside archaic dichobunid artiodactyls in most analyses. *Simpsonotus*, however, had a less consistent position, being found next to Palaeanodonta in DF, CM and CP, but on the atlantogenatan stem with Arctostylopidae in DP, on the chiropteran stem with Arctostylopidae in CF, and close to Artiodactyla in DM. As the sampling in this study does not adequately capture the diversity of meridiungulates, which include at least five distinct and unusual clades, further work focusing on this group is certainly required to clarify their affinities. The relationships presented here provide a starting point from which a more detailed analysis of this group can proceed, by including these potential close relatives of the South American ungulates. Neither *Protolipterna* nor *Simpsonotus* were resolved as close relatives of Perissodactyla, contrary to recent evidence from protein sequences (Welker *et al.*, 2015).

The relationships of Arctostylopidae are extremely poorly understood (Zack, 2004), but this group has been thought to be related to Glires, Notoungulata, or Artiodactyla (Cifelli, Schaff & McKenna, 1989; McKenna & Bell, 1997). Affiliation with Glires, supported by the DF analysis, was supported by mandibular and postcranial characters such as a single mental foramen (259), a space between m3 and the coronoid process (264), and a rotated sustentacular facet of the astragalus (626). A relationship with Notoungulata was supported in the CF and DP analyses by reduction of the metacone and protocone (9, 12), as well as smaller canines (83, 86), reduced protocristid (223), and a more even‐sized tooth row (137). A more focused study of this enigmatic and rare family is required to resolve the character conflict between this hypothesis and that implied by an Arctostylopidae–Glires clade.

### (2) Templeton's tests

Templeton's tests were used to compare all pairwise combinations of MPTs – a total of 42 comparisons. The lengths of all constrained topologies were found to be significantly longer than those of unconstrained trees for a given data set (Table 2), with the latter bearing no relationship to the known topologies of placental mammal phylogenies derived from molecular data. This result suggests that morphological data cannot of itself accurately reconstruct placental phylogeny without application of constraints, due to substantial differences between topologies resulting from constrained and unconstrained analyses. Nonetheless, the constraints that were applied are based upon well‐established relationships that are consistently retrieved from both molecular and combined morphological–molecular analyses, and so are justified in this context.

**Table 2:** Results of Templeton's tests, comparing each set of topologies under both discrete and continuous data sets. Of all constrained topologies, the only comparison that was considered to be significantly different is that between the discrete (DM) and continuous (CM) minimum constraints under the discrete data set

Data set	Shorter topology	Longer topology	*W*	*n*	*z*	*P* value (two‐tailed)
Continuous	CU	CF	22398	406	4.73	**<0.0001**
Continuous	CU	CP	20772	414	4.26	**<0.0001**
Continuous	CU	CM	23174	421	4.64	**<0.0001**
Continuous	CU	DF	20294	423	4.03	** 0.0001**
Continuous	CU	DP	24412	429	4.75	**<0.0001**
Continuous	CU	DM	19465	407	4.1	**<0.0001**
Continuous	CM	CF	1071	327	0.31	0.7566
Continuous	CM	CP	1451	345	0.39	0.6965
Continuous	CM	DF	342	353	0.09	0.9283
Continuous	CM	DP	2856	353	0.74	0.4593
Continuous	CM	DM	2733	327	0.8	0.4237
Continuous	CF	CP	1184	294	0.41	0.6816
Continuous	CF	DF	1838	313	0.57	0.5687
Continuous	CF	DP	2207	305	0.72	0.4715
Continuous	CF	DM	2009	329	0.58	0.5619
Continuous	CP	DF	370	201	0.22	0.8259
Continuous	CP	DP	1842	305	0.6	0.5485
Continuous	CP	DM	999	358	0.25	0.8026
Continuous	DF	DM	2389	360	0.6	0.5485
Continuous	DP	DM	5259	380	1.23	0.2187
Continuous	DF	DP	161	301	0.05	0.9601
Discrete	DU	DF	35176	410	7.33	**<0.0001**
Discrete	DU	DP	37655	414	7.73	**<0.0001**
Discrete	DU	DM	34198	425	6.75	**<0.0001**
Discrete	DU	CF	40483	421	8.1	**<0.0001**
Discrete	DU	CP	38274	420	7.69	**<0.0001**
Discrete	DU	CM	41344	430	8.02	**<0.0001**
Discrete	DM	DF	4359	348	1.16	0.246
Discrete	DM	DP	5911	370	1.44	0.1499
Discrete	DM	CF	5980	324	1.77	0.0767
Discrete	DM	CP	5623	348	1.5	0.1336
Discrete	DM	CM	7540	321	2.27	** 0.0232**
Discrete	DF	DP	1049	296	0.36	0.7188
Discrete	DF	CF	439	306	0.14	0.8887
Discrete	DF	CP	1751	194	1.12	0.2627
Discrete	DF	CM	3334	339	0.92	0.3576
Discrete	DP	CF	1397	302	0.46	0.6455
Discrete	DP	CP	2756	302	0.91	0.3628
Discrete	DP	CM	1865	346	0.5	0.6171
Discrete	CF	CP	1613	284	0.58	0.5619
Discrete	CF	CM	2282	316	0.7	0.4839
Discrete	CM	CP	1274	334	0.36	0.7188

*p*‐values significant at an alpha level of 0.05 are in bold.

Among the constrained topologies, no significant differences were found in either discrete or continuous character optimisations using the same constraints (Table 2), and for each data set, no particular constraint resulted in significantly longer trees than any other constraint. When different constraints and data types were compared in concert, significant differences were found between DM and CM under the discrete data set. In this case, the continuous topologies were significantly longer due to the trees derived from the discrete data set better fitting the discretised data. All other differences in length between combinations of data set and constraint were not significant.

### (3) Relative Bremer support

Relative Bremer support was low for several nodes across all trees (see online Figs S7–S12). The lack of support is in large part due to a few very unstable taxa, as confirmed by application of the ‘Pruned trees’ option in TNT, which identifies taxa that, when removed, result in the resolution of polytomies (Table 3). When relative Bremer supports were calculated after pruning these unstable taxa from the suboptimal topologies, the level of support increased markedly. In the CF analysis, many higher‐level relationships were strongly supported, being found in all suboptimal topologies to the storage limit of 99999 trees. Well‐supported relationships included the placement of all ‘condylarths’ within Laurasiatheria, the monophyly of Euungulata, the closest relatives of Perissodactyla being phenacodontids, and the affinity of Hyopsodontidae with Artiodactyla. Additionally, the placement of triisodontids with Arctocyonidae as a sister clade to Carnivora and Creodonta was very strongly supported. When *Eoryctes* was excluded, Atlantogenata was supported 100% of the time, as was the paraphyletic relationship of Leptictida with respect to crown Placentalia.

**Table 3:** Unstable taxa pruned from each topology for the calculation of representatives

Level of Constraint	Continuous	Discrete
Unconstrained	n/a	*Lainodon*, *Pentacodon*
Minimum	*Hilalia*, *Lainodon*, *Oxyclaenus*	*Lainodon*, *Oreotalpa*
Full	*Centetodon*	*Molinodus*
*Purgatorius*	*Prodiacodon*	*Eoryctes*, *Molinodus*, *Lainodon*

In the CM analysis, node support was in general weaker, although monophyly of many Paleocene clades was conserved. *Ectocion*, *Copecion* and *Tetraclaenodon* were still strongly supported as sequential closest relatives to perissodactyls. After excluding unstable taxa (*Lainodon*, *Oxyclaenus* and *Hilalia*) from the CM analysis, Atlantogenata was well supported, but laurasiatherian clades generally received poorer support than in the other analyses (see online Fig. S11).

In the DF analysis, support values were generally higher than other analyses, even before pruning unstable taxa, with relatively good support for a Hyopsodontidae–Artiodactyla relationship, and very high support for an arctocyonid–creodont–palaeanodontan clade. Atlantogenata, the laurasiatherian affinity for ‘condylarths’, the relationship between Perissodactyla and some phenacodontids, and a Triisodontidae‐Carnivora relationship were also notably all supported. The DM and DP analyses were very stable once unstable taxa (*Lainodon* and *Oreotalpa* in the former; *Eoryctes*, *Molinodus* and *Lainodon* in the latter) had been excluded from the strict consensus tree, with strong support for many higher‐level relationships within the phylogeny.

Supports for nodes across crown Placentalia were weaker when *Purgatorius* was constrained as a primate. In all analyses, the weakest area of support was within Ferae, where relative Bremer indicated almost equivocal support for the presence or absence of any given clade. The best‐supported topologies across the entire eutherian tree were found in the DF analysis (Fig. 4, see online Fig. S7).

## VI. DISCUSSION

### (1) Comparison with previous studies

This study represents a significant methodological improvement over all previous analyses that have sought to understand the affinities of enigmatic Paleocene groups. The only study to approach similar numbers of Palaeogene taxa (130 in this analysis) was the PhD thesis of Zack (2009). However, several of the terminals in that analysis are composites of multiple genera, which therefore may not represent the character distributions of any actual organism, and may result in topologies supported by none of the data from each constituent taxon (Malia, Lipscomb & Allard, 2003). Further, the Zack (2009) analyses presented trees derived primarily from dental and postcranial material. While cranial material, a rich source of data, was included in a single analysis, this was unconstrained to a backbone of known relationships, and as a result contains highly improbable topologies that contradict the body of scientific work to date. Excluding cranial data in all other analyses effectively ignores a rich source of phylogenetic information which may be more reliable than dental data in phylogenetic analyses (Sansom, 2014) due to atomisation of characters and strong functional correlations among occluding teeth. Otherwise, all other studies of mammal phylogenetics have included few, if any, Paleocene taxa, and those that do have focussed primarily on ordinal‐ or family‐level clades (e.g. Missiaen *et al.*, 2012; Chester & Bloch, 2013). While adequate for understanding relationships within groups, these smaller analyses are unable to test all competing hypotheses of placental interrelationships. For instance, by studying apheliscid and louisinid ‘condylarths’, but only including single members of Macroscelidea, Amphelimuridae, Adapisoriculidae, and outgroups (as in Hooker & Russell, 2012), it is possible to study the interrelationships of apheliscids and louisinids, but not possible to test alternative hypotheses of the relationships robustly between these taxa and the rest of the placental tree. The inclusion of a combination of living and fossil members of Placentalia in the present analysis advances our understanding of placental mammal origins, providing additional data, new resolution, and novel perspectives on the long‐debated affinities of several enigmatic clades.

### (2) Resolving placental relationships

Atlantogenata was strongly supported over Exafroplacentalia or Epitheria for the first time in an exclusively morphological analysis – albeit one incorporating constraints in other portions of the tree. While Atlantogenata has been supported by a wide range of molecular studies (e.g. Hallstrom & Janke, 2008; Prasad *et al.*, 2008; Song *et al.*, 2012; Morgan *et al.*, 2013), analyses including morphology have tended to favour a xenarthran root (Gaudin *et al.*, 1996; O'Leary *et al.*, 2013). The concordance between topologies derived from previous molecular studies and this morphological study suggests that a solution to the conflict between data sources may be possible, despite the degree of convergence that is clearly present in placental mammal morphology. Although molecular constraints were implemented in this tree, the topology at the root of Placentalia was left unconstrained, and Atlantogenata was recovered in all analyses. That there are dental synapomorphies at the node subtending Atlantogenata despite the extremely simplified dentition of xenarthrans can be explained by noting that the majority of dental characters that support Atlantogenata are concerned with structures that are lost in both Afrotheria and Xenarthra.

We found that a broad division can be drawn within Scrotifera between a loosely ‘ungulatomorph’ clade, including Artiodactyla, Perissodactyla, Hyopsodontidae, Phenacodontidae and Pleuraspidotheriidae, and a remaining group of more insectivorous, omnivorous, and carnivorous taxa, including Chiroptera, Mesonychia, Palaeanodonta, Pholidota, Carnivora, Creodonta, Palaeoryctidae and Arctocyonidae, associated with Periptycidae and Pantodonta.

The polyphyly of ‘Condylarthra’ and its status as a wastebasket taxon are supported by a large number of morphological features. Several enigmatic Paleocene groups within this wastebasket, including Periptychidae, Pantodonta and Mesonychia, as well as, in a majority of analyses, Arctocyonidae, are resolved as monophyletic sister taxa to extant clades. Others, such as Leptictida and Cimolestidae are recovered as paraphyletic with respect to extant clades. The polyphyly of Hyopsodontidae, which has long been suspected (Cifelli, 1983; Archibald, 1998; Zack *et al.*, 2005*a*), is also supported here, with Pleuraspidotheriidae, Apheliscidae and the ‘true’ hyopsodontids *Hyopsodus* and allies found to be entirely separate lineages (Fig. 4).

Although Phenacodontidae is here returned as diphyletic, the consistent support for the presence of phenacodontids as closest relatives of Perissodactyla is in agreement with the majority of the literature. The original definition of ‘Condylarthra’ was as a subgroup of Perissodactyla, and was largely composed of phenacodontids (Cope, 1884*a*; Rose, 2006); phenacodontids and perissodactyls have been considered close relatives since (Radinsky, 1966; Thewissen, 1990; Zack, 2009; Ladevèze *et al.*, 2010), although sometimes Phenacodontidae has been identified as the sister group of Altungulata (Kondrashov & Lucas, 2012), a problematic grouping of perissodactyls and several afrotherian ‘ungulates’, which is in conflict with molecular topologies.

The placement of creodonts closer to pangolins than to carnivorans has not been recovered in previous phylogenetic analyses. While Creodonta has been suggested to be paraphyletic relative to Carnivora (Flynn & Wesley‐Hunt, 2005), the distinctive carnivoran carnassial teeth are composed of the upper fourth premolar and the lower first molar, rather than solely molars as seen in Creodonta (Colbert, 1933; Colbert & Morales, 1991; Goswami, 2010; Ungar, 2010), suggesting a possible convergent acquisition of this phenotype. Additionally, monophyly of Creodonta is not always recovered (Polly, 1996; Morlo *et al.*, 2009; Sole *et al.*, 2009). A position within Ferae – the clade uniting Carnivora and Pholidota – is accepted (MacIntyre, 1966; Smith & Smith, 2001), but the relative positions of Pholidota, Carnivora, and Creodonta have been unclear.

While many relationships presented in this study are consistent across analyses, several remain poorly supported. Although *a posteriori* pruning of unstable taxa removes some of the uncertainty in relationships, there are several aspects of the tree that still remain to be clarified with additional data, including the position of apheliscine condylarths and the topology of the enigmatic groups here resolved on the stem of Ferae. Indeed, evidence is accruing that the diversification of the laurasiatherian orders occurred extremely rapidly, (Hallstrom &Janke, 2008; Zhou *et al.*, 2012) such that incomplete lineage sorting has been invoked as an explanation for lack of resolution in early placental relationships (Hallstrom & Janke, 2010).

Clearly, there is little consensus for the majority of the relationships of Paleocene placental mammals, and many of the relationships supported herein will continue to be debated. Many of the results from this analysis actually resurrect previous hypotheses from recent and more distant studies. The nature of a wastebasket taxon, particularly one with such a long history as ‘Condylarthra’, is that many hypotheses of internal and external relationships have been and are being put forward, based upon different lines of evidence. This study, in presenting relationships supported by a broad skeleto‐dental matrix combined with molecularly derived constraints, supports topologies which are largely consistent with at least some of the literature and provides new resolution to the placental tree.

With the inclusion of Paleocene taxa into a broad phylogenetic tree for eutherian mammals, we have a window into the time during which the placental diversification was occurring, and are able to break the methodological constraints of using only extant data to peer back at events whose effects on the genome, have, over time, been overwritten and obscured. Use of molecular constraints which can overcome problems of homoplasy in morphological data help to reveal past patterns (Davalos *et al.*, 2014), meaning that integration of palaeontological and neontological data is essential to answer questions of ancient relationships. This analysis represents an important step in untangling the relationships of these extinct clades, and understanding the evolutionary and ecological context of the radiation of placental mammals. Ongoing work is focused on robustly dating the topologies produced in this phylogenetic analysis, and investigating the rates of morphological character evolution and changes in morphological disparity over the K/Pg boundary in order to ascertain whether the end‐Cretaceous mass extinction had a discernible effect on the macroevolutionary patterns within eutherian mammals.

### (3) Implications for the timing of placental origins

As noted in previous studies including *Protungulatum* (Wible *et al.*, 2007; Archibald *et al.*, 2011; O'Leary *et al.*, 2013), the phylogenetic position of that taxon is critical to the interpretation of the oldest known members of crown Placentalia. *Protungulatum* (and *Purgatorius*) were most parsimoniously resolved on the stem of Placentalia, except where *Purgatorius* was constrained. However, Templeton's tests do not reject alternative phylogenetic positions for either *Protungulatum* or *Purgatorius* within the crown. *Protungulatum* is known from both Paleocene and Cretaceous formations (Archibald *et al.*, 2011), and as such, the presence of this taxon within crown Placentalia would be evidence that the origin of placental mammals pre‐dates the K/Pg boundary. Where *Protungulatum* is resolved as a stem placental, the conclusions are more equivocal, as neither a Cretaceous nor a Paleocene origin for placental mammals can be ruled out. Nevertheless, given that the earliest ‘condylarths’ are known from the first few hundred thousand years of the Paleocene (Lofgren *et al.*, 2004), and are consistently resolved not just within Placentalia but within Laurasiatheria, an explosive increase in evolutionary rate would be necessary for a radiation to occur entirely within the Paleocene. Estimates from extant taxa require a fivefold increase in background rates of morphological evolution to allow the placental mammal origin to be less than 66 million years ago (Beck & Lee, 2014). This new topology, with broad sampling of temporally relevant taxa, makes it possible to analyse the timing and rate of placental mammal divergences robustly.

Identifying the sister taxon to crown Placentalia is also relevant for dating its origin and estimating the effect of the mass extinction on mammal diversity. The sister taxon to crown Placentalia varied among analyses in this study, but was either a member of the now‐paraphyletic Leptictida in DP and CP, or a *Protungulatum–Purgatorius* clade in analyses where *Purgatorius* was not constrained as a stem primate. *Gypsonictops* is known from the Late Cretaceous, while the other two genera are Paleocene. The presence of Cimolestidae and *Leptictis* on the placental stem implies that, minimally, three lineages of eutherian mammals (Placentalia, Cimolestidae, and a subgroup of Leptictida) survived the end‐Cretaceous mass extinction; more if Placentalia had already begun to diverge in the Late Cretaceous.

Additionally, there are several well‐supported relationships within Placentalia that provide minimum estimates for the divergence of orders which, based simply on first‐appearance dates, differ markedly in some cases from previous estimates. The oldest perissodactyl, *Hyracotherium*, is known from the earliest Eocene (e.g. Smith & Smith, 2003), giving a minimum divergence date of Perissodactyla from its nearest relatives of 56 Ma. However, the earliest phenacodontid, *Tetraclaenodon*, is known from the Torrejonian (e.g. Scott, Spivak, & Sweet, 2013). With a close relationship found here between Perissodactyla and some members of Phenacodontidae, including *Tetraclaenodon*, the minimum divergence date of Perissodactyla from its closest extant relatives would be 63 Ma, in the Early Paleocene. Such changes to internal estimates of divergence dates will impact further on the predicted date of divergence of crown Placentalia. The deep nesting within Laurasiatheria of Periptychidae, one of the earliest definitively crown placental clades from the first faunal substage of the Paleocene, would seem to support the hypothesis that either a rapid increase in evolutionary rate took place, or the origin of placental mammals pre‐dated the end‐Cretaceous mass extinction.

## VII. CONCLUSIONS

1. The majority of members of Cimolestidae and Leptictida are here resolved as stem eutherians, and both are paraphyletic with respect to crown Placentalia. The hypothesised relationship between Cimolestidae and Pantodonta is therefore not supported here.
2. All ‘condylarth’ taxa are laurasiatherian, with no taxa favoured as a stem paenungulate. The origin of the afrotherian ‘ungulates’ therefore remains unresolved, and although some ‘ungulate’ taxa such as *Simpsonotus* are occasionally recovered on the stem of Afrotheria, the pattern is inconsistent and requires further analysis with a more representative sample of South American ungulates.
3. The closest relatives of crown perissodactyls are consistently found to be phenacodontids, although *Phenacodus* and *Meniscotherium* are more parsimoniously resolved with members of Hyopsodontidae, which are sometimes closer to Artiodactyla.
4. Periptychidae and Pantodonta are sister taxa, and are more closely related to Ferae and Chiroptera than to Perissodactyla or Artiodactyla.
5. Where there is lack of support for the relationships of Paleocene mammals, this is in large part due to the behaviour of a few highly unstable taxa such as *Lainodon*.
6. An Atlantogenata–Boreoeutheria split is favoured over Epitheria or Exafroplacentalia at the root of Placentalia. While relatively common in molecular systematics, this topology is rarely supported using maximum parsimony and morphological data.
7. No definitive crown‐placental mammal has yet been found from the Cretaceous, as *Protungulatum* is resolved as a stem eutherian, and therefore the Cretaceous occurrence of *Protungulatum* cannot be considered definitive proof of a Cretaceous origin for placental mammals.

## Supporting information


**Fig. S1.** Consensus topology deriving from the CU analysis.

**Fig. S2.** Consensus topology deriving from the DM analysis.

**Fig. S3.** Consensus topology deriving from the DP analysis.

**Fig. S4.** Consensus topology deriving from the CF analysis.

**Fig. S5.** Consensus topology deriving from the CM analysis.

**Fig. S6.** Consensus topology deriving from the CP analysis.

**Fig. S7.** Bremer support tree from the DF analysis.

**Fig. S8.** Bremer support tree from the DM analysis.

**Fig. S9.** Bremer support tree from the DP analysis.

**Fig. S10.** Bremer support tree from the CF analysis.

**Fig. S11.** Bremer support tree from the CM analysis.

**Fig. S12.** Bremer support tree from the CP analysis.

**Appendix S1.** Specimens used in the coding of this matrix, complete with specimen number, genus and species‐level affiliation, and the literature from which codings were assessed, where relevant, including the figure number.

**Appendix S2.** Matrix used for the phylogenetic analyses which resulted in the DU, DF, DM and DP sets of most‐parsimonious trees, formatted to be openable in TNT.

**Appendix S3.** Matrix used for the phylogenetic analyses which resulted in the CU, CF, CM and CP sets of most‐parsimonious trees, formatted to be openable in TNT.

**Appendix S4.** List of all 680 characters with descriptions of each character state, the proximal and ultimate sources of that character, and the treatment of that character – whether it is continuous or discrete, and ordered or unordered.

**Appendix S5.** Additional topologies recovered in the DM, CM, DP and CP analyses.

**Appendix S6.** List of unambiguous synapomorphies of commonly recovered clades.

## References

[brv12242-bib-0001] Agnolin, F. L. & Chimento, N. R. (2011). Afrotherian affinities for endemic South American “ungulates”. Mammalian Biology – Zeitschrift für Säugetierkunde 76, 101–108.

[brv12242-bib-0002] Alroy, J. (1999). The fossil record of North American mammals: evidence for a Paleocene evolutionary radiation. Systematic Biology 48, 107–118. 12078635 10.1080/106351599260472

[brv12242-bib-0003] Ameghino, F. (1902). Notices préliminaires sur des ongulés nouveaux des terrains crétacés de Patagonie. Boletín Academia Nacional de Ciencias (Argentina) 16, 350–426.

[brv12242-bib-0004] Antunes, M. T. , Sigogneau‐Russell, D. & Russell, D. E. (1986). Sur quelques dents de Mammifères du Crétacé supérieur de Taveiro, Portugal (Note préliminaire). Comptes Rendus de l'Académie des Sciences à Paris, Série II 303, 1247–1250.

[brv12242-bib-0005] Archibald, J. D. (1998). Archaic ungulates (“Condylarthra”) In Evolution of Tertiary Mammals of North America. Terrestrial Carnivores, Ungulates, and Ungulate‐like Mammals (eds Janis C. M., Scott K. M. and Jacobs L. L.), pp. 292–331. Cambridge University Press, Cambridge.

[brv12242-bib-0006] Archibald, J. D. (2011). Extinction and Radiation: How the Fall Of Dinosaurs Led to the Rise Of Mammals. Johns Hopkins University Press, Baltimore, Maryland, USA.

[brv12242-bib-0007] *Archibald, J. D. & Averianov, A. O. (2001). *Paranyctoides* and allies from the late Cretaceous of North America and Asia. Acta Palaeontologica Polonica 46, 533–551.

[brv12242-bib-0008] *Archibald, J. D. & Averianov, A. O. (2006). Late Cretaceous asioryctitherian eutherian mammals from Uzbekistan and phylogenetic analysis of Asioryctitheria. Acta Palaeontologica Polonica 51, 351–376.

[brv12242-bib-0009] *Archibald, J. D. & Averianov, A. O. (2011). Phylogenetic analysis, taxonomic revision and dental ontogeny of the Cretaceous Zhelestidae (Mammalia: Eutheria). Zoological Journal of the Linnean Society 164, 361–426.

[brv12242-bib-0010] Archibald, J. D. , Averianov, A. O. & Ekdale, E. G. (2001). Late Cretaceous relatives of rabbits, rodents, and other extant eutherian mammals. Nature 414, 62–65. 11689942 10.1038/35102048

[brv12242-bib-0011] Archibald, J. D. , Schoch, R. M. & Rigby, J. K. Jr. (1983). A new subfamily, Conacodontinae, and new species, *Conacodon kohlbergeri*, of the Periptychidae (Condylarthra, Mammalia). Postilla 191, 1–24.

[brv12242-bib-0012] Archibald, J. D. , Zhang, Y. , Harper, T. & Cifelli, R. L. (2011). *Protungulatum*, confirmed Cretaceous occurrence of an otherwise Paleocene eutherian (Placental?) mammal. Journal of Mammalian Evolution 18, 153–161.

[brv12242-bib-0013] Argot, C. (2013). Postcranial analysis of a carnivoran‐like archaic ungulate: the case of *Arctocyon primaevus* (Arctocyonidae, Mammalia) from the late Paleocene of France. Journal of Mammalian Evolution 20, 83–114.

[brv12242-bib-0014] Asher, R. J. (2007). A web‐database of mammalian morphology and a reanalysis of placental phylogeny. BMC Evolutionary Biology 7, 10. 17608930 10.1186/1471-2148-7-108 PMC1941728

[brv12242-bib-0015] Asher, R. J. , Geisler, J. H. & Sanchez‐Villagra, M. R. (2008). Morphology, paleontology, and placental mammal phylogeny. Systematic Biology 57, 311–317. 18432551 10.1080/10635150802033022

[brv12242-bib-0016] Asher, R. J. & Helgen, K. M. (2010). Nomenclature and placental mammal phylogeny. BMC Evolutionary Biology 10, 9. 20406454 10.1186/1471-2148-10-102 PMC2865478

[brv12242-bib-0017] Asher, R. J. , Lin, K. H. , Kardjilov, N. & Hautier, L. (2011). Variability and constraint in the mammalian vertebral column. Journal of Evolutionary Biology 24, 1080–1090. 21338435 10.1111/j.1420-9101.2011.02240.x

[brv12242-bib-0018] *Asher, R. J. , McKenna, M. C. , Emry, R. J. , Tabrum, A. R. & Kron, D. G. (2002). Morphology and relationships of *Apternodus* and other extinct, zalambdodont, placental mammals. Bulletin of the American Museum of Natural History 273, 1–117.

[brv12242-bib-0019] *Asher, R. J. , Meng, J. , Wible, J. R. , McKenna, M. C. , Rougier, G. W. , Dashzeveg, D. & Novacek, M. J. (2005). Stem lagomorpha and the antiquity of Glires. Science 307, 1091–1094. 15718468 10.1126/science.1107808

[brv12242-bib-0020] *Asher, R. J. , Novacek, M. J. & Geisler, J. H. (2003). Relationships of endemic African mammals and their fossil relatives based on morphological and molecular evidence. Journal of Mammalian Evolution 10, 131–194.

[brv12242-bib-0021] *Averianov, A. O. (1997). New late Cretaceous mammals of Southern Kazakhstan. Acta Palaeontologica Polonica 42, 243–256.

[brv12242-bib-0022] *Averianov, A. O. (2000). Mammals from the Mesozoic of Kirgizstan, Uzbekistan, Kazakhstan, and Tadzhikistan In The Age of Dinosaurs in Russia and Mongolia (eds Benton M. J., Shishkin M. A., Unwin D. M. and Kurochkin E. N.), pp. 627–652. Cambridge University Press, Cambridge.

[brv12242-bib-0023] *Averianov, A. O. & Archibald, J. D. (2013). New material and reinterpretation of the late Cretaceous eutherian mammal *Paranyctoides* from Uzbekistan. Acta Palaeontologica Polonica 58, 17–23.

[brv12242-bib-0024] *Bai, B. , Wang, Y. & Meng, J. (2010). New craniodental materials of *Litolophus gobiensis* (Perissodactyla, “Eomoropidae”) from Inner Mongolia, China, and phylogenetic analyses of Eocene chalicotheres. American Museum Novitates 3688, 1–27.

[brv12242-bib-0025] Bajpai, S. , Thewissen, J. G. M. & Sahni, A. (2009). The origin and early evolution of whales: macroevolution documented on the Indian Subcontinent. Journal of Biosciences 34, 673–686. 20009264 10.1007/s12038-009-0060-0

[brv12242-bib-0026] *Barnes, L. G. (1984). Fossil odontocetes (Mammalia: Cetacea) from the Almejas Formation, Isla Cedros, Mexico. PaleoBios 42, 1–46.

[brv12242-bib-0027] *Beard, K. C. & Dawson, M. R. (2009). Early Wasatchian mammals of the Red Hot local fauna, uppermost Tuscahoma Formation, Lauderdale County, Mississippi. Annals of the Carnegie Museum 78, 193–243.

[brv12242-bib-0028] *Beard, K. C. , Wang, Y. Q. , Meng, J. , Ni, X.‐J. , Gebo, D. L. & Li, C. K. (2010). Paleocene *Hapalodectes* (Mammalia, Mesonychia) from Subeng, Nei Mongol: further evidence of “East of Eden” dispersal at the Paleocene‐Eocene boundary. Vertebrata PalAsiatica 48, 375–389.

[brv12242-bib-0029] Beck, R. M. D. & Lee, M. S. Y. (2014). Ancient dates or accelerated rates? Morphological clocks and the antiquity of placental mammals. Proceedings of the Royal Society B: Biological Sciences 281 (doi: 10.1098/rspb.2014.1278). PMC4173679 25165770

[brv12242-bib-0030] Billet, G. (2010). New observations on the skull of *Pyrotherium* (Pyrotheria, Mammalia) and new phylogenetic hypotheses on South American ungulates. Journal of Mammalian Evolution 17, 21–59.

[brv12242-bib-0031] Bininda‐Emonds, O. R. P. , Cardillo, M. , Jones, K. E. , MacPhee, R. D. E. , Beck, R. M. D. , Grenyer, R. , Price, S. A. , Vos, R. A. , Gittleman, J. L. & Purvis, A. (2007). The delayed rise of present‐day mammals. Nature 446, 507–512. 17392779 10.1038/nature05634

[brv12242-bib-0032] de Blainville, H. M. D. (1841). Ostéographie et description iconographique des Mammifères récents et fossiles (Carnivores), J.B. Baillière et Fils, Paris.

[brv12242-bib-0033] *Bloch, J. I. , Secord, R. & Gingerich, P. D. (2004). Systematics and phylogeny of late Paleocene and early Eocene Palaeoryctinae (Mammalia, Insectivora) from the Clarks Fork and Bighorn basins, Wyoming. Papers in the Earth and Atmospheric Sciences 187, 119–154.

[brv12242-bib-0034] Bond, M. , Reguero, M. A. , Vizcaino, S. F. & Marenssi, S. A. (2006). A new 'South American ungulate' (Mammalia : Litopterna) from the Eocene of the Antarctic Peninsula. Cretaceous‐Tertiary High‐Latitude Palaeoenvironments: James Ross Basin Antartica 258, 163–176.

[brv12242-bib-0035] *Boyer, D. M. & Georgi, J. A. (2007). Cranial morphology of a pantolestid eutherian mammal from the Eocene Bridger Formation, Wyoming, USA: implications for relationships and habitat. Journal of Mammalian Evolution 14, 239–280.

[brv12242-bib-0036] Buckley, M. (2015). Ancient collagen reveals evolutionary history of the endemic South American 'ungulates'. Proceedings of the Royal Society B: Biological Sciences 282 (doi: 10.1098/rspb.2014.2671). PMC4426609 25833851

[brv12242-bib-0037] Butler, P. M. (1972). Some functional aspects of molar evolution. Evolution 26, 474. 28563057 10.1111/j.1558-5646.1972.tb01951.x

[brv12242-bib-0038] Carroll, R. L. (1988). Vertebrate Paleontology and Evolution. W.H. Freeman and Company, New York.

[brv12242-bib-0039] Chester, S. G. B. & Bloch, J. I. (2013). Systematics of Paleogene Micromomyidae (Euarchonta, Primates) from North America. Journal of Human Evolution 65, 109–142. 23850536 10.1016/j.jhevol.2013.04.006

[brv12242-bib-0040] Chester, S. G. B. , Bloch, J. I. , Boyer, D. M. & Clemens, W. A. (2015). Oldest known euarchontan tarsals and affinities of Paleocene *Purgatorius* to Primates. Proceedings of the National Academy of Sciences of the United States of America 112, 1487–1492. 25605875 10.1073/pnas.1421707112 PMC4321231

[brv12242-bib-0041] Chow, M. & Li, C.‐K. (1965). *Homogalax* and *Heptodon* of Shantung. Vertebrata PalAsiatica 9, 15–22.

[brv12242-bib-0042] Churakov, G. , Kriegs, J. O. , Baertsch, R. , Zemann, A. , Brosius, J. & Schmitz, J. (2009). Mosaic retroposon insertion patterns in placental mammals. Genome Research 19, 868–875. 19261842 10.1101/gr.090647.108 PMC2675975

[brv12242-bib-0043] *Cifelli, R. (1982). The petrosal structure of *Hyopsodus* with respect to that of some other ungulates, and its phylogenetic implications. Journal of Vertebrate Paleontology 56, 795–805.

[brv12242-bib-0044] Cifelli, R. (1983). The origin and affinities of the South American Condylarthra and early Tertiary Litopterna (Mammalia). American Museum Novitates 2772, 1–49.

[brv12242-bib-0045] *Cifelli, R. (1990). Cretaceous mammals of Southern Utah IV: eutherian mammals from the Wahweap (Aquilan) and Kaiparowits (Judithian) Formations. Journal of Vertebrate Paleontology 3, 346–360.

[brv12242-bib-0046] Cifelli, R. L. , Schaff, C. R. & McKenna, M. C. (1989). The relationships of the Arctostylopidae (Mammalia): new data and interpretation. Bulletin of the Museum of Comparative Zoology 152, 1–44.

[brv12242-bib-0047] Clemens, W. A. (2004). *Purgatorius* (Plesiadapiformes, Primates?, Mammalia), a Paleocene immigrant into northeastern Montana: stratigraphic occurrences and incisor proportions. Bulletin of Carnegie Museum of Natural History 36, 3–13.

[brv12242-bib-0048] Clemens, W. A. (2010). Were immigrants a significant part of the Earliest Paleocene mammalian fauna of the North American Western Interior? Vertebrata PalAsiatica 48, 285–307.

[brv12242-bib-0049] Colbert, E. H. (1933). The skull of *Dissopsalis carnifex pilgrim*, a Miocene creodont from India. American Museum Novitates 603, 1–8.

[brv12242-bib-0050] Colbert, E. H. & Morales, M. (1991). Creodonts and Carnivores In Evolution of the Vertebrates (eds Colbert E. H. and Morales M.). Wiley‐Liss, New York, 373–390.

[brv12242-bib-0051] *Coombs, M. C. & Coombs, W. P. Jr. (1977). Dentition of *Gobiohyus* and a re‐evaluation of the Helohyidae (Artiodactyla). Journal of Mammalogy 58, 291–308.

[brv12242-bib-0052] *Coombs, M. C. & Coombs, W. P. Jr. (1982). Anatomy of the ear region of four Eocene artiodactyls: *Gobiohyus*, ?*Helohyus*, *Diacodexis* and *Homacodon* . Journal of Vertebrate Paleontology 2, 219–236.

[brv12242-bib-0053] *Cooper, L. N. , Thewissen, J. G. M. , Bajpai, S. & Tiwari, B. N. (2012). Postcranial morphology and locomotion of the Eocene raoellid *Indohyus* (Artiodactyla: Mammalia). Historical Biology 24, 279–310.

[brv12242-bib-0054] Cope, E. D. (1884a). The Condylarthra. The American Naturalist 18, 790–805.

[brv12242-bib-0055] *Cope, E. D. (1884b). The Condylarthra (continued). The American Naturalist 18, 892–906.

[brv12242-bib-0056] Cope, E. D. (1884c). The Creodonta. The American Naturalist 18, 255–267.

[brv12242-bib-0057] Crusafont‐Pairo, M. & Golpe Posse, J. M. (1975). Les prosimiens de l'Eocene de la region preaxiale pyrenaique II: Adapidae. Colloque International CNRS: Problemes Actuels de Paleontologie‐Evolution des Vertebres 218, 851–860.

[brv12242-bib-0058] Darroch, S. A. F. , Webb, A. E. , Longrich, N. & Belmaker, J. (2014). Palaeocene‐Eocene evolution of beta diversity among ungulate mammals in North America. Global Ecology and Biogeography 23, 757–768.

[brv12242-bib-0059] Davalos, L. M. , Velazco, P. M. , Warsi, O. M. , Smits, P. D. & Simmons, N. B. (2014). Integrating incomplete fossils by isolating conflicting signal in saturated and non‐independent morphological characters. Systematic Biology 63, 582–600. 24817532 10.1093/sysbio/syu022

[brv12242-bib-0060] Dawson, M. R. (2012). *Coryphodon*, the northernmost Holarctic Paleogene pantodont (Mammalia), and its global wanderings. Swiss Journal of Palaeontology 131, 11–22.

[brv12242-bib-0061] De Bast, E. & Smith, T. (2013). Reassessment of the small arctocyonid *Prolatidens waudruae* from the early Paleocene of Belgium, and its phylogenetic relationships with ungulate‐like mammals. Journal of Vertebrate Paleontology 33, 964–976.

[brv12242-bib-0062] Delsuc, F. , Scally, M. , Madsen, O. , Stanhope, M. J. , de Jong, W. W. , Catzeflis, F. M. , Springer, M. S. & Douzery, E. J. P. (2002). Molecular phylogeny of living xenarthrans and the impact of character and taxon sampling on the placental tree rooting. Molecular Biology and Evolution 19, 1656–1671. 12270893 10.1093/oxfordjournals.molbev.a003989

[brv12242-bib-0063] Ding, S.‐Y. (1987). A Paleocene edentate from Nanxiong Basin, Guangdong. Palaeontologica Sinica New Series C 173, 1–118.

[brv12242-bib-0064] Domning, D. P. , Emry, R. J. , Portell, R. W. , Donovan, S. K. & Schindler, K. S. (1997). Oldest West Indian land mammal: rhinocerotoid ungulate from the Eocene of Jamaica. Journal of Vertebrate Paleontology 17, 638–641.

[brv12242-bib-0065] Eberle, J. J. , Rybczynski, N. & Greenwood, D. R. (2014). Early Eocene mammals from the Driftwood Canyon Provincial Park, northern British Columbia. Journal of Vertebrate Paleontology 34, 739–746.

[brv12242-bib-0066] Ekdale, E. G. , Archibald, J. D. & Averianov, A. O. (2004). Petrosal bones of placental mammals from the Late Cretaceous of Uzbekistan. Acta Palaeontologica Polonica 49, 161–176.

[brv12242-bib-0067] Filhol, H. (1892). Note sur un insectivore nouveau. Bulletin de la Société Philomathique de Paris 8, 134.

[brv12242-bib-0068] *Flynn, J. J. & Galiano, H. (1982). Phylogeny of early Tertiary Carnivora, with a description of a new species of *Protictis* from the Middle Eocene of Northwestern Wyoming. American Museum Novitates 2725, 1–64.

[brv12242-bib-0069] Flynn, J. J. & Wesley‐Hunt, G. D. (2005). Phylogeny and early diversification of the Carnivora In The Rise of Placental Mammals: Origins and Relationships of the Major Extant Clades (eds Archibald J. D. and Rose K.). John Hopkins University Press, Baltimore, 171–198.

[brv12242-bib-0070] *Fox, R. C. (1984). First North American record of the Paleocene primate *Saxonella* . Journal of Paleontology 66, 499–520.

[brv12242-bib-0071] *Fox, R. C. (1991). *Saxonella* (Plesiadapiformes, ?Primates) in North America: *S. naylori*, sp. nov., from the Late Paleocene of Alberta, Canada. Journal of Vertebrate Paleontology 11, 334–349.

[brv12242-bib-0072] *Fox, R. C. (1994). Composition of the holotype of the North American Late Cretaceous mammal *Cimolestes cerberoides* Lillegraven 1969. Journal of Paleontology 68, 910–911.

[brv12242-bib-0073] *Fox, R. C. (2011). An unusual early primate from the Paleocene Paskapoo Formation, Alberta, Canada. Acta Palaeontologica Polonica 56, 1–10.

[brv12242-bib-0074] Fox, R. C. & Scott, C. S. (2011). A new, early Puercan (Earliest Paleocene) species of *Purgatorius* (Plesiadapiformes, Primates) from Saskatchewan, Canada. Journal of Paleontology 85, 537–548.

[brv12242-bib-0075] Fox, R. C. , Scott, C. S. & Rankin, B. D. (2010). New early carnivoran specimens from the Puercan (Earliest Paleocene) of Saskatchewan, Canada. Journal of Paleontology 84, 1035–1039.

[brv12242-bib-0076] Fox, R. C. & Youzwyshyn, G. P. (1994). New primitive carnivorans (Mammalia) from the Paleocene of Western Canada, and their bearing on relationships of the order. Journal of Vertebrate Paleontology 14, 382–404.

[brv12242-bib-0077] Froehlich, D. J. (1999). Phylogenetic systematics of basal perissodactyls. Journal of Vertebrate Paleontology 19, 140–159.

[brv12242-bib-0078] Froehlich, D. J. (2002). Quo vadis *Eohippus*? The systematics and taxonomy of the early Eocene equids (Perissodactyla). Zoological Journal of the Linnean Society 134, 141–256.

[brv12242-bib-0079] Gaudin, T. J. (1999). The morphology of xenarthrous vertebrae (Mammalia: Xenarthra). Fieldiana Geology 41, 1–38. 10.1002/jmor.1052140105 1433308

[brv12242-bib-0080] Gaudin, T. J. , Emry, R. J. & Wible, J. R. (2009). The phylogeny of living and extinct pangolins (Mammalia, Pholidota) and associated taxa: a morphology based analysis. Journal of Mammalian Evolution 16, 235–305.

[brv12242-bib-0081] Gaudin, T. J. , Wible, J. R. , Hopson, J. A. & Turnbull, W. D. (1996). Reexamination of the morphological evidence for the cohort Epitheria (Mammalia, Eutheria). Journal of Mammalian Evolution 3, 31–79.

[brv12242-bib-0082] *Geisler, J. H. (2001). New morphological evidence for the phylogeny of Artiodactyla, Cetacea, and Mesonychidae. American Museum Novitates 3344, 1–53.

[brv12242-bib-0083] Geisler, J. H. & McKenna, M. C. (2007). A new species of mesonychian mammal from the lower Eocene of Mongolia and its phylogenetic relationships. Acta Palaeontologica Polonica 52, 189–212.

[brv12242-bib-0084] *Geisler, J. H. & Luo, Z. X. (1996). The petrosal and inner ear of *Herpetocetus* sp. (Mammalia, Cetacea) and their implications for the phylogeny and hearing of archaic mysticetes. Journal of Paleontology 70, 1045–1066.

[brv12242-bib-0085] *Geisler, J. H. & Luo, Z. X. (1998). Relationships of Cetacea to terrestrial ungulates and the evolution of cranial vasculature in Cete In The Emergence of Whales (ed. Thewissen J. G. M.), pp. 163–212. Springer, New York.

[brv12242-bib-0086] *Gentry, A. W. & Hooker, J. J. (1988). The phylogeny of the Artiodactyla In The Phylogeny and Classification of the Tetrapods (ed. Benton M. J.), pp. 235–272. Clarendon Press, Oxford.

[brv12242-bib-0087] *George, S. B. , Choate, J. R. & Genoways, H. H. (1986). Blarina brevicauda. Mammalogy Papers: University of Nebraska State Museum 95, 1–9.

[brv12242-bib-0088] *Gheerbrant, E. (1991). *Todralestes variabilis* n.g. n.sp., nouveau proteuthérien (Eutheria, Todralestidae fam. nov.) du Paléocène du Maroc. Comptes Rendus de l'Académie des Sciences à Paris Série II 312, 1249–1255.

[brv12242-bib-0089] *Gheerbrant, E. (2009). Paleocene emergence of elephant relatives and the rapid radiation of African ungulates. Proceedings of the National Academy of Sciences of the United States of America 106, 10717–10721. 19549873 10.1073/pnas.0900251106 PMC2705600

[brv12242-bib-0090] Gheerbrant, E. , Amaghzaz, M. , Bouya, B. , Goussard, F. & Letenneur, C. (2014). Ocepeia (Middle Paleocene of Morocco): the oldest skull of an afrotherian mammal. PLoS One 9, e89739. 24587000 10.1371/journal.pone.0089739 PMC3935939

[brv12242-bib-0091] *Gheerbrant, E. & Astibia, H. (1994). Un nouveau mammifère du Maastrichtien de Laño (Pays Basques espagnol). Comptes Rendus de l'Académie des Sciences à Paris, Série II 318, 1125–1131.

[brv12242-bib-0092] Gill, T. (1872). Arrangement of the Families of Mammals with Analytical Tables. Smithsonian Institution, Washington.

[brv12242-bib-0093] *Gingerich, P. D. (1980). *Tytthaena parrisi*, oldest known oxyaenid (Mammalia, Creodonta) from the Late Paleocene of Western North America. Journal of Paleontology 54, 570–576.

[brv12242-bib-0094] *Gingerich, P. D. (1981). Radiation of early Cenozoic Didymoconidae (Condylarthra, Mesonychia) in Asia, with a new genus from the early Eocene of Western North America. Journal of Mammalogy 62, 526–538.

[brv12242-bib-0095] *Gingerich, P. D. (1982). *Aaptoryctes* (Palaeoryctidae) and *Thelysia* (Palaeoryctidae?): new insectivorous mammals from the late Paleocene and early Eocene of Western North America. Contributions from the Museum of Paleontology, University of Michigan 26, 37–47.

[brv12242-bib-0096] *Gingerich, P. D. (1983a). Systematics of early Eocene Miacidae (Mammalia, Carnivora) in the Clark's Fork Basin, Wyoming. Contributions from the Museum of Paleontology, University of Michigan 26, 197–225.

[brv12242-bib-0097] *Gingerich, P. D. (1983b). New Adapisoricidae, Pentacodontidae, and Hyopsodontidae (Mammalia, Insectivora and Condylarhtra) from the late Paleocene of Wyoming and Colorado. Contributions from the Museum of Paleontology, University of Michigan 26, 227–255.

[brv12242-bib-0098] *Gingerich, P. D. (1987). Early Eocene bats (Mammalia, Chiroptera) and other vertebrates in freshwater limestones of the Willwood Formation, Clark's Fork Basin, Wyoming. Contributions from the Museum of Paleontology, University of Michigan 27, 275–320.

[brv12242-bib-0099] *Gingerich, P. D. (1990). Prediction of body mass in mammalian species from long bone lengths and diameters. Contributions from the Museum of Paleontology, University of Michigan 28, 79–92.

[brv12242-bib-0100] *Gingerich, P. D. (1994). New species of *Apheliscus*, *Haplomylus* and *Hyopsodus* (Mammalia, Condylarthra) from the late Paleocene of Southern Montana and early Eoceneof Northwestern Wyoming. Contributions from the Museum of Paleontology, University of Michigan 29, 119–134.

[brv12242-bib-0101] *Gingerich, P. D. & Deutsch, H. A. (1989). Systematics and evolution of early Eocene Hyaenodontidae (Mammalia, Creodonta) in the Clark's Fork Basin, Wyoming. Contributions from the Museum of Paleontology, University of Michigan 27, 319–327.

[brv12242-bib-0102] Gingerich, P. D. , ul Haq, M. , Zalmout, I. S. , Khan, I. H. & Malkani, M. S. (2001). Origin of whales from early artiodactyls: hands and feet of Eocene Protocetidae from Pakistan. Science 293, 2239–2242. 11567134 10.1126/science.1063902

[brv12242-bib-0103] *Gingerich, P. D. , Raza, S. M. , Arif, M. , Anwar, M. & Zhou, X. (1994). New whale from the Eocene of Pakistan and the origin of cetacean swimming. Nature 368, 844–847.

[brv12242-bib-0104] *Gingerich, P. D. & Russell, D. E. (1981). *Pakicetus inachus*, a new archaeocete (Mammalia, Cetacea) from the early‐Middle Eocene Kuldana Formation of Kohat (Pakistan). Contributions from the Museum of Paleontology, University of Michigan 25, 235–246.

[brv12242-bib-0105] Godinot, M. , Smith, T. & Smith, R. (1996). Life habitat and affinities of *Paschatherium* (Condylarthra, Hyopsodontidae) based on tarsal examination. Palaeovertebrata (Montpellier) 25, 225–242.

[brv12242-bib-0106] Goloboff, P. A. & Farris, J. S. (2001). Methods for quick consensus estimation. Cladistics 17, S26–S34.

[brv12242-bib-0107] Goloboff, P. A. , Farris, J. S. & Nixon, K. C. (2008). TNT, a free program for phylogenetic analysis. Cladistics 24, 774–786.

[brv12242-bib-0108] Goloboff, P. A. , Mattoni, C. I. & Quinteros, A. S. (2006). Continuous characters analyzed as such. Cladistics 22, 589–601. 10.1111/j.1096-0031.2006.00122.x 34892898

[brv12242-bib-0109] Goswami, A. (2010). Introduction to Carnivora In Carnivoran evolution: new views on phylogeny, form and function [Cambridge Studies in Morphology and Molecules: New Paradigms in Evolutionary Biology] (eds Goswami A. and Friscia A.), pp. 1–24. Cambridge University Press, Cambridge, UK.

[brv12242-bib-0110] Goswami, A. (2012). A dating success story: genomes and fossils converge on placental mammal origins. EvoDevo 3, 4. 22883371 10.1186/2041-9139-3-18 PMC3472198

[brv12242-bib-0111] Goswami, A. , Prasad, G. V. R. , Upchurch, P. , Boyer, D. M. , Seiffert, E. R. , Verma, O. , Gheerbrant, E. & Flynn, J. J. (2011). A radiation of arboreal basal eutherian mammals beginning in the late Cretaceous of India. Proceedings of the National Academy of Sciences of the United States of America 108, 16333–16338. 21930906 10.1073/pnas.1108723108 PMC3182738

[brv12242-bib-0112] Gould, F. D. H. & Rose, K. D. (2014). Gnathic and postcranial skeleton of the largest known arctocyonid 'condylarth' *Arctocyon mumak* (Mammalia, Procreodi) and ecomorphological diversity in Procreodi. Journal of Vertebrate Paleontology 34, 1180–1202.

[brv12242-bib-0113] Gregory, W. K. (1910). The Orders of Mammals. Bulletin of the American Museum of Natural History 27, 1–556.

[brv12242-bib-0114] Grossnickle, D. M. & Polly, P. D. (2013). Mammal disparity decreases during the Cretaceous angiosperm radiation. Proceedings of the Royal Society B: Biological Sciences 280 (doi: 10.1098/rspb.2013.2110). PMC3790494 24089340

[brv12242-bib-0115] Gunnell, G. F. & Gingerich, P. D. (1991). Systematics and evolution of late Paleocene and early Eocene Oxyaenidae (Mammalia, Creodonta) in the Clark's Fork Basin, Wyoming, USA. Contributions from the Museum of Paleontology University of Michigan 28, 141–179.

[brv12242-bib-0116] Hallstrom, B. M. & Janke, A. (2008). Resolution among major placental mammal interordinal relationships with genome data imply that speciation influenced their earliest radiations. BMC Evolutionary Biology 8 (doi: 10.1186/1471-2148-8-162). PMC2435553 18505555

[brv12242-bib-0117] Hallstrom, B. M. & Janke, A. (2010). Mammalian evolution may not be strictly bifurcating. Molecular Biology and Evolution 27, 2804–2816. 20591845 10.1093/molbev/msq166 PMC2981514

[brv12242-bib-0118] Hallstrom, B. M. , Kullberg, M. , Nilsson, M. A. & Janke, A. (2007). Phylogenomic data analyses provide evidence that Xenarthra and Afrotheria are sister groups. Molecular Biology and Evolution 24, 2059–2068. 17630282 10.1093/molbev/msm136

[brv12242-bib-0119] Hallstrom, B. M. , Schneider, A. , Zoller, S. & Janke, A. (2011). A genomic approach to examine the complex evolution of Laurasiatherian mammals. PLoS One 6(12), e28199 (doi: 10.1371/journal.pone.0028199). 22164244 PMC3229520

[brv12242-bib-0120] *Heinrich, R. E. (1997). Referral of *Miacis jepseni* Guthrie to *Oödectes* Wortman, and an assessment of phylogenetic relationships among early Eocene Miacidae (Mammalia:Carnivora). Journal of Paleontology 71, 1172–1178.

[brv12242-bib-0121] *Holbrook, L. T. (1999). The phylogeny and classification of tapiromorph perissodactyls (Mammalia). Cladistics 15, 331–350. 10.1111/j.1096-0031.1999.tb00270.x 34902952

[brv12242-bib-0122] Holbrook, L. T. & Lapergola, J. (2011). A new genus of perissodactyl (Mammalia) from the Bridgerian of Wyoming, with comments on basal perissodactyl phylogeny. Journal of Vertebrate Paleontology 31, 895–901.

[brv12242-bib-0123] Hooker, J. J. (2001). Tarsals of the extinct insectivoran family Nyctitheriidae (Mammalia): evidence for archontan relationships. Zoological Journal of the Linnean Society 132, 501–529.

[brv12242-bib-0124] Hooker, J. J. (2013). Origin and evolution of the Pseudorhyncocyonidae, a European Paleogene family of insectivorous placental mammals. Palaeontology 56, 807–835.

[brv12242-bib-0125] Hooker, J. J. (2014). New postcranial bones of the extinct mammalian family Nyctitheriidae (Paleogene, UK): primitive euarchontans with scansorial locomotion. Palaeontologica Electronica 17(3), 47A.

[brv12242-bib-0126] Hooker, J. J. & Dashzeveg, D. (2003). Evidence for direct mammalian faunal interchange between Europe and Asia near the Paleocene‐Eocene boundary. Geological Society of America, Special Paper 369, 479–500.

[brv12242-bib-0127] Hooker, J. J. & Russell, D. E. (2012). Early Palaeogene Louisinidae (Macroscelidea, Mammalia), their relationships and north European diversity. Zoological Journal of the Linnean Society 164, 856–936.

[brv12242-bib-0128] *Horovitz, I. (2000). The tarsus of *Ukhaatherium nessovi* (Eutheria, Mammalia) from the Late Cretaceous of Mongolia: an appraisal of the evolution of the ankle in basal therians. Journal of Vertebrate Paleontology 20, 547–560.

[brv12242-bib-0129] *Horovitz, I. (2003). Postcranial skeleton of *Ukhaatherium nessovi* (Eutheria, Mammalia) from the Late Cretaceous of Mongolia. Journal of Vertebrate Paleontology 23, 857–868.

[brv12242-bib-0130] Horovitz, I. (2004). Eutherian mammal systematics and the origins of South American ungulates as based on postcranial osteology. Bulletin of Carnegie Museum of Natural History 36, 63–79.

[brv12242-bib-0131] *Horovitz, I. & Sánchez‐Villagra, M. R. (2003). A morphological analysis of marsupial mammal higher‐level phylogenetic relationships. Cladistics 19, 181–212.

[brv12242-bib-0132] *Horovitz, I. , Storch, G. & Martin, T. (2005). Ankle structure in Eocene pholidotan mammal *Eomanis krebsi* and its taxonomic implications. Acta Palaeontologica Polonica 50, 545–548.

[brv12242-bib-0133] Hu, J. , Zhang, Y. & Yu, L. (2012). Summary of Laurasiatheria (Mammalia) Phylogeny. Zoological Research 33, E65–E74. 23266984 10.3724/SP.J.1141.2012.E05-06E65

[brv12242-bib-0134] Hunt, R. M. & Tedford, R. H. (1993). Phylogenetic relationships within the aeluroid Carnivora and implications of their temporal and geographic distribution In Mammal Phylogeny:Placentals (eds Szalay F. S., Novacek M. J. and McKenna M. C.), pp. 53–73. Springer‐Verlag, New York, USA.

[brv12242-bib-0135] Jablonski, D. & Chaloner, W. G. (1994). Extinctions in the fossil record. Philosophical Transactions of the Royal Society of London Series B: Biological Sciences 344, 11–16.

[brv12242-bib-0136] Jepsen, G. L. (1937). A Paleocene rodent, *Paramys atavus* . Proceedings of the American Philosophical Society 78, 291–301.

[brv12242-bib-0137] Jepsen, G. L. (1966). Early Eocene bat from Wyoming. Science 154, 1333. 17770307 10.1126/science.154.3754.1333

[brv12242-bib-0138] *Kalthoff, D. C. , Rose, K. D. & von Koenigswald, W. (2011). Dental microstructure in *Palaeanodon* and *Tubulodon* (Palaeanodonta) and bioerosional tunnelling as a widespread phenomenon in fossil mammal teeth. Journal of Vertebrate Paleontology 31, 1303–1313.

[brv12242-bib-0139] *Kielan‐Jaworowska, Z. (1968). Preliminary data on the Upper Cretaceous eutherian mammals from Bayn Dzak, Gobi Desert. Acta Palaeontologica Polonica 19, 171–191.

[brv12242-bib-0140] *Kielan‐Jaworowska, Z. (1975). Preliminary description of two new eutherian genera from the Late Cretaceous of Mongolia. Acta Palaeontologica Polonica 33, 1–13.

[brv12242-bib-0141] Kielan‐Jaworowska, Z. , Bown, T. M. & Lillegraven, J. A. (1979). Eutheria In Mesozoic Mammals: the First Two‐Thirds of Mammalian History (eds Lillegraven J. A., Kielan‐Jaworowska Z. and Clemens W. A.), pp. 221–258. University of California Press, Berkeley.

[brv12242-bib-0142] Kielan‐Jaworowska, Z. , Cifelli, R. L. & Luo, Z.‐X. (2004). Mammals from the Age of Dinosaurs: Origin, Evolutions, and Structure. Columbia University Press, New York.

[brv12242-bib-0143] *Kihm, A. J. & Schumaker, K. K. (2008). *Domnina* (Mammalia, Soricomorpha) from the latest Eocene (Chadronian) Medicine Pole Hills Local Fauna of North Dakota. Paludicola 7, 26–36.

[brv12242-bib-0144] Kondrashov, P. (2009). Postcranial adaptations of European arctocyonids (Mammalia, Arctocyonidae). Journal of Vertebrate Paleontology 29, 128A.

[brv12242-bib-0145] Kondrashov, P. E. & Lucas, S. G. (2004). *Oxyclaenus* from the Early Paleocene of New Mexico and the status of the Oxyclaeninae (Mammalia, Arctocyonidae). Bulletin of the New Mexico Museum of Natural History and Science 26, 21–32.

[brv12242-bib-0146] *Kondrashov, P. E. & Lucas, S. G. (2006). Early Paleocene (Puercan and Torrejonian) archaic ungulates (Condylarthra, Procreodi and Acreodi) of the San Juan Basin, New Mexico In Fossils from Federal Lands, New Mexico Museum of Natural History and Science Bulletin (eds Lucas S. G., Spielmann J. A., Hester P. M., Kenworthy J. P. and Santiccui V. L.), pp. 84–97. New Mexico Museum of Natural History, New Mexico.

[brv12242-bib-0147] Kondrashov, P. E. & Lucas, S. G. (2012). Nearly complete skeleton of *Tetraclaenodon* (Mammalia, Phenacodontidae) from the early Paleocene of New Mexico: morpho‐functional analysis. Journal of Paleontology 86, 25–43.

[brv12242-bib-0148] *Korth, W. W. (1988). *Paramys compressidens* Peterson and the systematic relationships of the species of *Paramys* (Paramyinae, Ischyromyidae). Journal of Paleontology 62, 468–471.

[brv12242-bib-0149] Kuntner, M. , May‐Collado, L. J. & Agnarsson, I. (2011). Phylogeny and conservation priorities of afrotherian mammals (Afrotheria, Mammalia). Zoologica Scripta 40, 1–15.

[brv12242-bib-0150] Ladevèze, S. , Missiaen, P. & Smith, T. (2010). First skull of *Orthaspidotherium edwardsi* (Mammalia, “Condylarthra”) from the Late Paleocene of Berru (France) and phylogenetic affinities of the enigmatic European family Pleuraspidotheriidae. Journal of Vertebrate Paleontology 30, 1559–1578.

[brv12242-bib-0151] Lee, M. S. Y. & Camens, A. B. (2009). Strong morphological support for the molecular evolutionary tree of placental mammals. Journal of Evolutionary Biology 22, 2243–2257. 19780874 10.1111/j.1420-9101.2009.01843.x

[brv12242-bib-0152] Leidy, J. (1868). Notice of some remains of extinct Insectivora from Dakota. Proceedings of the Academy of Natural Sciences of Philadelphia 20, 196–197.

[brv12242-bib-0153] *Lessertisseur, J. & Saban, R. (1967). Squelette appendiculaire In Traité de Zoologie I (ed. Grassé P.‐P.). Masson & Cie, Paris, 709–1078.

[brv12242-bib-0154] *Lihoreau, F. , Ducrocq, S. , Antoine, P.‐O. , Vianey‐Liaud, M. , Rafaÿ, S. , Garcia, G. & Valentin, X. (2009). First complete skulls of *Elomeryx crispus* (Gervais 1849) and of *Protaceratherium albingense* (Roman 1912) from a new Oligocene locality near Moissac (SW France). Journal of Vertebrate Paleontology 29, 242–253.

[brv12242-bib-0155] Lillegraven, J. A. (1969). Latest Cretaceous mammals of the upper part of the Edmonton Formation of Alberta, Canada, and review of the marsupial‐placental dichotomy in mammalian evolution. University of Kansas Paleontological Contributions 50, 1–122.

[brv12242-bib-0156] *Lillegraven, J. A. (1976). A new genus of therian mammal from the Late Cretaceous “El Gallo Formation”, Baja California, Mexico. Journal of Paleontology 50, 437–443.

[brv12242-bib-0157] *Lillegraven, J. A. , McKenna, M. C. & Krishtalka, L. (1981). Evolutionary relationships of Middle Eocene and younger species of *Centetodon* (Mammalia, Insectivora, Geolabididae) with a description of the dentition of *Ankylodon* (Adapisoricidae). University of Wyoming Publications 45, 1–115.

[brv12242-bib-0158] *Lloyd, K. J. & Eberle, J. J. (2008). A new talpid from the late Eocene of North America. Acta Palaeontologica Polonica 53, 539–543.

[brv12242-bib-0159] Lofgren, D. L. , Lillegraven, J. A. , Clemens, W. A. , Gingerich, P. D. & Williamson, T. E. (2004). Paleocene biochronology: the Puercan through Clarkforkian land mammal ages In Late Cretaceous and Cenozoic Mammals of North America: Biostratigraphy and Geochronology (ed. Woodburne M. O.), pp. 43–105. Columbia University Press, New York.

[brv12242-bib-0160] Lopatin, A. V. (2001). The skull structure of *Archaeoryctes euryalis* sp. nov. (Didymoconidae, Mammalia) from the Paleocene of Mongolia, with comments on the the taxonomic position of the family. Paleontological Journal 3, 97–107.

[brv12242-bib-0161] Lopatin, A. V. (2006). Early Paleogene insectivore mammals of Asia and establishment of the major groups of Insectivora. Paleontological Journal 40, S205–S405.

[brv12242-bib-0162] Lopatin, A. V. & Averianov, A. O. (2008). The earliest lagomorph (Lagomorpha, Mammalia) from the basal Eocene of Mongolia. Proceedings of the Academy of Sciences of the USSR, Biological Sciences 419, 131–132. 10.1134/s001249660802018x 18536282

[brv12242-bib-0163] *López‐Martinez, N. & Peláez‐Campomanus, P. (1999). New mammals from south central Pyrenees (Tremp Formation, Spain) and their bearing on Late Paleocene marine‐continental correlations. Bulletin du Sociéte Géologique de la France 170, 681–696.

[brv12242-bib-0164] *Lucas, S. G. & O'Neill, F. M. (1981). Occurrence of *Pantolambda* (Mammalia, Pantodonta) in the Torrejonian *Deltatherium* “Zone”, San Juan Basin, New Mexico. American Journal of Science 281, 187–191.

[brv12242-bib-0165] *Luo, Z. X. (1991). Variability of dental morphology and the relationships of the earliest arctocyonid species. Journal of Vertebrate Paleontology 11, 452–471.

[brv12242-bib-0166] Luo, Z. X. (2007). Transformation and diversification in early mammal evolution. Nature 450, 1011–1019. 18075580 10.1038/nature06277

[brv12242-bib-0167] *Luo, Z. X. & Wible, J. R. (2005). A Late Jurassic digging mammal and early mammalian diversification. Science 308, 103–107. 15802602 10.1126/science.1108875

[brv12242-bib-0168] *Maas, M. C. , Thewissen, J. G. M. , Sen, S. , Kazanci, N. & Kappelman, J. (2001). Enigmatic new ungulates from the early middle Eocene of central Anatolia, Turkey. Journal of Vertebrate Paleontology 21, 578–590.

[brv12242-bib-0169] MacFadden, B. J. & Shockey, B. J. (1997). Ancient feeding ecology and niche differentiation of Pleistocene mammalian herbivores from Tarija, Bolivia: morphological and isotopic evidence. Paleobiology 23, 77–100.

[brv12242-bib-0170] MacIntyre, G. T. (1966). The Miacidae (Mammalia, Carnivora): the systematics of *Ictidopappus* and *Protictis* . Bulletin of the American Museum of Natural History 131, 117–209.

[brv12242-bib-0171] *MacPhee, R. D. E. (1994). Morphology, adaptations, and relationships of *Plesiorycteropus*, and a diagnosis of a new order of eutherian mammals. Bulletin of the American Museum of Natural History 220, 1–214.

[brv12242-bib-0172] Madsen, O. , Scally, M. , Douady, C. J. , Kao, D. J. , DeBry, R. W. , Adkins, R. , Amrine, H. M. , Stanhope, M. J. , de Jong, W. W. & Springer, M. S. (2001). Parallel adaptive radiations in two major clades of placental mammals. Nature 409, 610–614. 11214318 10.1038/35054544

[brv12242-bib-0173] Malia, M. J. , Lipscomb, D. L. & Allard, M. W. (2003). The misleading effects of composite taxa in supermatrices. Molecular Phylogenetics and Evolution 27, 522–527. 12742756 10.1016/s1055-7903(03)00020-4

[brv12242-bib-0174] Mannion, P. D. , Upchurch, P. , Barnes, R. N. & Mateus, O. (2013). Osteology of the Late Jurassic Portuguese sauropod dinosaur *Lusotitan atalaiensis* (Macronaria) and the evolutionary history of basal titanosauriforms. Zoological Journal of the Linnean Society 168, 98–206.

[brv12242-bib-0175] Manz, C. L. , Chester, S. G. B. , Bloch, J. I. , Silcox, M. T. & Sargis, E. J. (2015). New partial skeletons of Palaeocene Nyctitheriidae and evaluation of proposed euarchontan affinities. Biology Letters 11, 20140911–20140911. 25589486 10.1098/rsbl.2014.0911 PMC4321154

[brv12242-bib-0176] Matthew, W. D. (1915). A revision of the lower Eocene Wasatch and Wind River faunas: part I – Order Ferae (Carnivora) Suborder Creodonta. Bulletin of the American Museum of Natural History 34, 4–103.

[brv12242-bib-0177] Matthew, W. D. , Granger, W. & Simpson, G. G. (1929). Additions to the fauna of the Gashato Formation of Mongolia. American Museum Novitates 376, 1–12.

[brv12242-bib-0178] *McKenna, M. C. (1968). *Leptacodon*, an American Paleocene nyctithere (Mammalia, Insectivora). American Museum Novitates 2317, 1–12.

[brv12242-bib-0179] McKenna, M. C. (1975). Towards a phylogenetic classification of the Mammalia In Phylogeny of the Primates: a Multidisciplinary Approach (eds Luckett W. and Szalay F.), pp. 21–46. Plenum, New York.

[brv12242-bib-0180] McKenna, M. C. & Bell, S. K. (1997). Classification of Mammals Above the Species Level. Columbia University Press, New York.

[brv12242-bib-0181] Meehan, T. J. & Martin, L. D. (2010). New leptictids (Mammalia: lnsectivora) from the early Oligocene of Nebraska, USA. Neues Jahrbuch Fur Geologie Und Palaontologie‐Abhandlungen 256, 99–107.

[brv12242-bib-0182] Meehan, T. J. & Wilson, R. W. (2002). New viverravids from the Torrejonian (Middle Paleocene) of Kutz Canyon, New Mexico and the oldest skull of the Order Carnivora. Journal of Paleontology 76, 1091–1101.

[brv12242-bib-0183] Meng, J. , Suyin, T. & Schiebout, J. A. (1995). The cranial morphology of an early Eocene didymoconid (Mammalia, Insectivora). Journal of Vertebrate Paleontology 14, 534–551.

[brv12242-bib-0184] Meng, J. & Wyss, A. R. (2001). The morphology of *Tribosphenomys* (Rodentiaformes, Mammalia): phylogenetic implications for basal Glires. Journal of Mammalian Evolution 8, 1–71.

[brv12242-bib-0185] *Mehta, S. K. & Jolly, A. (1989). *Leptomeryx*, an Oligocene artiodactyl from the Lower Murree of Sial Sui (Kalakot Tehsil), District Rajauri, Jammu and Kashmir. Current Science 58, 625–627.

[brv12242-bib-0186] *Meng, J. , Hu, Y. & Li, C. (2003). The osteology of *Rhombomylus* (Mammalia, Glires): implications for phylogeny and evolution of Glires. Bulletin of the American Museum of Natural History 275, 1–247.

[brv12242-bib-0187] *Meng, J. , Wyss, A. R. , Dawson, M. R. & Zhai, R. (1994). Primitive fossil rodent from Inner Mongolia and its implications for mammalian phylogeny. Nature 370, 134–136. 8022481 10.1038/370134a0

[brv12242-bib-0188] *Middleton, M. D. & Dewar, E. W. (2004). New mammals from the early Paleocene Littleton Fauna (Denver Formation, Colorado). New Mexico Museum of Natural History Science Bulletin 26, 51–80.

[brv12242-bib-0189] Missiaen, P. , Escarguel, G. , Hartenberger, J.‐L. & Smith, T. (2012). A large new collection of Palaeostylops from the Paleocene of the Flaming Cliffs area (Ulan‐Nur Basin, Gobi Desert, Mongolia), and an evaluation of the phylogenetic affinities of Arctostylopidae (Mammalia, Gliriformes). Geobios 45, 311–322.

[brv12242-bib-0190] Morgan, C. C. , Foster, P. G. , Webb, A. E. , Pisani, D. , McInerney, J. O. & O'Connell, M. J. (2013). Heterogeneous models place the root of the placental mammal phylogeny. Molecular Biology and Evolution 30, 2145–2156. 23813979 10.1093/molbev/mst117 PMC3748356

[brv12242-bib-0191] Morlo, M. , Gunnell, G. & Polly, P. D. (2009). What, if not nothing, is a creodont? Phylogeny and classification of Hyaenodontida and other former creodonts. Journal of Vertebrate Paleontology 29, 152A–152A.

[brv12242-bib-0192] de Muizon, C. & Cifelli, R. L. (2000). The “condylarths” (archaic Ungulata, Mammalia) from the early Palaeocene of Tiupampa (Bolivia): implications on the origin of the South American ungulates. Geodiversitas 22, 47–48.

[brv12242-bib-0193] *de Muizon, C. & Marshall, L. G. (1992). *Alcidedorbignya inopinata* (Mammalia: Pantodonta) from the early Paleocene of Bolivia: phylogenetic and paleobiogeographic implications. Journal of Paleontology 66, 499–520.

[brv12242-bib-0194] Murphy, W. J. , Pringle, T. H. , Crider, T. A. , Springer, M. S. & Miller, W. (2007). Using genomic data to unravel the root of the placental mammal phylogeny. Genome Research 17, 413–421. 17322288 10.1101/gr.5918807 PMC1832088

[brv12242-bib-0195] *Nessov, L. A. , Archibald, J. D. & Kielan‐Jaworowska, Z. (1998). Ungulate‐like mammals from the late Cretaceous of Uzbekistan and a phylogenetic analysis of Ungulatomorpha. Bulletin of the Carnegie Museum of Natural History 34, 40–88.

[brv12242-bib-0196] Nie, W. , Fu, B. , O'Brien, P. C. M. , Wang, J. , Su, W. , Tanomtong, A. , Volobouev, V. , Ferguson‐Smith, M. A. & Yang, F. (2008). Flying lemurs – the 'flying tree shrews'? Molecular cytogenetic evidence for a Scandentia‐Dermoptera sister clade. BMC Biology 6, 18. 18452598 10.1186/1741-7007-6-18 PMC2386441

[brv12242-bib-0197] Nishihara, H. , Hasegawa, M. & Okada, N. (2006). Pegasoferae, an unexpected mammalian clade revealed by tracking ancient retroposon insertions. Proceedings of the National Academy of Sciences of the United States of America 103, 9929–9934. 16785431 10.1073/pnas.0603797103 PMC1479866

[brv12242-bib-0198] Nishihara, H. , Maruyama, S. & Okada, N. (2009). Retroposon analysis and recent geological data suggest near‐simultaneous divergence of the three superorders of mammals. Proceedings of the National Academy of Sciences of the United States of America 106, 5235–5240. 19286970 10.1073/pnas.0809297106 PMC2655268

[brv12242-bib-0199] Novacek, M. J. (1986). The skull of leptictid insectivorans and the higher‐level classification of eutherian mammals. Bulletin of the American Museum of Natural History 183, 1–111.

[brv12242-bib-0200] *Novacek, M. J. (1987). Auditory features and affinities of the Eocene bats *Icaronycteris* and *Palaeochiropteryx* (Microchiroptera, *incertae sedis*). American Museum Novitates 2877, 1–18.

[brv12242-bib-0201] Novacek, M. J. (1992). Mammalian phylogeny – shaking the tree. Nature 356, 121–125. 1545862 10.1038/356121a0

[brv12242-bib-0202] *Novacek, M. J. , Bown, T. M. & Schankler, D. (1985). On the classification of the early Tertiary Erinaceomorpha (Insectivora, Mammalia). American Museum Novitates 2813, 1–22.

[brv12242-bib-0203] *Novacek, M. J. & Wyss, A. R. (1986). Higher‐level relationships of the recent eutherian orders: morphological evidence. Cladistics 2, 257–287. 10.1111/j.1096-0031.1986.tb00463.x 34949071

[brv12242-bib-0204] *Nummela, S. , Taseer Hussain, S. & Thewissen, J. G. M. (2006). Cranial anatomy of Pakicetidae (Cetacea, Mammalia). Journal of Vertebrate Paleontology 26, 746–759.

[brv12242-bib-0205] O'Leary, M. A. (1998). Morphology of the humerus of *Hapalodectes* (Mammalia, Mesonychia). American Museum Novitates 3242, 1–6.

[brv12242-bib-0206] O'Leary, M. A. , Bloch, J. I. , Flynn, J. J. , Gaudin, T. J. , Giallombardo, A. , Giannini, N. P. , Goldberg, S. L. , Kraatz, B. P. , Luo, Z. X. , Meng, J. , Ni, X. J. , Novacek, M. J. , Perini, F. A. , Randall, Z. S. , Rougier, G. W. , Sargis, E. J. , Silcox, M. T. , Simmons, N. B. , Spaulding, M. , Velazco, P. M. , Weksler, M. , Wible, J. R. & Cirranello, A. L. (2013). The placental mammal ancestor and the post‐K‐Pg radiation of placentals. Science 339, 662–667. 23393258 10.1126/science.1229237

[brv12242-bib-0207] *O'Leary, M. A. & Gatesy, J. (2008). Impact of increased character sampling on the phylogeny of Cetartiodactyla (Mammalia): combined analyses including fossils. Cladistics 24, 397–442. 10.1111/j.1096-0031.2007.00187.x 34879630

[brv12242-bib-0208] *O'Leary, M. A. & Geisler, J. H. (1999). The position of Cetacea within Mammalia: phylogenetic analysis of morphological data from extinct and extant taxa. Systematic Biology 48, 455–490. 12066291 10.1080/106351599260102

[brv12242-bib-0209] O'Leary, M. A. & Rose, K. D. (1995). Postcranial skeleton of the early Eocene mesonychid *Pachyaena* (Mammalia: Mesonychia). Journal of Vertebrate Paleontology 15, 401–430.

[brv12242-bib-0210] Onuma, M. , Cao, Y. , Hasegawa, M. & Kusakabe, S. (2000). A close relationship of Chiroptera with Eulipotyphla (core insectivora) suggested by four mitochondrial genes. Zoological Science 17, 1327–1332.

[brv12242-bib-0211] Orliac, M. J. , Argot, C. & Gilissen, E. (2012a). Digital cranial endocast of *Hyopsodus* (Mammalia, “Condylarthra”): a case of Paleogene terrestrial echolocation? PLoS One 7, e30000. 22347998 10.1371/journal.pone.0030000 PMC3277592

[brv12242-bib-0212] Orliac, M. J. , Benoit, J. & O'Leary, M. A. (2012b). The inner ear of *Diacodexis*, the oldest artiodactyl mammal. Journal of Anatomy 221, 417–426. 22938073 10.1111/j.1469-7580.2012.01562.x PMC3482349

[brv12242-bib-0213] Osborn, H. F. (1898). Remounted skeleton of *Phenacodus primaevus*; comparison with *Euprotogonia* . Bulletin of the American Museum of Natural History 10, 159–165.

[brv12242-bib-0214] Osborn, H. F. (1902). The law of adaptive radiation. American Naturalist 36, 353–363.

[brv12242-bib-0215] Osborn, H. F. (1924). *Andrewsarchus*, giant mesonychid of Mongolia. American Museum Novitates 146, 1–5.

[brv12242-bib-0216] Osborn, H. F. & Earle, C. (1895). Fossil mammals of the Puerco bed. Collection of 1892. Bulletin of the American Museum of Natural History 7, 1–70.

[brv12242-bib-0217] Polly, P. D. (1994). What, if anything, is a creodont? Journal of Vertebrate Paleontology 14, 42A.

[brv12242-bib-0218] Polly, P. D. (1996). The skeleton of *Gazinocyon vulpeculus* gen. et comb. nov. and the cladistic relationships of Hyaenodontidae (Eutheria, Mammalia). Journal of Vertebrate Paleontology 16, 303–319.

[brv12242-bib-0219] Prasad, A. B. , Allard, M. W. , Green, E. D. & Program, N. C. S. (2008). Confirming the phylogeny of mammals by use of large comparative sequence data sets. Molecular Biology and Evolution 25, 1795–1808. 18453548 10.1093/molbev/msn104 PMC2515873

[brv12242-bib-0220] Prothero, D. R. (1994). The Eocene‐Oligocene Transition: Paradise Lost, Cambridge University Press, New York, New York, USA.

[brv12242-bib-0221] Prothero, D. R. (1998). The chronological, climatic, and paleogeographic background to North American mammalian evolution In Evolution of Tertiary Mammals of North America, Terrestrial Carnivores, Ungulates, and Ungulatelike Mammals (Volume 1, eds Janis C. M., Scott K. M. and Jacobs L. L.), pp. 9–36. Cambridge University Press, Cambridge.

[brv12242-bib-0222] Prothero, D. R. , Manning, E. M. & Fischer, M. (1988). The Phylogeny of the Ungulates In The Phylogeny and Classification of the Tetrapods, Mammals (Volume II, ed. Benton M. J.), pp. 201–234. Clarendon, Oxford.

[brv12242-bib-0223] Pyron, R. A. & Burbrink, F. T. (2012). Trait‐dependent diversification and the impact of palaeontological data on evolutionary hypothesis testing in New World ratsnakes (tribe Lampropeltini). Journal of Evolutionary Biology 25, 497–508. 22226034 10.1111/j.1420-9101.2011.02440.x

[brv12242-bib-0224] *Qi, T. , Zong, G. & Wang, Y. (1989). Discovery of *Lushilagus* and *Miacis* in Jiangsu and its zoogeographical significance. Vertebrata PalAsiatica 29, 59–63.

[brv12242-bib-0225] Radinsky, L. B. (1965). Evolution of the tapiroid skeleton from *Heptodon* to *Tapirus* . Bulletin of the Museum of Comparative Zoology, Harvard University 134, 69–106.

[brv12242-bib-0226] Radinsky, L. B. (1966). Adaptive radiation of phenacodontid condylarths and origin of Perissodactyla. Evolution 20, 408. 28562971 10.1111/j.1558-5646.1966.tb03375.x

[brv12242-bib-0227] Rae, T. C. (1998). The logical basis for the use of continuous characters in phylogenetic systematics. Cladistics 14, 221–228. 10.1111/j.1096-0031.1998.tb00335.x 34905828

[brv12242-bib-0228] Rage, J. C. , Buffetaut, E. , Buffetauttong, H. , Chaimanee, Y. , Ducrocq, S. , Jaeger, J. J. & Suteethorn, V. (1992). A colubrid snake in the Late Eocene of Thailand ‐ the oldest known Colubridae (Reptilia, Serpentes). Comptes Rendus de l'Académie des Sciences à Paris, Serie II 314, 1085–1089.

[brv12242-bib-0229] Raia, P. , Carotenuto, F. , Passaro, F. , Piras, P. , Fulgione, D. , Werdelin, L. , Saarinen, J. & Fortelius, M. (2013). Rapid action in the Palaeogene, the relationship between phenotypic and taxonomic diversification in Coenozoic mammals. Proceedings of the Royal Society B: Biological Science 280, 1–7. 10.1098/rspb.2012.2244 PMC3574440 23173207

[brv12242-bib-0230] Raj Pant, S. , Goswami, A. & Finarelli, J. A. (2014). Complex body size trends in the evolution of sloths (Xenarthra: Pilosa). BMC Evolutionary Biology 14, 184. 25319928 10.1186/s12862-014-0184-1 PMC4243956

[brv12242-bib-0231] Rana, R. S. & Wilson, G. P. (2003). New Late Cretaceous mammals from the Intertrappean beds of Rangapur, India and paleobiogeographic framework. Acta Palaeontologica Polonica 48, 331–348.

[brv12242-bib-0232] dos Reis, M. , Inoue, J. , Hasegawa, M. , Asher, R. J. , Donoghue, P. C. J. & Yang, Z. (2012). Phylogenomic datasets provide both precision and accuracy in estimating the timescale of placental mammal phylogeny. Proceedings of the Royal Society B: Biological Sciences 279, 3491–3500. 10.1098/rspb.2012.0683 PMC3396900 22628470

[brv12242-bib-0233] Rigby, J. K. Jr. (1980). Swain quarry of the Fort Union Formation, middle Paleocene (Torrejonian), Carbon County, Wyoming: geologic setting and mammalian fauna. Evolutionary Monographs 3, 1–178.

[brv12242-bib-0234] Robinson, P. , Gunnell, G. F. , Walsh, S. L. , Clyde, W. C. , Storer, J. E. , Stucky, R. K. , Froehlich, D. J. , Ferrusquia‐Villafranca, I. & McKenna, M. C. (2004). Wasatchian through Duchesnean biochronology In Late Cretaceous and Cenozoic Mammals of North America: Biostratigraphy and Geochronology (ed. Woodburne M. O.), pp. 106–155. Columbia University Press, New York.

[brv12242-bib-0235] Rook, D. L. & Hunter, J. P. (2014). Rooting around the eutherian family tree: the origin and relations of the Taeniodonta. Journal of Mammalian Evolution 21, 75–91.

[brv12242-bib-0236] Rose, K. D. (1981). The Clarkforkian Land Mammal Age and mammalian faunal composition across the Paleocene‐Eocene boundary. Museum of Paleontology Papers on Paleontology 26, 1–197.

[brv12242-bib-0237] *Rose, K. D. (1982a). Anterior dentition of the early Eocene plagiomenid dermopteran *Worlandia* . Journal of Mammalogy 63, 179–183.

[brv12242-bib-0238] Rose, K. D. (1982b). Skeleton of *Diacodexis*, oldest known artiodactyl. Science 216, 621–623. 17783306 10.1126/science.216.4546.621

[brv12242-bib-0239] Rose, K. D. (1987). Climbing adaptations in the early Eocene mammal *Chriacus* and the origin of Artiodactyla. Science 236, 314–316. 3426662 10.1126/science.3426662

[brv12242-bib-0240] Rose, K. D. (1996). On the origin of the order Artiodactyla. Proceedings of the National Academy of Sciences of the United States of America 93, 1705–1709. 11607634 10.1073/pnas.93.4.1705 PMC40006

[brv12242-bib-0241] Rose, K. D. (1999a). *Eurotamandua* and Palaeanodonta: convergent or related? Palaeontologische Zeitschrift 73, 395–401.

[brv12242-bib-0242] Rose, K. D. (1999b). Postcranial skeleton of Eocene Leptictidae (mammalia), and its implications for behavior and relationships. Journal of Vertebrate Paleontology 19, 355–372.

[brv12242-bib-0243] Rose, K. D. (2006). The Beginning of the Age of Mammals. The John Hopkins University Press, Baltimore.

[brv12242-bib-0244] *Rose, K. D. , Chew, A. E. , Dunn, R. H. , Kraus, M. J. , Fricke, H. C. & Zack, S. P. (2012). Earliest Eocene mammalian fauna from the Paleocene‐Eocene Thermal Maximum at Sand Creek Divide, Southern Bighorn Basin, Wyoming. University of Michigan Papers on Paleontology 36, 1–122.

[brv12242-bib-0245] Rose, K. D. , Emry, R. J. , Gaudin, T. J. & Storch, G. (2005). Xenarthra and Pholidota In The Rise of Placental Mammals: Origins and Relationships of the Major Extant Clades (eds Rose K. D. and Archibald J. D.), pp. 106–126. John Hopkins University Press, Baltimore.

[brv12242-bib-0246] Rose, K. D. , Holbrook, L. T. , Rana, R. S. , Kumar, K. , Jones, K. E. , Ahrens, H. E. , Missiaen, P. , Sahni, A. & Smith, T. (2014). Early Eocene fossils suggest that the mammalian order Perissodactyla originated in India. Nature Communications 5, 5570. 10.1038/ncomms6570 25410701

[brv12242-bib-0247] *Rose, K. D. & Krause, D. W. (1982). Cyriacotheriidae, a new family of early Tertiary Pantodonts from Western North America. Proceedings of the American Philosophical Society 126, 26–50.

[brv12242-bib-0248] *Rose, K. D. & Lucas, S. G. (2000). An early Paleocene Palaeanodont (Mammalia, ?Pholidota) from New Mexico, and the origin of Palaeanodonta. Journal of Vertebrate Paleontology 20, 139–156.

[brv12242-bib-0249] *Rose, K. D. & Walker, A. (1985). The skeleton of early Eocene *Cantius*, oldest lemuriform primate. American Journal of Physical Anthropology 66, 73–89. 3976872 10.1002/ajpa.1330660107

[brv12242-bib-0250] *Rougier, G. W. , Wible, J. R. & Novacek, M. J. (1998). Implications of *Deltatheridium* specimens for early marsupial history. Nature 396, 459–463. 9853752 10.1038/24856

[brv12242-bib-0251] Russell, D. E. (1964). The Paleocene mammals of Europe. Mémoires du Musée National d'Histoire Naturelle Série C 13, 1–324.

[brv12242-bib-0252] *Sánchez‐Villagra, M. R. , Horovitz, I. & Motokawa, M. (2006). A comprehensive morphological analysis of talpid moles (Mammalia) phylogenetic relationships. Cladistics 22, 59–88. 10.1111/j.1096-0031.2006.00087.x 34892894

[brv12242-bib-0253] Sansom, R. S . (2014). Dental morphology of mammals is less reliable than osteology: phylogenetic differences align with taphonomic biases. In 62nd Symposium for Vertebrate Palaeontology and Comparative Anatomy, York, United Kingdom.

[brv12242-bib-0254] Schaeffer, B. (1947). Notes on the origin and function of the artiodactyl tarsus. American Museum Novitates 1356, 1–24.

[brv12242-bib-0255] *Scott, W. B. (1891). On the osteology of *Pœbrotherium*: a contribution to the phylogeny of the Tylopoda. Journal of Morphology 5, 1–78.

[brv12242-bib-0256] Scott, C. S. (2010). New cyriacotheriid pantodonts (Mammalia, Pantodonta) from the Paleocene of Alberta, Canada, and the Relationships of Cyriacotheriidae. Journal of Paleontology 84, 197–215.

[brv12242-bib-0257] Scott, C. S. , Spivak, D. N. & Sweet, A. R. (2013). First mammals from the Paleocene Porcupine Hills Formation of southwestern Alberta, Canada. Canadian Journal of Earth Sciences 50, 355–378.

[brv12242-bib-0258] *Seiffert, E. R. (2010). The oldest and youngest records of afrosoricid placentals from the Fayum Depression of Northern Egypt. Acta Palaeontologica Polonica 55, 599–616.

[brv12242-bib-0259] *Seiffert, E. R. & Simons, E. L. (2000). *Widanelfarasia*, a diminutive placentalfrom the Late Eocene of Egypt. Proceedings of the National Academy of Sciences of the United States of America 97, 2646–2651. 10694573 10.1073/pnas.040549797 PMC15983

[brv12242-bib-0260] *Seiffert, E. R. , Simons, E. L. , Ryan, T. M. , Bown, T. M. & Attia, Y. (2007). New remains of Eocene and Oligocene Afrosoricida (Afrotheria) from Egypt, with implications for the origin of afrosoricid zalambdodonty. Journal of Vertebrate Paleontology 27, 963–972.

[brv12242-bib-0261] Sessa, J. A. , Bralower, T. J. , Patzkowsky, M. E. , Handley, J. C. & Ivany, L. C. (2012). Environmental and biological controls on the diversity and ecology of Late Cretaceous through early Paleogene marine ecosystems in the U.S. Gulf Coastal Plain. Paleobiology 38, 218–239.

[brv12242-bib-0262] *Silcox, M.T. (2001). A phylogenetic analysis of Plesiadapiformes and their relationship to Euprimates and other archontans. PhD Thesis: John Hopkins University.

[brv12242-bib-0263] Simmons, N. B. , Seymour, K. L. , Habersetzer, J. & Gunnell, G. F. (2008). Primitive early Eocene bat from Wyoming and the evolution of flight and echolocation. Nature 451, 818–822. 18270539 10.1038/nature06549

[brv12242-bib-0264] Simons, E. L. (1960). The Paleocene Pantodonta. Transactions of the American Philosophical Society 50, 1–81.

[brv12242-bib-0265] Simpson, G. G. (1937). The Fort Union of the Crazy Mountain field, Montana, and its mammalian faunas. Bulletin of the United States National Museum 169, 1–287.

[brv12242-bib-0266] Simpson, G. G. (1945). The principles of classification and a classification of mammals. Bulletin of the American Museum of Natural History 85, 1–350.

[brv12242-bib-0267] Simpson, G. G. (1953). The Major Features of Evolution. Columbia University Press, New York.

[brv12242-bib-0268] *Simpson, G. G. (1957). Fossil mammals from the type area of the Puerco and Nacimiento Strata, Paleocene of New Mexico. American Museum Novitates 1957, 1–22.

[brv12242-bib-0269] Slater, G. J. (2013). Phylogenetic evidence for a shift in the mode of mammalian body size evolution at the Cretaceous‐Palaeogene boundary. Methods in Ecology and Evolution 4, 734–744.

[brv12242-bib-0270] Slater, G. J. , Harmon, L. J. & Alfaro, M. E. (2012). Integrating fossils with molecular phylogenies improves inference of trait evolution. Evolution 66, 3931–3944. 23206147 10.1111/j.1558-5646.2012.01723.x

[brv12242-bib-0271] Sloan, R. E. & Van Valen, L. (1965). Cretaceous mammals from Montana. Science 148, 220. 17780082 10.1126/science.148.3667.220

[brv12242-bib-0272] Smith, T. , De Bast, E. & Sige, B. (2010). Euarchontan affinity of Paleocene Afro‐European adapisoriculid mammals and their origin in the late Cretaceous Deccan Traps of India. Naturwissenschaften 97, 417–422. 20174778 10.1007/s00114-010-0651-5

[brv12242-bib-0273] Smith, T. & Smith, R. (2001). The creodonts (Mammalia, Ferae) from the Paleocene‐Eocene transition in Belgium (Tienen Formation, MP7). Belgian Journal of Zoology 131, 117–135.

[brv12242-bib-0274] Smith, T. & Smith, R. (2003). Terrestrial mammals as biostratigraphic indicators in Upper Paleocene‐Lower Eocene marine deposits of the southern North Sea Basin. Geological Society of America, Special Paper 369, 513–520.

[brv12242-bib-0275] Smith, R. , Smith, T. & Sudre, J. (1996). *Diacodexis gigasei* n. sp., the oldest Belgian artiodactyl (Mammalia), found near the Palaeocene‐Eocene transition. Bulletin de l'Institut Royal des Sciences Naturelles de Belgique: Sciences de la Terre 66, 177–196.

[brv12242-bib-0276] Sole, F. , Gheerbrant, E. , Amaghzaz, M. & Bouya, B. (2009). Further evidence of the African antiquity of hyaenodontid ('Creodonta', Mammalia) evolution. Zoological Journal of the Linnean Society 156, 827–846.

[brv12242-bib-0277] Sole, F. & Smith, T. (2013). Dispersals of placental carnivorous mammals (Carnivoramorpha, Oxyaenodonta & Hyaenodontida) near the Paleocene‐Eocene boundary: a climatic and almost worldwide story. Geologica Belgica 16, 254–261.

[brv12242-bib-0278] Song, S. , Liu, L. , Edwards, S. V. & Wu, S. (2012). Resolving conflict in eutherian mammal phylogeny using phylogenomics and the multispecies coalescent model. Proceedings of the National Academy of Sciences of the United States of America 109, 14942–14947. 22930817 10.1073/pnas.1211733109 PMC3443116

[brv12242-bib-0279] Spaulding, M. , O'Leary, M. A. & Gatesy, J. (2009). Relationships of Cetacea (Artiodactyla) among mammals: increased taxon sampling alters interpretations of key fossils and character evolution. PLoS One 4, 1–14. 10.1371/journal.pone.0007062 PMC2740860 19774069

[brv12242-bib-0280] Springer, M. S. , Murphy, W. J. , Eizirik, E. & O'Brien, S. J. (2003). Placental mammal diversification and the Cretaceous‐Tertiary boundary. Proceedings of the National Academy of Sciences of the United States of America 100, 1056–1061. 12552136 10.1073/pnas.0334222100 PMC298725

[brv12242-bib-0281] Springer, M. S. , Stanhope, M. J. , Madsen, O. & de Jong, W. W. (2004). Molecules consolidate the placental mammal tree. Trends in Ecology & Evolution 19, 430–438. 16701301 10.1016/j.tree.2004.05.006

[brv12242-bib-0282] Stanhope, M. J. , Waddell, V. G. , Madsen, O. , de Jong, W. , Hedges, S. B. , Cleven, G. C. , Kao, D. & Springer, M. S. (1998). Molecular evidence for multiple origins of Insectivora and for a new order of endemic African insectivore mammals. Proceedings of the National Academy of Sciences of the United States of America 95, 9967–9972. 9707584 10.1073/pnas.95.17.9967 PMC21445

[brv12242-bib-0283] *St. Clair, E. M. , Boyer, D. M. , Bloch, J. I. & Krause, D. W. (2010). First records of a triisodontine mammal (*Goniacodon levisanus*) in the late Paleocene of the Northern Great Plains, North America. Journal of Vertebrate Paleontology 30, 604–608.

[brv12242-bib-0284] *Stock, C. (1934). Microsyopinæ and Hyopsodontinæ in the Sespe Upper Eocene, California. Geology 20, 349–354. 10.1073/pnas.20.6.349 PMC1076417 16587901

[brv12242-bib-0285] Storch, G. (1978). Messel fossil finds part 14: *Eomanis waldi* (new genus, new species), a pangolin from the middle Eocene of the messel pit near darmstadt, West Germany (Mammalia, Pholidota). Senckenbergiana Lethaea 59, 503–530.

[brv12242-bib-0286] Stucky, R. K. & Hardy, T. G. (2007). A new large, hypercarnivorous oxyaenid (Mammalia, Creodonta) from the middle Eocene of the Wind River Formation, Natrona County, Wyoming. Bulletin of Carnegie Museum of Natural History 39, 57–65.

[brv12242-bib-0287] *Szalay, F. S. (1969). Mixodectidae, Microsyopidae, and the insectivore‐primate transition. Bulletin of the American Museum of Natural History 140, 195–330.

[brv12242-bib-0288] Szalay, F. S. & Decker, R. L. (1974). Origins, evolution and function of the tarsus in Late Cretaceous Eutheria and Paleocene primates In Primate Locomotion (ed. Jenkins F. A.), pp. 223–259. Illustrated Academic Press, New York.

[brv12242-bib-0289] *Tabuce, R. , Antunes, M. T. , Smith, R. & Smith, T. (2006). Dental and tarsal morphology of the European Palaeocene/Eocene “condylarth” mammal *Microhyus* . Acta Palaeontologica Polonica 51, 37–52.

[brv12242-bib-0290] Tabuce, R. , Asher, R. J. & Lehmann, T. (2008). Afrotherian mammals: a review of current data. Mammalia 72, 2–14.

[brv12242-bib-0291] Tabuce, R. , Clavel, J. & Antunes, M. T. (2011). A structural intermediate between triisodontids and mesonychians (Mammalia, Acreodi) from the earliest Eocene of Portugal. Naturwissenschaften 98, 145–155. 21181109 10.1007/s00114-010-0747-y

[brv12242-bib-0292] Tabuce, R. , Coiffait, B. , Coiffait, P. E. , Mahboubi, M. & Jaeger, J. J. (2001). A new genus of Macroscelidea (Mammalia) from the Eocene of Algeria: a possible origin for elephant‐shrews. Journal of Vertebrate Paleontology 21, 535–546.

[brv12242-bib-0293] *Tabuce, R. , Marivaux, L. , Adaci, M. , Bensalah, M. , Hartenberger, J.‐L. , Mahboubi, M. , Mebrouk, F. , Tafforeau, P. & Jaeger, J.‐J. (2007). Early Tertiary mammals from North Africa reinforce the molecular Afrotheria clade. Proceedings of the Royal Society B: Biological Science 274, 1159–1166. 10.1098/rspb.2006.0229 PMC2189562 17329227

[brv12242-bib-0294] Tarver, J. E. & Donoghue, P. C. J. (2011). The trouble with topology: phylogenies without fossils provide a revisionist perspective of evolutionary history in topological analyses of diversity. Systematic Biology 60, 700–712. 21436106 10.1093/sysbio/syr018

[brv12242-bib-0295] Teeling, E. C. & Hedges, S. B. (2013). Making the impossible possible: rooting the tree of placental mammals. Molecular Biology and Evolution 30, 1999–2000. 23813980 10.1093/molbev/mst118

[brv12242-bib-0296] Templeton, A. R. (1983). Phylogenetic inference from restriction endonuclease cleavage site maps with particular reference to the evolution of humans and the apes. Evolution 37, 221–244. 28568373 10.1111/j.1558-5646.1983.tb05533.x

[brv12242-bib-0297] Theodor, J. M. & Foss, S. E. (2005). Deciduous dentitions of Eocene cebochoerid artiodactyls and cetartiodactyl relationships. Journal of Mammalian Evolution 12, 161–181.

[brv12242-bib-0298] Thewissen, J. G. M. (1990). Evolution of Paleocene and Eocene Phenacodontidae (Mammalia, Condylarthra). Museum of Paleontology Papers on Paleontology III‐VIII, 1–107.

[brv12242-bib-0299] *Thewissen, J. G. M. (1991). Limb osteology and function of the primitive Paleocene ungulate *Pleuraspidotherium* with notes on *Tricuspiodon* and *Dissacus* (Mammalia). Geobios 24, 483–495.

[brv12242-bib-0300] *Thewissen, J. G. M. (1994). Phylogenetic aspects of cetacean origins: a morphological perspective. Journal of Mammalian Evolution 2, 157–183.

[brv12242-bib-0301] *Thewissen, J. G. M. , Cooper, L. N. , Clementz, M. T. , Bajpai, S. & Tiwari, B. N. (2007). Whales originated from aquatic artiodactyls in the Eocene epoch of India. Nature 450, 1190–1194. 18097400 10.1038/nature06343

[brv12242-bib-0302] *Thewissen, J. G. M. & Domning, D. G. (1992). The role of phenacodontids in the origin of the modern orders of ungulate mammals. Journal of Vertebrate Paleontology 12, 494–504.

[brv12242-bib-0303] *Thewissen, J. G. M. & Gingerich, P. D. (1989). Skull and endocranial cast of *Eoryctes melanus*, a new palaeoryctid (Mammalia, Insectivora) from the early Eocene of Western North America. Journal of Vertebrate Paleontology 9, 459–470.

[brv12242-bib-0304] Thewissen, J. G. M. , Williams, E. M. , Roe, L. J. & Hussain, S. T. (2001). Skeletons of terrestrial cetaceans and the relationship of whales to artiodactyls. Nature 413, 277–281. 11565023 10.1038/35095005

[brv12242-bib-0305] Ting, S. Y. , Bowen, G. J. , Koch, P. L. , Clyde, W. C. , Wang, Y. , Wang, Y. & McKenna, M. C. (2003). Biostratigraphic, chemostratigraphic, and magnetostratigraphic study across the Paleocene‐Eocene boundary in the Hengyang Basin, Hunan, China. Geological Society of America, Special Paper 369, 521–535.

[brv12242-bib-0306] *Ting, S. Y. & Li, C. K. (1987). The skull of *Hapalodectes* (Acreodi, Mammalia), with notes on some Chinese Paleocene mesonychids. Vertebrata PalAsiatica 25, 161–186.

[brv12242-bib-0307] Tomiya, S. (2011). A new basal caniform (Mammalia: Carnivora) from the middle Eocene of North America and Remarks on the Phylogeny of early Carnivorans. PLoS One 6(9), e24146. 21935380 10.1371/journal.pone.0024146 PMC3173397

[brv12242-bib-0308] Ungar, P. S. (2010). Mammal Teeth: Origin, Evolution, and Diversity. John Hopkins University Press, Baltimore.

[brv12242-bib-0309] Vajda, V. , Raine, J. I. & Hollis, C. J. (2001). Indication of global deforestation at the Creataceous‐Tertiary boundary by New Zealand fern spike. Science 294, 1700–1702. 11721051 10.1126/science.1064706

[brv12242-bib-0310] Van Valen, L. (1969). The multiple origins of the placental carnivores. Evolution 23, 118–130. 28562968 10.1111/j.1558-5646.1969.tb03499.x

[brv12242-bib-0311] Van Valen, L. (1978). The beginning of the age of Mammals. Evolutionary Theory 4, 46–80.

[brv12242-bib-0312] Van Valen, L. & Sloan, R. E. (1965). The earliest Primates. Science 150, 743. 5891702 10.1126/science.150.3697.743

[brv12242-bib-0313] Van Valkenburgh, B. (1999). Major patterns in the history of carnivorous mammals. Annual Review of Earth and Planetary Sciences 27, 463–493.

[brv12242-bib-0314] Venditti, C. , Meade, A. & Pagel, M. (2011). Multiple routes to mammalian diversity. Nature 479, 393–396. 22012260 10.1038/nature10516

[brv12242-bib-0315] Waddell, P. J. , Cao, Y. , Hauf, J. & Hasegawa, M. (1999). Using novel phylogenetic methods to evaluate mammalian mtDNA, including amino acid invariant sites LogDet plus site stripping, to detect internal conflicts in the data, with special reference to the positions of hedgehog, armadillo, and elephant. Systematic Biology 48, 31–53. 12078643 10.1080/106351599260427

[brv12242-bib-0316] Waddell, P. J. , Kishino, H. & Ota, R. (2001). A phylogenetic foundation for comparative mammalian genomics. Genome Informatics International Conference on Genome Informatics 12, 141–154. 11791233

[brv12242-bib-0317] *Webb, S. D. & Taylor, B. E. (1980). The phylogeny of hornless ruminants and a description of the cranium of *Archaeomeryx* . Bulletin of the American Museum of Natural History 167, 121–157.

[brv12242-bib-0318] Welker, F. , Collins, M. J. , Thomas, J. A. , Wadsley, M. , Brace, S. , Cappellini, E. , Turvey, S. T. , Reguero, M. , Gelfo, J. N. , Kramarz, A. , Burger, J. , Thomas‐Oates, J. , Ashford, D. A. , Ashton, P. D. , Rowsell, K. , Porter, D. M. , Kessler, B. , Fischer, R. , Baessmann, C. , Kaspar, S. , Olson, J. V. , Kiley, P. , Elliott, J. A. , Kelstrup, C. D. , Mullin, V. , Hofreiter, M. , Willerslev, E. , Hublin, J.‐J. , Orlando, L. , Barnes, I. & MacPhee, R. D. E. (2015). Ancient proteins resolve the evolutionary history of Darwin's South American ungulates. Nature 522, 81–84. 25799987 10.1038/nature14249

[brv12242-bib-0319] Wesley‐Hunt, G. D. (2005). The morphological diversification of carnivores in North America. Paleobiology 31, 35–55.

[brv12242-bib-0320] *West, R. M. (1970). *Tetraclaenodon puercensis* (Mammalia: Phenacodontidae), Goler Formation, Paleocene of California, and distribution of the genus. Journal of Paleontology 44, 852–857.

[brv12242-bib-0321] *Wible, J. R. , Novacek, M. J. & Rougier, G. W. (2004). New data on the skull and dentition in the Mongolian late Cretaceous Eutherian mammal *Zalambdalestes* . Bulletin of the American Museum of Natural History 281, 1–144.

[brv12242-bib-0322] Wible, J. R. , Rougier, G. W. & Novacek, M. J. (2005). Anatomical evidence for superordinal/ordinal eutherian taxa in the Cretaceous In The Rise of Placental Mammals: Origins and Relationships of the Major Extant Clades (eds Rose K. D. and Archibald J. D.). John Hopkins University Press, Baltimore, 15–36.

[brv12242-bib-0323] Wible, J. R. , Rougier, G. W. , Novacek, M. J. & Asher, R. J. (2007). Cretaceous eutherians and Laurasian origin for placental mammals near the K/T boundary. Nature 447, 1003–1006. 17581585 10.1038/nature05854

[brv12242-bib-0324] Wible, J. R. , Rougier, G. W. , Novacek, M. J. & Asher, R. J. (2009). The eutherian mammal *Maelestes gobiensis* from the late cretaceous of mongolia and the phylogeny of Cretaceous Eutheria. Bulletin of the American Museum of Natural History 327, 1–123.

[brv12242-bib-0325] Wiens, J. J. (2001). Character analysis in morphological phylogenetics: problems and solutions. Systematic Biology 50, 689–699. 12116939 10.1080/106351501753328811

[brv12242-bib-0326] Wilkinson, M. (1992). Ordered versus unordered characters. Cladistics‐the International Journal of the Willi Hennig Society 8, 375–385. 10.1111/j.1096-0031.1992.tb00079.x 34929964

[brv12242-bib-0327] Williamson, T. E. & Carr, T. D. (2007). *Bomburia* and *Ellipsodon* (Mammalia : Mioclaenidae) from the early Paleocene of new Mexico. Journal of Paleontology 81, 966–985.

[brv12242-bib-0328] Williamson, T. E. & Lucas, S. G. (1992). *Meniscotherium* (Mammalia: “Condylarthra”) from the Paleocene‐Eocene of Western North America. New Mexico Museum of Natural History Scientific Bulletin 1, 1–75.

[brv12242-bib-0329] Williamson, T. E. & Weil, A. (2011). A new puercan (early Paleocene) hyopsodontid “condylarth” from New Mexico. Acta Palaeontologica Polonica 56, 247–255.

[brv12242-bib-0330] Williamson, T. E. , Weil, A. & Standhardt, B. (2011). Cimolestids (Mammalia) from the early Paleocene (Puercan) of New Mexico. Journal of Vertebrate Paleontology 31, 162–180.

[brv12242-bib-0331] Wilson, G. P. (2014). Mammalian extinction, survival, and recovery dynamics across the Cretaceous‐Palaeogene boundary in north‐eastern Montana, USA. Geological Society of America Special Papers 503, 365–392.

[brv12242-bib-0332] Wood, H. M. , Matzke, N. J. , Gillespie, R. G. & Griswold, C. E. (2013). Treating fossils as terminal taxa in divergence time estimation reveals ancient vicariance patterns in the palpimanoid spiders. Systematic Biology 62, 264–284. 23192838 10.1093/sysbio/sys092

[brv12242-bib-0333] Yongsheng, T. (1988). Fossil tree shrews from the eocene Hetaoyuan Formation of Xichuan, Henan. Vertebrata PalAsiatica 26, 214–220.

[brv12242-bib-0334] Zack, S. P. (2004). An early eocene arctostylopid (Mammalia: Arctostylopida) from the Green River Basin, Wyoming. Journal of Vertebrate Paleontology 24, 498–501.

[brv12242-bib-0335] Zack, S. P. (2009). The phylogeny of eutherian mammals: a new analysis emphasizing dental and postcranial morphology of paleogene taxa. PhD Thesis: John Hopkins University.

[brv12242-bib-0336] Zack, S. P. (2011). New Species of the rare early Eocene creodont *Galecyon* and the radiation of early Hyaenodontidae. Journal of Paleontology 85, 315–336.

[brv12242-bib-0337] Zack, S. P. , Penkrot, T. A. , Bloch, J. I. & Rose, K. D. (2005a). Affinities of 'hyopsodontids' to elephant shrews and a Holarctic origin of Afrotheria. Nature 434, 497–501. 15791254 10.1038/nature03351

[brv12242-bib-0338] Zack, S. P. , Penkrot, T. A. , Krause, D. W. & Maas, M. C. (2005b). A new apheliscine “condylarth” mammal from the late Paleocene of Montana and Alberta and the phylogeny of “hyopsodontids”. Acta Palaeontologica Polonica 50, 809–830.

[brv12242-bib-0339] *Zan, S. , Wood, C. B. , Rougier, G. W. , Jin, L. , Chen, J. & Schaaf, C. R. (2006). A new “middle” Cretaceous zalambdalestid mammal, from a new locality in Jilin Province, Northeastern China. Journal of the Paleontological Society of Korea 22, 153–172.

[brv12242-bib-0340] *Zheng, J. J. (1979). Notoungulata from the Paleocene of Jiangsu, South China In Mesozoic and Cenozoic Red Beds of South China (eds IVPP ), pp. 387–394. Science Press, Marrickville.

[brv12242-bib-0341] Zhou, X. , Xu, S. , Xu, J. , Chen, B. , Zhou, K. & Yang, G. (2012). Phylogenomic analysis resolves the interordinal relationships and rapid diversification of the laurasiatherian mammals. Systematic Biology 61, 150–164. 21900649 10.1093/sysbio/syr089 PMC3243735

[brv12242-bib-0342] *Zhou, X. , Zhai, R. , Gingerich, P. D. & Chen, L. (1995). Skull of a new mesonychid (Mammalia: Mesonychia) from the late Paleocene of China. Journal of Vertebrate Paleontology 15, 387–400.

