# Supplementary figures and images for: Resolving the relationships of Paleocene placental mammals
Source: Biol Rev Camb Philos Soc. 2015 Dec 21;92(1):521–50. doi: 10.1111/brv.12242 (PMC6849585; doi:10.1111/brv.12242)

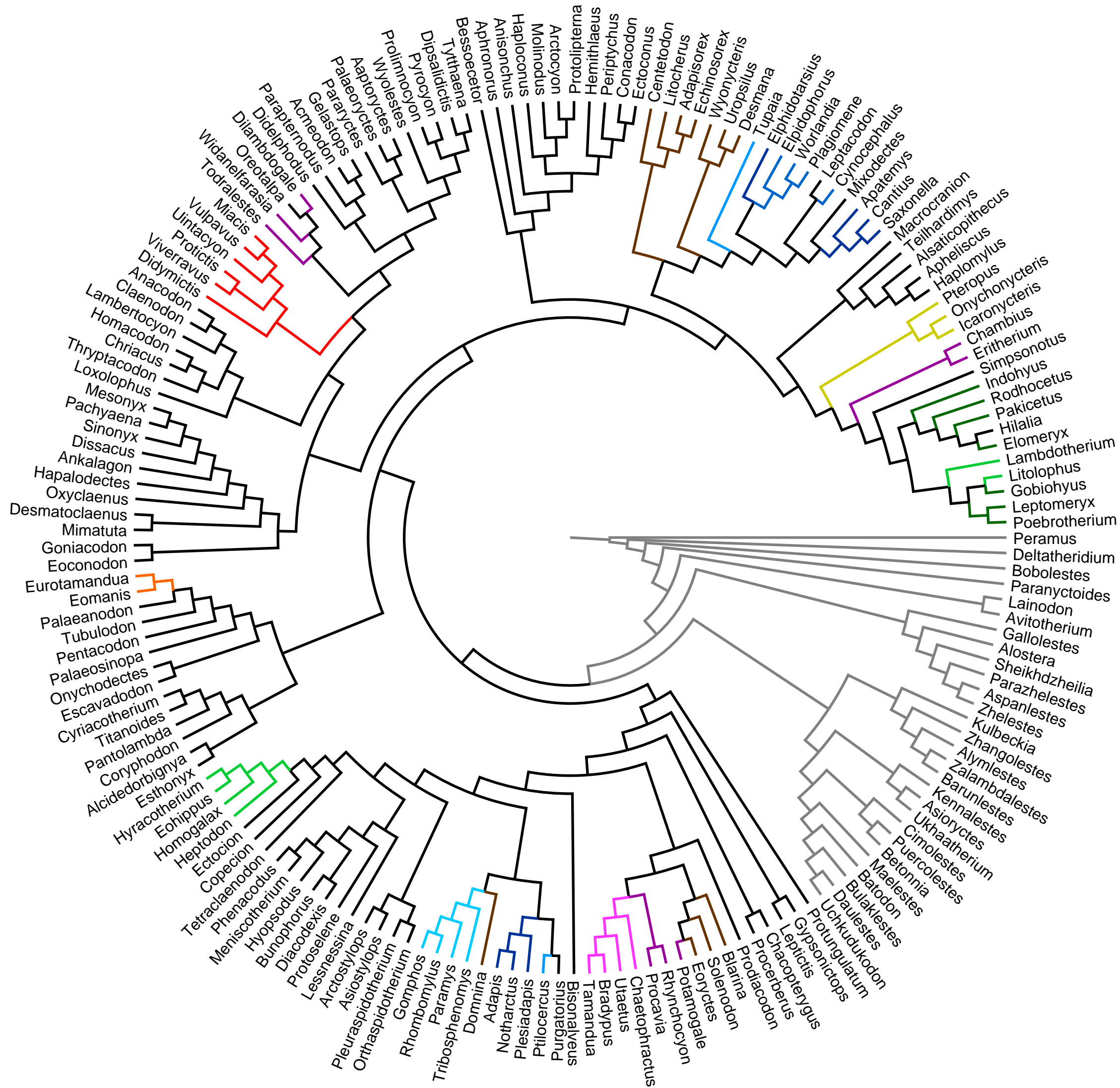

Supplement: Supplementary file 1 — Fig. S1. Consensus topology deriving from the CU analysis. [file BRV-92-521-s005.pdf]

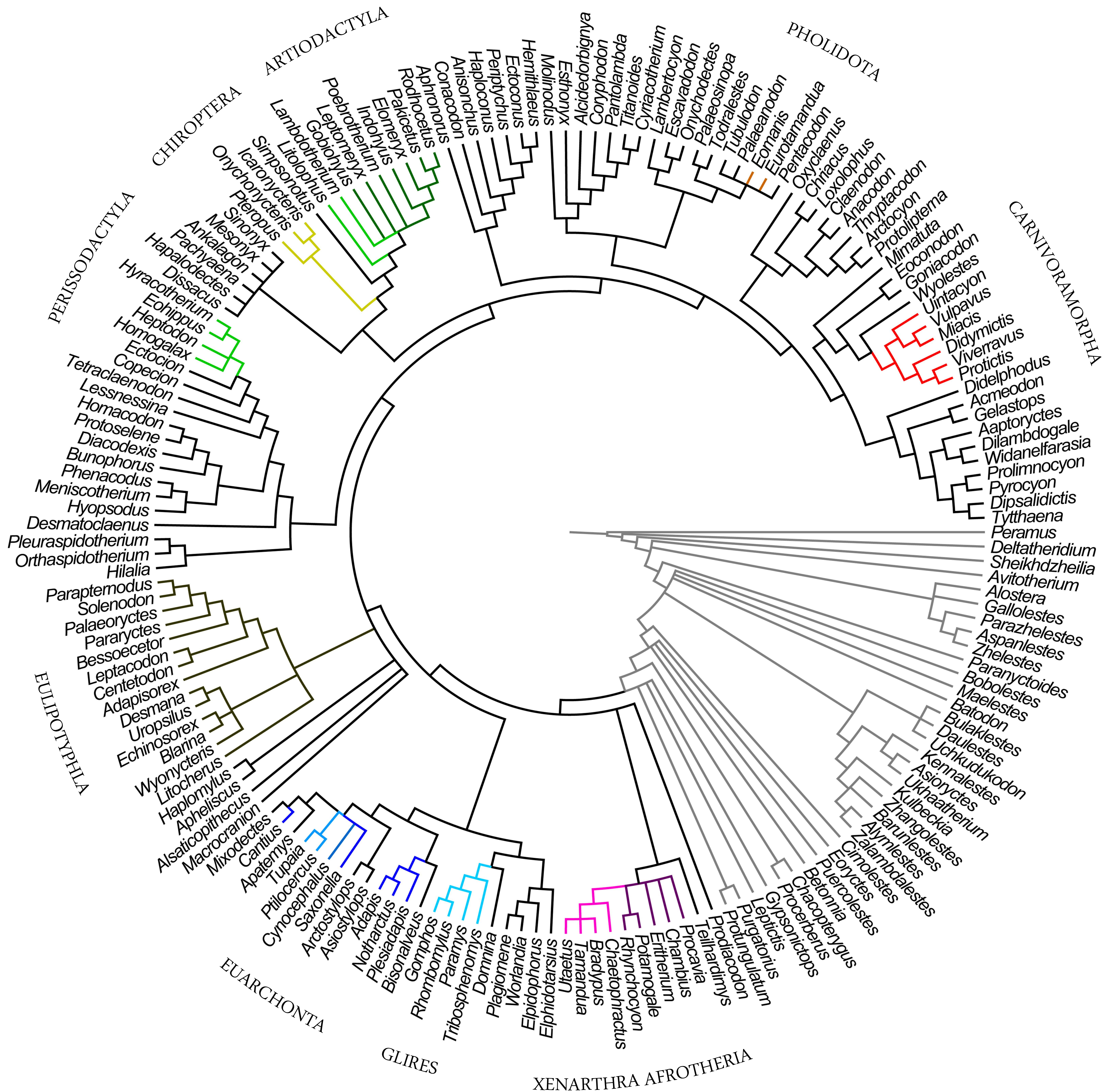

Supplement: Supplementary file 2 — Fig. S2. Consensus topology deriving from the DM analysis. [file BRV-92-521-s008.pdf]

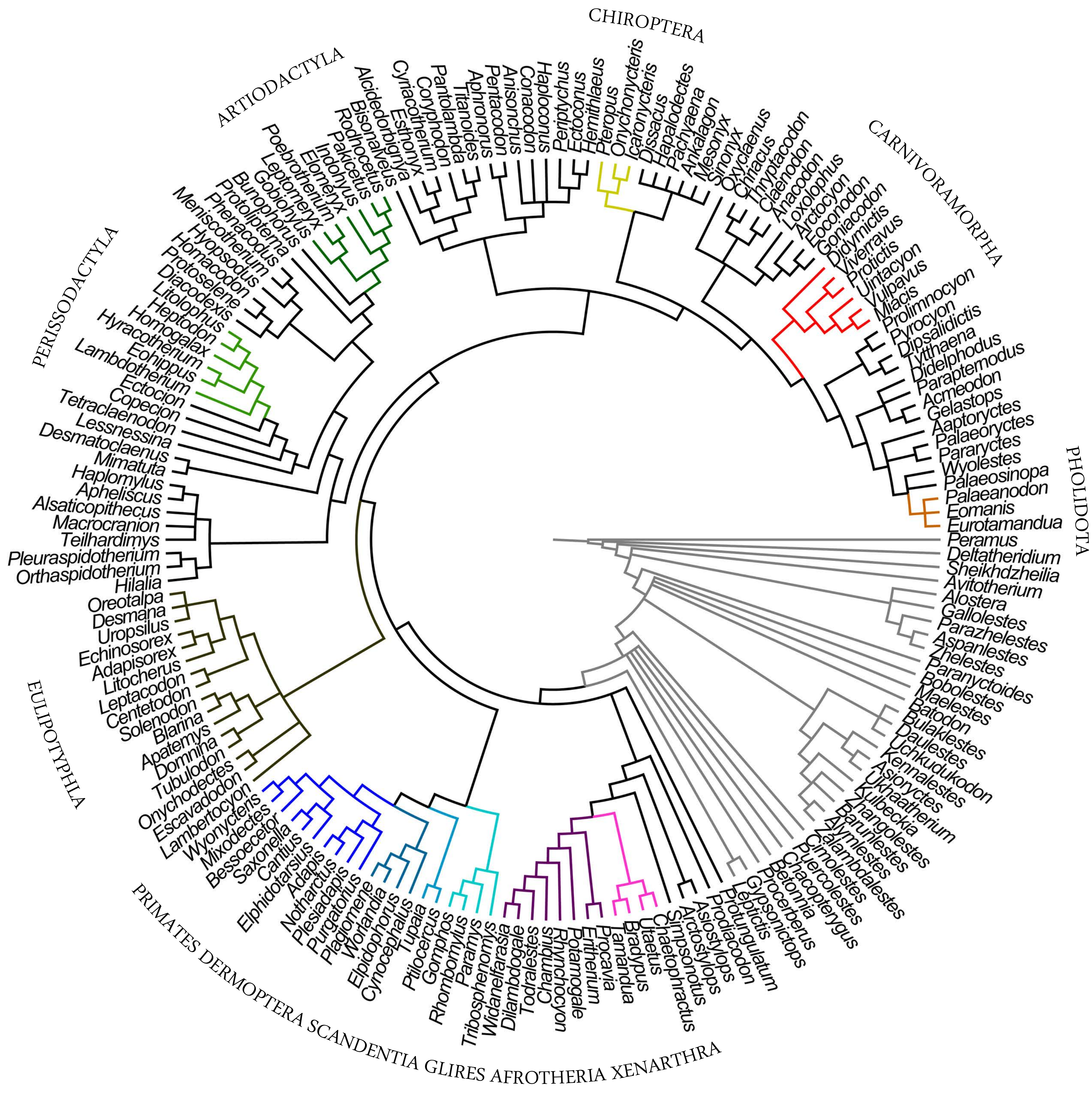

Supplement: Supplementary file 3 — Fig. S3. Consensus topology deriving from the DP analysis. [file BRV-92-521-s012.pdf]

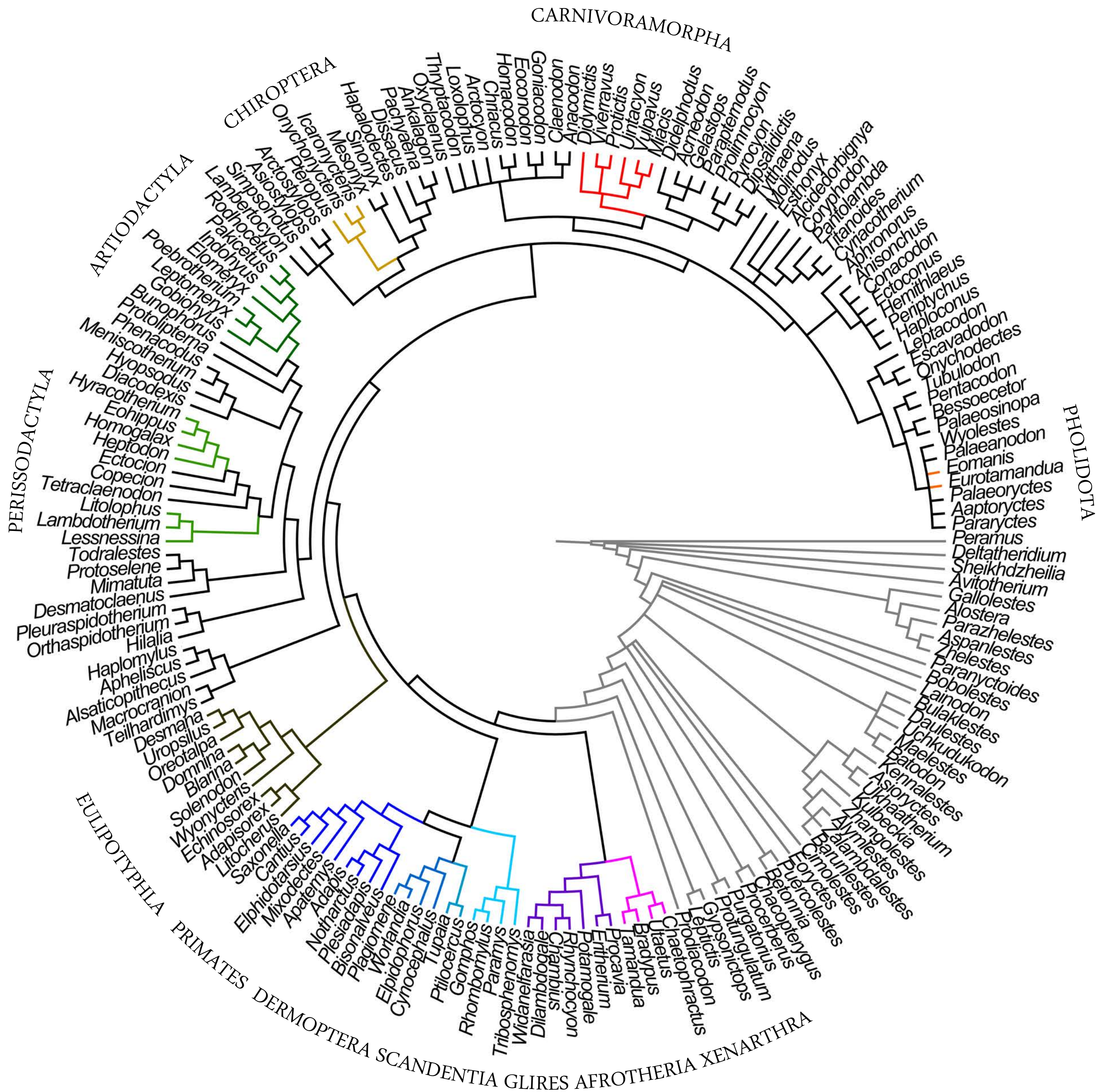

Supplement: Supplementary file 4 — Fig. S4. Consensus topology deriving from the CF analysis. [file BRV-92-521-s018.pdf]

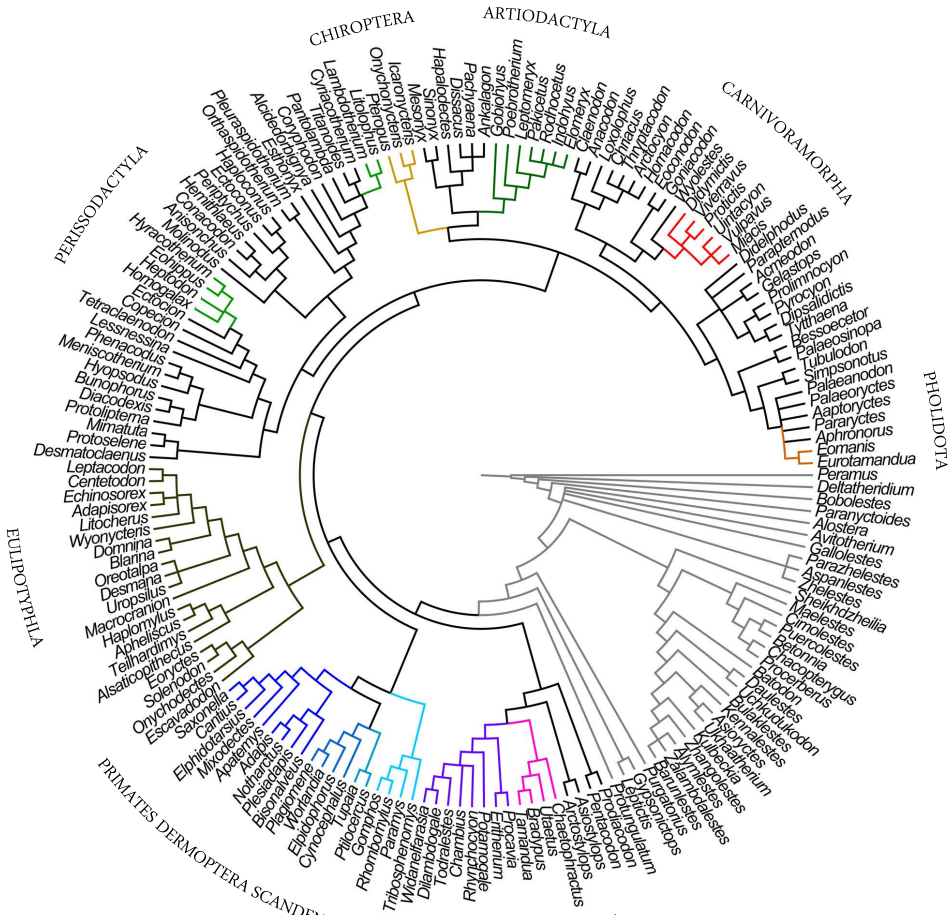

Supplement: Supplementary file 5 — Fig. S5. Consensus topology deriving from the CM analysis. [file BRV-92-521-s016.pdf]

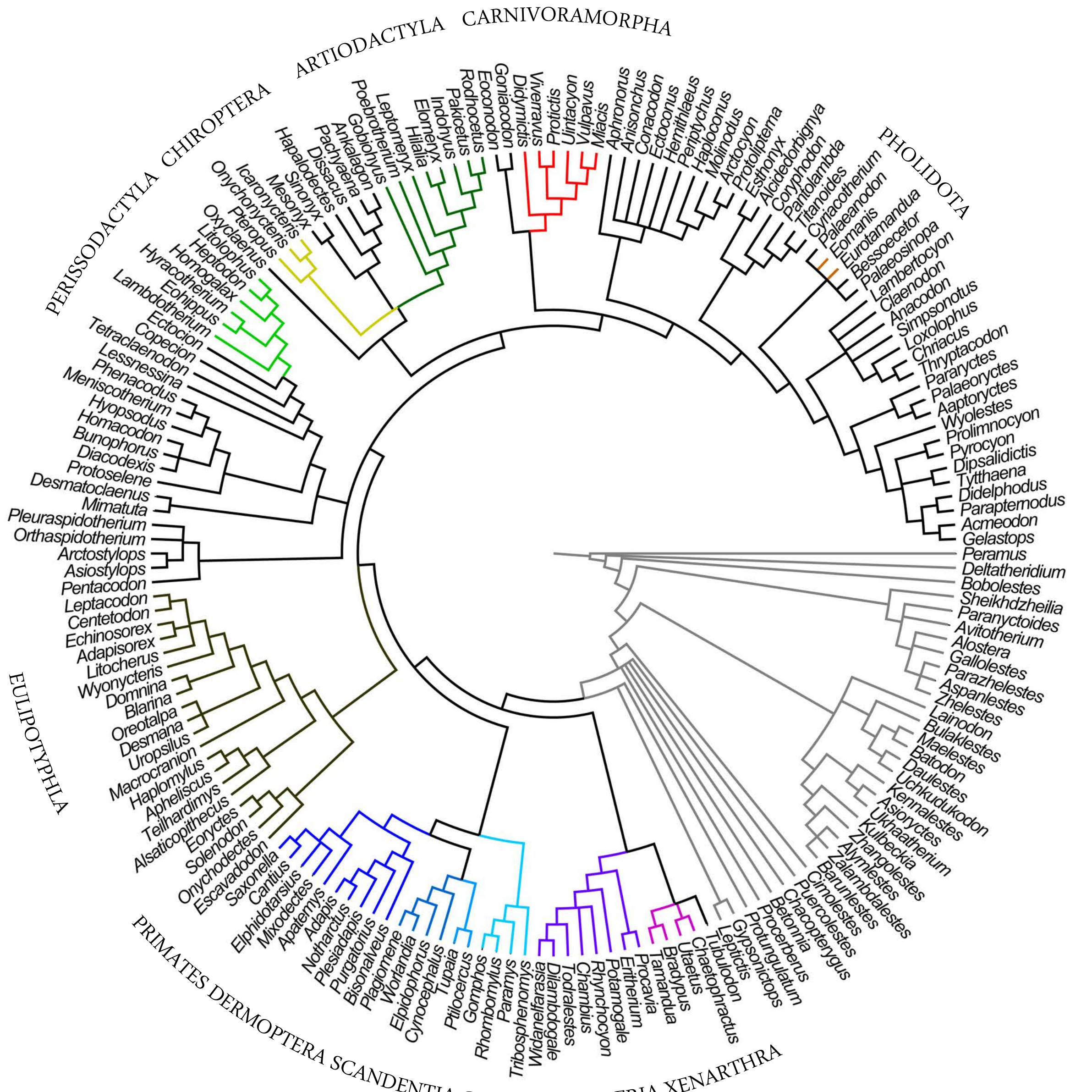

Supplement: Supplementary file 6 — Fig. S6. Consensus topology deriving from the CP analysis. [file BRV-92-521-s010.pdf]

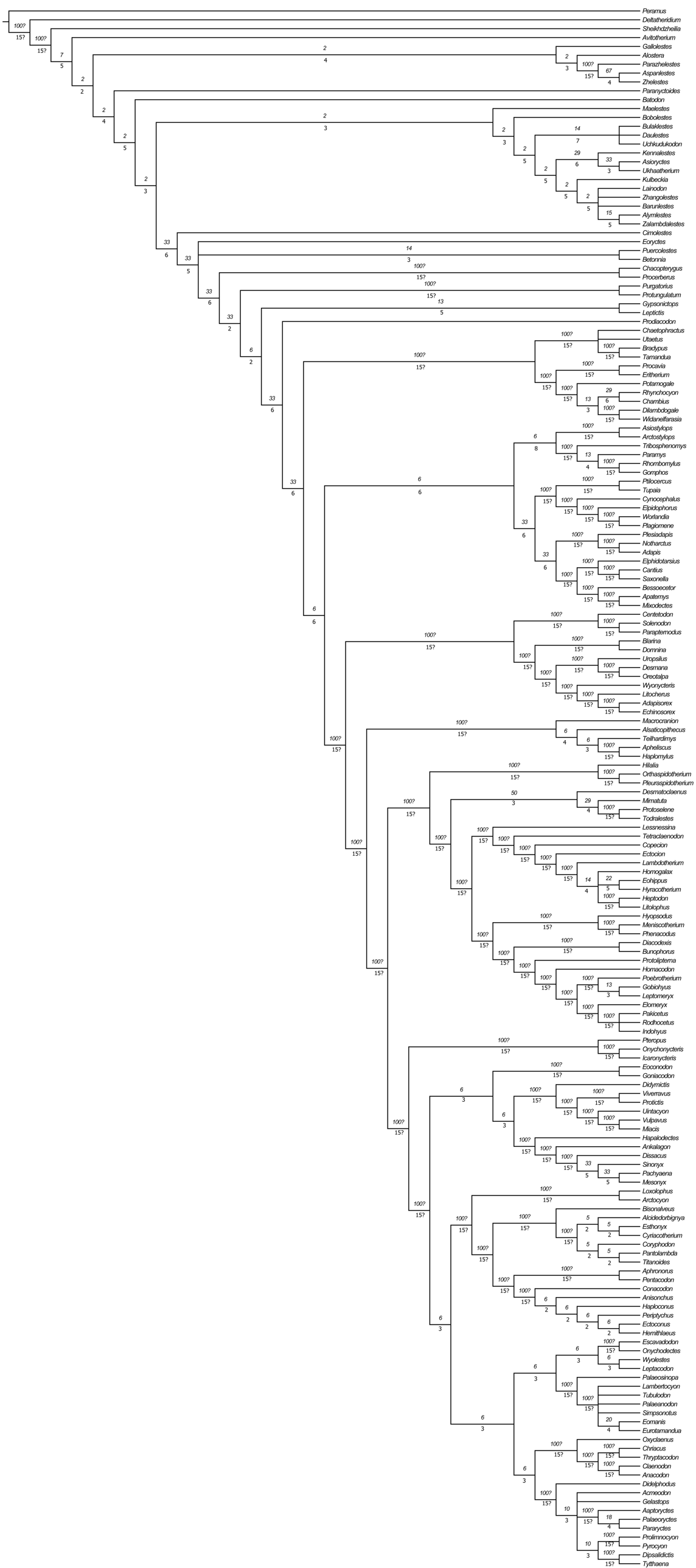

Supplement: Supplementary file 7 — Fig. S7. Bremer support tree from the DF analysis. [file BRV-92-521-s007.pdf]

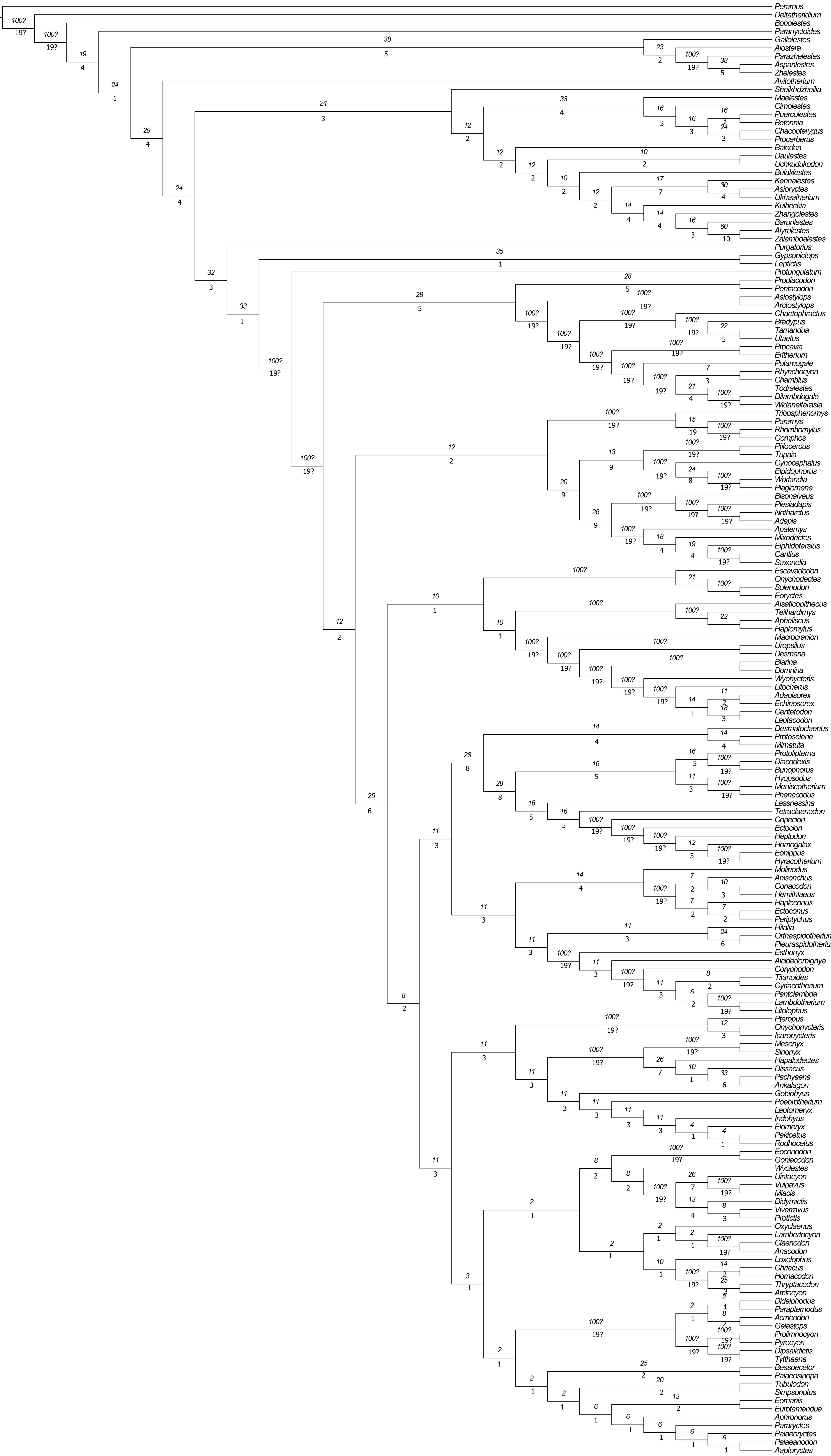

Supplement: Supplementary file 8 — Fig. S8. Bremer support tree from the DM analysis. [file BRV-92-521-s009.pdf]

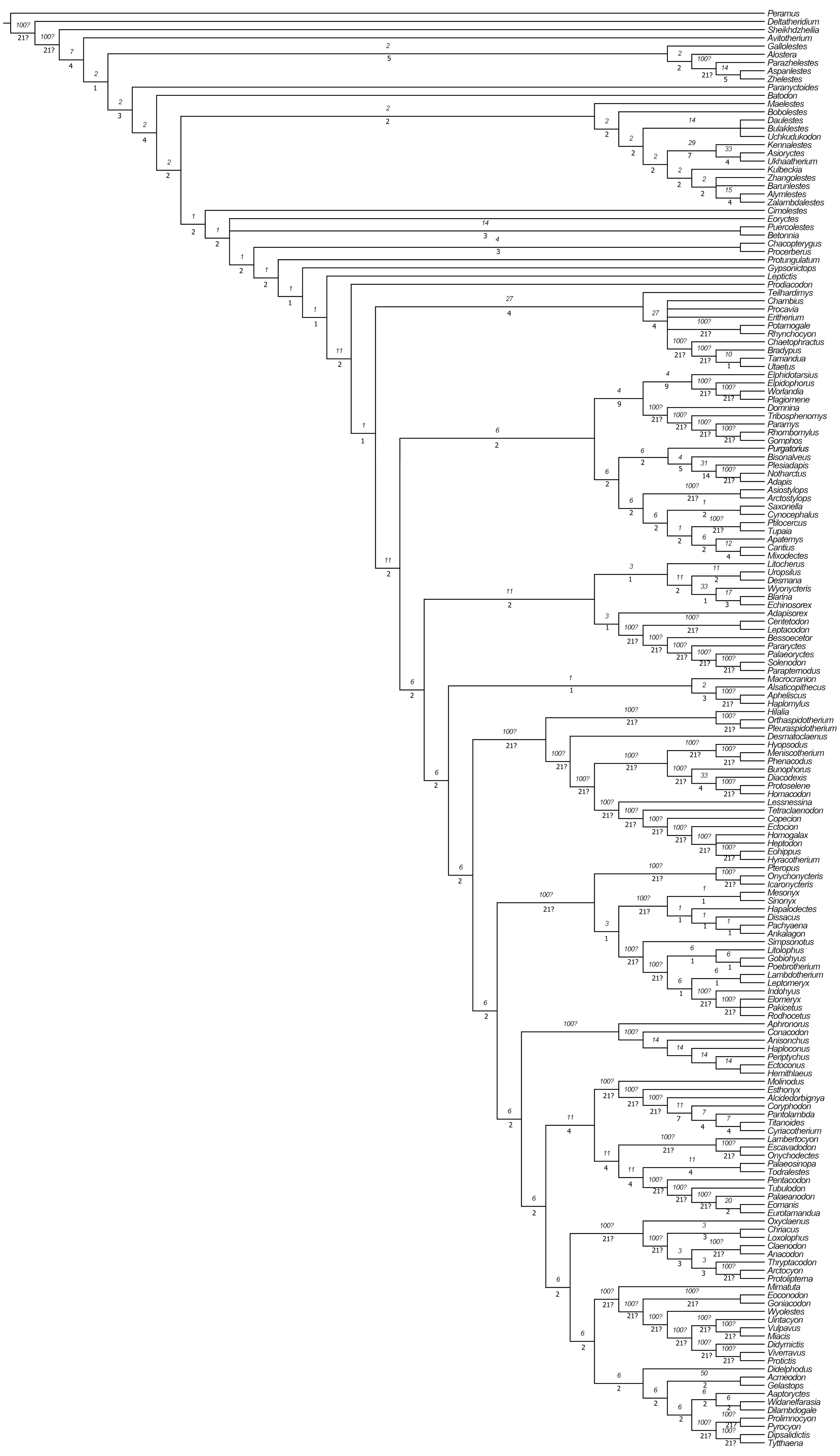

Supplement: Supplementary file 9 — Fig. S9. Bremer support tree from the DP analysis. [file BRV-92-521-s006.pdf]

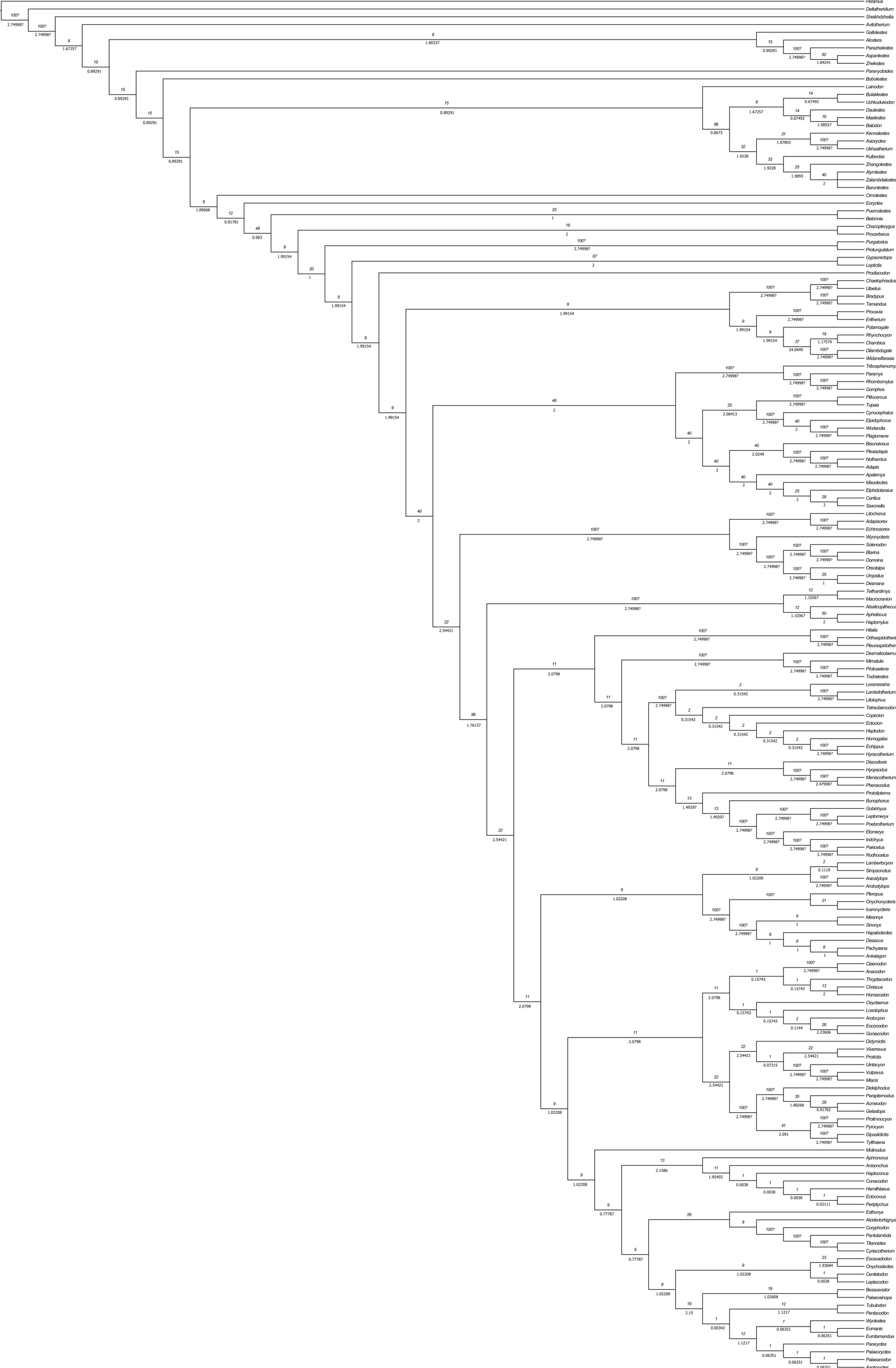

Supplement: Supplementary file 10 — Fig. S10. Bremer support tree from the CF analysis. [file BRV-92-521-s002.pdf]

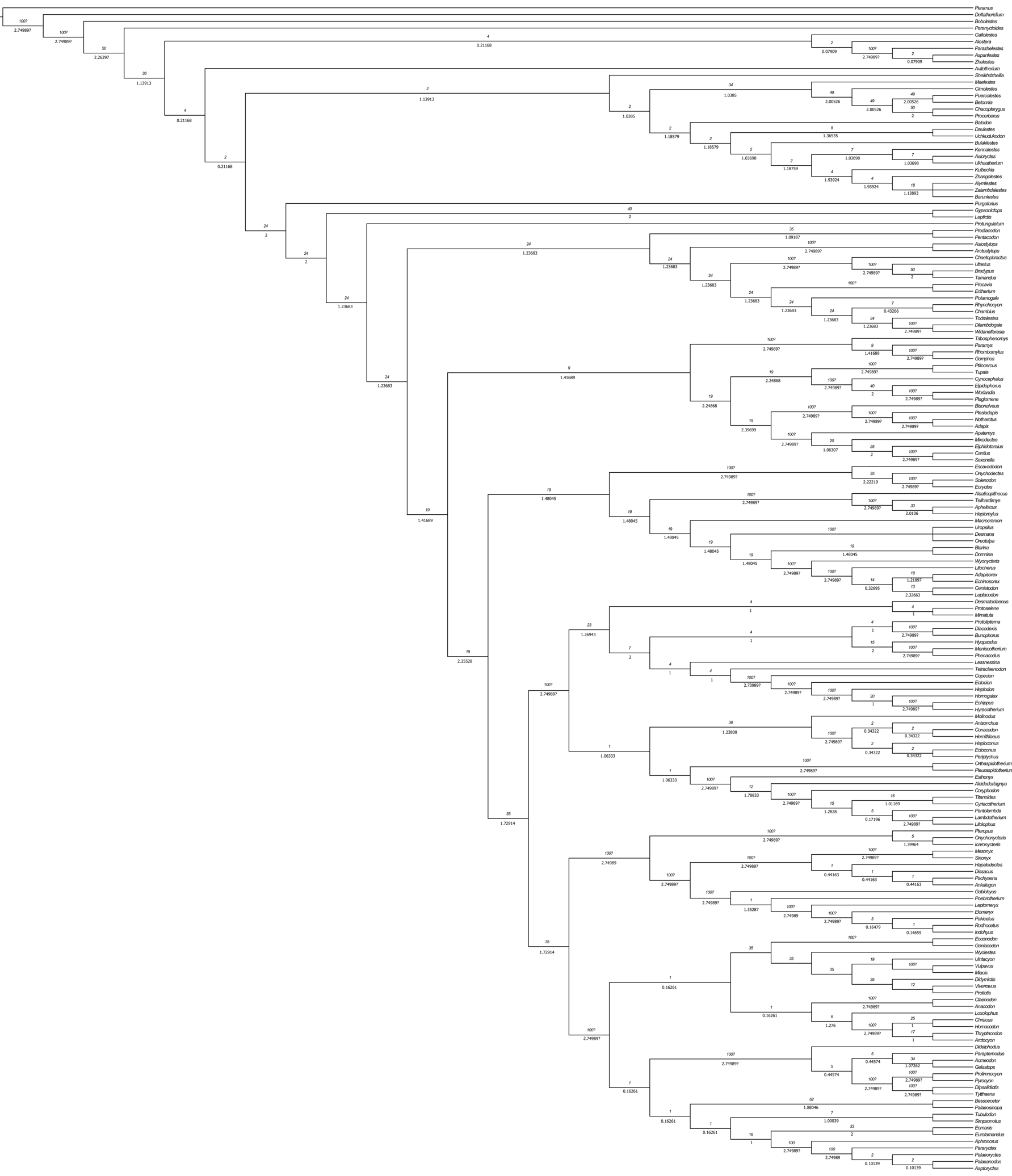

Supplement: Supplementary file 11 — Fig. S11. Bremer support tree from the CM analysis. [file BRV-92-521-s011.pdf]

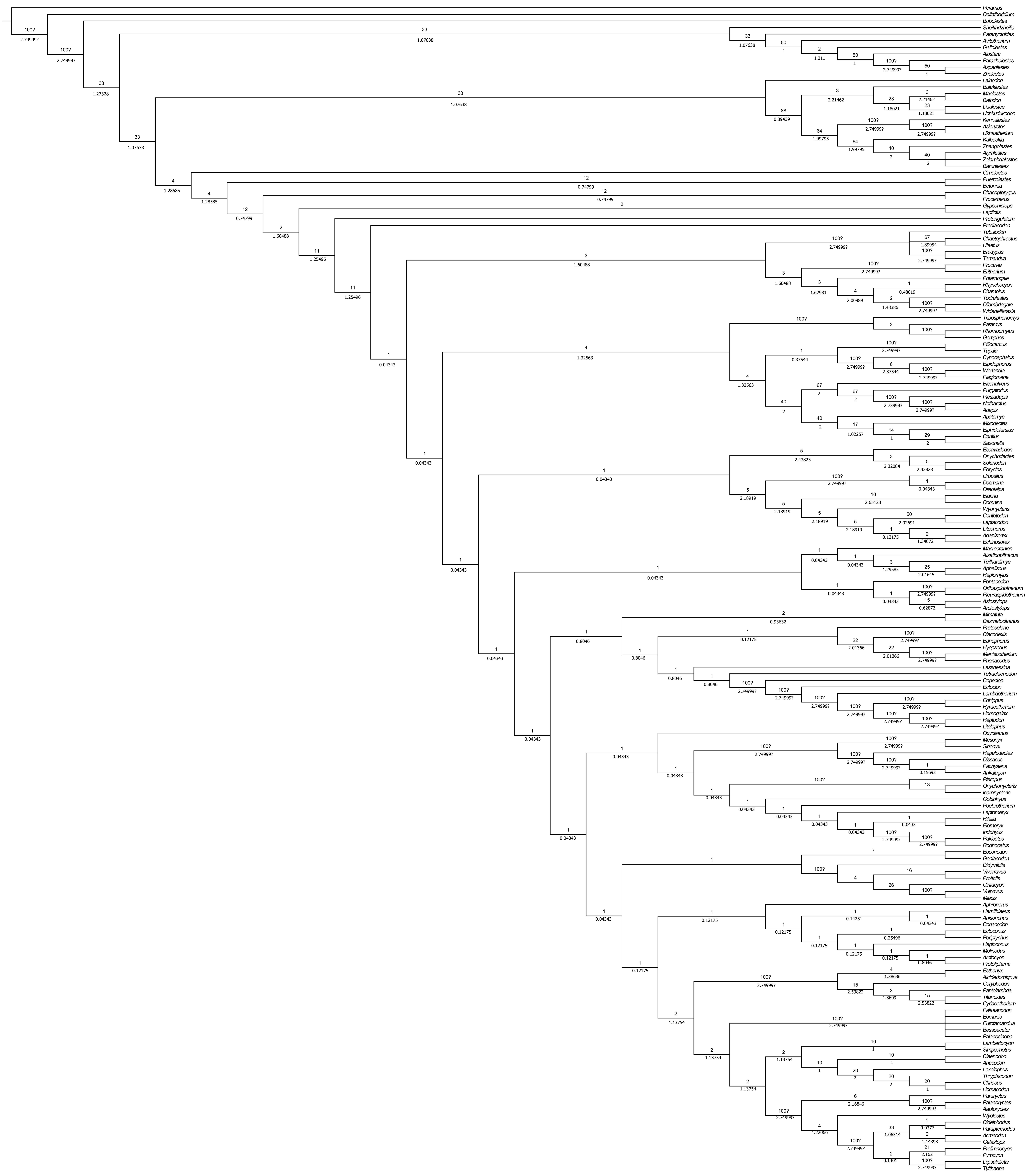

Supplement: Supplementary file 12 — Fig. S12. Bremer support tree from the CP analysis. [file BRV-92-521-s015.pdf]
