# Supplementary material for: Resolving the relationships of Paleocene placental mammals
Source: Biol Rev Camb Philos Soc. 2015 Dec 21;92(1):521–50. doi: 10.1111/brv.12242 (PMC6849585; doi:10.1111/brv.12242)
Supplement: Supplementary file 17 — Appendix S5. Additional topologies recovered in the DM, CM, DP and CP analyses. [file BRV-92-521-s004.docx]

Appendix S5 – Additional topologies recovered in DM, CM, DP and CP analyses.

*Stem placentals*

In the DM, CM, DP, and CP analyses, *Protungulatum* was also most parsimoniously reconstructed as a non-placental eutherian, as in DF and CF. Where *Purgatorius* was left unconstrained, it was generally found as sister taxon to *Protungulatum,* immediately stemward of a paraphyletic Leptictida. The sole exception to this is the CM analysis, where *Protungulatum* was recovered crownward of Leptictida, with *Purgatorius* stemward. Where constrained as a relative of Primates, *Purgatorius* was found to be the sister taxon to the clade composed of *Adapis*, *Notharctus*, and *Plesiadapis*. The positions of Zhelestidae and Zalambdalestidae did not differ between any sets of constrained analysis.

*The Placental Root and Higher-Level Relationships*

All constrained analyses supported an Atlantogenata–Boreoeutheria division at the base of Placentalia. The sole difference in the higher-level relationships concerned the presence of extinct lineages on the stems of the superorders; the North American and East Asian clade of Palaeogene herbivores Arctostylopidae was found on the Atlantogenatan stem in the DP and CM analyses. Descriptions of unambiguous synapomorphies in the main text are those that are present across all analyses, not just DF and CF.

As with DF and CF, Eulipotyphla was supported as the most basal extant order within Laurasiatheria. In the DP and CP analyses, the same division between Euungulata and Chiroptera–Ferae as was found in the DF and CF analyses was recovered. However, in the DM and CM analyses, Perissodactyla was the next most basal clade to Eulipotyphla, and Chiroptera was closest to Artiodactyla, this latter relationship supported by the presence of a supraorbital process, an expanded tegmen tympani, and a keel on the posteroventral portion of the axis. The topology of Laurasiatheria had only little impact on the interpretation of several extinct groups, but the lack of resolution only perpetuates the current lack of understanding over higher-level Laurasiatherian interrelationships.

*Cimolestidae*While the position of *Gelastops* was consistent across analyses, the stem-eutherian group of cimolestids was found to be paraphyletic with respect to more crownward taxa in all analyses except CM, where it was a monophyletic sister clade to zalambdalestids.

*Leptictida*

The three leptictidans were not recovered as monophyletic in any analysis, but *Gypsonictops* and *Leptictis* were found to be sister taxa in all analyses. Contrasting with CF and DF, this was sister to a Placentalia–*Protungulatum* clade in CM, DM, CP, and DP analyses. *Prodiacodon* was found as a stem Atlantogenatan in CP and DM analyses, with an uncertain position near the placental root in DP and CM analyses.

*‘Condylarths’*
Despite use of disparate constraints, there were several points of consistency across all or most analyses in the positions of the various ‘condylarth’ clades. For example, the positions of Phenacodontidae and Pleuraspidotheriidae were consistent in all constrained analyses.

In the CM analysis, Apheliscidae was found to be sister to Eulipotyphla, and to Euungulata in the DP analysis. Besides these two exceptions, both CP and DM analyses returned the same result as CF and DF – sister to Scrotifera. Hyopsodontids were, in CM and DM, found as sister to the Phenacodontidae–Perissodactyla clade. In CP and DP, they were sister taxa to Artiodactyla, as in CF and DF. The exception for the position of Periptychidae and Pantodonta was CM, in which they were not more closely related to the Ferae–Chiroptera clade, but formed a polytomy with the perissodactyl stem and with the remainder of Laurasiatheria. Arctocyonidae differed from the CF and DF analyses only in that it was found to be diphyletic in DM, with triisodontids found to be stem carnivorans, and the remaining arctocyonids sister to the larger Carnivora–Creodonta clade, while in DP it was resolved as the monophyletic sister taxon to Ferae.

*Other Palaeocene taxa*

There were no large differences among constraints with respect to the Creodonta–Carnivora or the Palaeanodonta–Pholidota relationships.

*Protolipterna* was also resolved alongside archaic dichobunid artiodactyls in the DP, CM, CF and CP analyses, but near chriacid arctocyonids in DM. *Simpsonotus*, however, had a position that was less consistent, being found next to Palaeanodonta in CM and CP, but on the atlantogenatan stem with Arctostylopidae in DP, on the chiropteran stem with Arctostylopidae in CF, and close to Artiodactyla in DM.
